# Multicolor fluorescence activated cell sorting to generate humanized monoclonal antibody binding seven subtypes of BoNT/F

**DOI:** 10.1371/journal.pone.0273512

**Published:** 2022-09-01

**Authors:** Yongfeng Fan, Zhengda Sun, Fraser Conrad, Weihua Wen, Lequn Zhao, Jianlong Lou, Yu Zhou, Shauna Farr-Jones, James D. Marks

**Affiliations:** Zuckerberg San Francisco General Hospital and Trauma Center, Department of Anesthesia and Perioperative Care, University of California, San Francisco, San Francisco, CA, United States of America; National Research Council Canada, CANADA

## Abstract

Generating specific monoclonal antibodies (mAbs) that neutralize multiple antigen variants is challenging. Here, we present a strategy to generate mAbs that bind seven subtypes of botulinum neurotoxin serotype F (BoNT/F) that differ from each other in amino acid sequence by up to 36%. Previously, we identified 28H4, a mouse mAb with poor cross-reactivity to BoNT/F1, F3, F4, and F6 and with no detectable binding to BoNT/F2, F5, or F7. Using multicolor labeling of the different BoNT/F subtypes and fluorescence-activated cell sorting (FACS) of yeast displayed single-chain Fv (scFv) mutant libraries, 28H4 was evolved to a humanized mAb hu6F15.4 that bound each of seven BoNT/F subtypes with high affinity (K_D_ 5.81 pM to 659.78 pM). In contrast, using single antigen FACS sorting, affinity was increased to the subtype used for sorting but with a decrease in affinity for other subtypes. None of the mAb variants showed any binding to other BoNT serotypes or to HEK293 or CHO cell lysates by flow cytometry, thus demonstrating stringent BoNT/F specificity. Multicolor FACS-mediated antibody library screening is thus proposed as a general method to generate multi-specific antibodies to protein subtypes such as toxins or species variants.

## Introduction

The immune system generates antibodies that bind to epitopes with high specificity allowing the host to recognize and eliminate pathogens. In response to this immune pressure, pathogens can generate diversity and co-evolve to evade neutralization. Monoclonal antibodies (mAbs) with cross-immunity can be isolated that bind and neutralize multiple serotypes and subtypes of toxins [1], heterologous strains of bacteria [2], or different strains of viruses [3–6]. Such mAbs typically bind conserved epitopes that are shared to some extent between variants. As a result, generally the greater the sequence variation between variants, the greater the challenge in isolating cross-reactive mAbs.

Generating neutralizing antitoxins for botulism exemplifies the challenge of antigenic variation. Botulism is caused by botulinum neurotoxin (BoNT), the most poisonous substance known [7]. BoNT serotypes [8] are defined by their neutralization by serotype-specific antitoxin; an antitoxin against one serotype will not neutralize another serotype [9]. For six serotypes (A-F), there exist multiple subtypes, which vary at the amino acid level by a few percent to up to 36% for BoNT/F [10–12]. Sequence differences within subtypes result in changes in surface epitopes that can cause a reduction in mAb or antitoxin potency [13, 14].

As an alternative to equine derived antitoxin [15], highly potent human mAb-based antitoxins composed of three mAbs [13, 16–18] are being developed for serotypes A, B, C, D, E, F, and G with the most advanced (serotypes A, B, C, D, and E) having completed Phase 1 human testing [19–22]. The three mAbs bind non-overlapping epitopes on the BoNT molecule and elicit rapid clearance from the circulation and high potency [23, 24].

The BoNT molecule is composed of two peptide chains covalently linked through a disulfide bond: a light chain (LC) that contains the Zn^2+^-dependent endopeptidase, and a heavy chain (HC) that consists of a receptor-binding domain (H_C_) and translocation domain (H_N_). BoNT/F includes nine subtypes (BoNT/F1-F9) with amino acid differences as high as 36.2% (BoNT/F5 vs. F7) [12, 25]. The light chain of BoNT/F5 differs from that of other BoNT/F subtypes by greater than 50% in amino acid sequence [12]. The low homology among the subtypes presents a particularly significant challenge for the development of mAbs that bind all BoNT/F subtypes. In contrast, the greatest amino acid sequence difference between subtypes of BoNT/A is 15.6%, BoNT/B is 7.1%, BoNT/C is 24.2%, BoNT/D is 23.5% and BoNT/E is 11.3% [26]. No subtypes have been reported for the BoNT/G serotype.

We previously reported three murine IgG mAbs (6F5.1, 6F11 and 6F13) that bound to seven BoNT/F subtypes (F1-F7) with non-overlapping epitopes and that potently neutralized BoNT/F1 in mice [13]. The mAbs had high affinity to BoNT/F1 (K_D_, 1.47–327 x 10^−12^ M) and lower affinity to other subtypes (K_D_, 204–22,150 x 10^−12^ M), leading to 5-50-fold decreased protection against challenge with BoNT/F2, F4, and F7 toxins. To generate an additional lead mAb with broad BoNT/F specificity, we report here the molecular evolution of mAb 28H4 [13] using yeast displayed mutant scFv libraries. Because of the challenges of improving mAb affinity to all BoNT/F subtypes by labeling with a single BoNT/F subtype, we developed an approach whereby yeast displayed scFv libraries were simultaneously stained with up to five different BoNT/F subtypes labeled with different fluorophores prior to sorting. We believe this is a general approach that can be used to select cross-reactive mAbs.

## Materials and methods

### 1. Materials

Yeast, bacterial strains, and cell lines: *Saccharomyces cerevisiae* strain EBY100 was used for BoNT/F1 fragment display, single-chain variable fragment (scFv) display and scFv library construction. The *Escherichia coli* (*E*. *coli*) strain DH5α was used for subcloning and plasmid preparation and the TG1 strain was used for soluble single-chain antibody (scFv) expression. The cell line Chinese hamster ovary (CHO) was used for immunoglobulin G (IgG) expression. CHO cells and human embryonic kidney 293 (HEK293) cells were also used for cell lysate preparation used for polyspecificity tests.

Media: the yeast peptone dextrose (YPD) medium was used for EBY100 growth, the selective growth dextrose casamino acids media (SD-CAA), for pYD4-transformed EBY100 selection, and the selective growth galactose casamino acids media (SG-CAA), for induction of scFv expression. 2xYT was used for DH5α and TG1 growth.

Toxins, toxin fragments, and antibodies: The pure holotoxin BoNT/F1 was purchased from Metabiologics Inc. (Madison, WI). The crude toxins BoNT/F2 and F4 were provided by United States Army Medical Research Institute of Infectious Diseases (USAMRIID, Fort Detrick, MD). BoNT/F subtypes and BoNT/F5 fragments were expressed in BL21 [13] and were conjugated with different fluorescent labels using the Easylink antibody conjugation kit (Abcam, Burlingame, CA). Mouse anti-SV5 antibody was purified from a hybridoma cell line. BoNT/F IgG1 antibodies were made in CHO cells as described [17]. All the secondary antibodies including Phycoerythrin (PE) or Allophycocyanin (APC)-conjugated goat anti-human Fc, goat anti-mouse Fc and goat anti-human F(ab) were purchased from Jackson ImmunoResearch Laboratories (West Grove, PA).

### 2. scFv library construction

**Fig 1:**
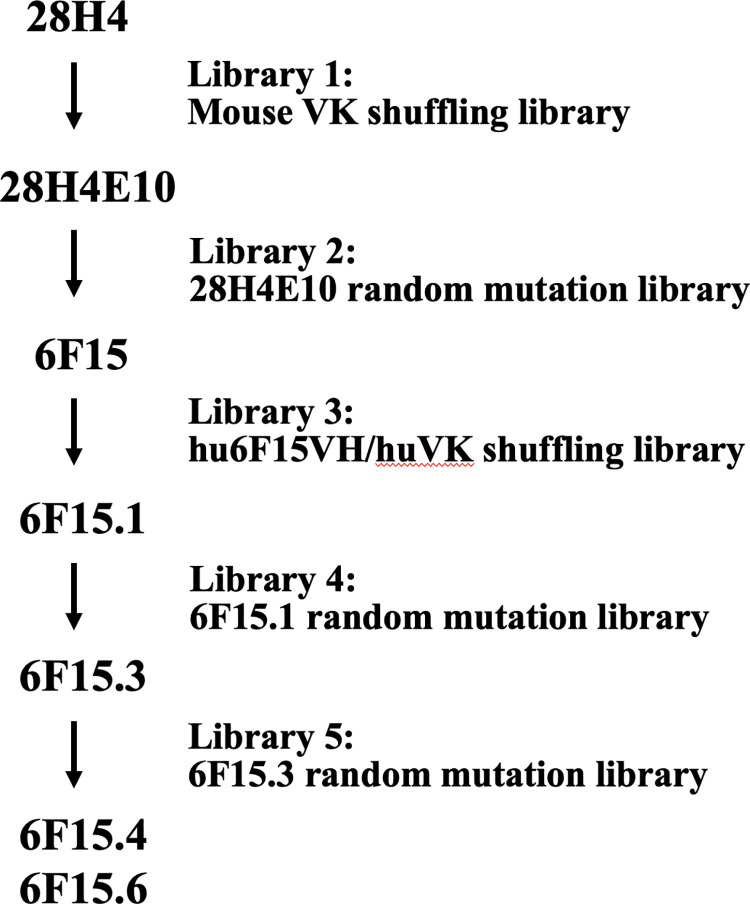
Outline of mAb 28H4 evolution. mAb 28H4 was evolved to mAb hu6F15.4 with four sequential intermediates (28H4E10, 6F15, hu6F15.1, and hu6F15.3) from five designed single-chain variable fragment (scFv) libraries including two light chain shuffling libraries and three random mutation libraries. MAbs hu6F15.4 and hu6F15.6 showed cross-reactivity to seven BoNT/F subtypes.

The parental antibody, 28H4 bound the subtypes of BoNT/F1, F2, F4 and F6, but did not bind BoNT/F2, F5 or F7 (S1 Fig). To achieve binding to all the 7 BoNT/F subtypes, five scFv libraries were constructed to evolve mAb 28H4. The most cross-reactive individual scFv from each round of evolution served as the input for the next round of evolution. An overview of the strategy and the five libraries used is summarized below and in Fig 1.

**Library One**: Light chain shuffling of murine scFv 28H4: A murine V_K_ library was constructed by cloning a murine V_K_ cDNA repertoire amplified from BoNT/F and BoNT/F H_C_ immunized mouse spleen into the vector pYD4 [27]. The recombinant pYD4-V_K_ repertoire was prepared with a plasmid Max Prep kit (Qiagen, Redwood City, CA) and digested with the restriction enzymes BssHII and NotI. The 28H4 V_H_ gene was amplified with a high-fidelity enzyme PicoMaxx (Agilent Technologies Inc., Santa Clara, CA) from the pYD4-scFv containing the parental scFv gene with gap tailed primers homologous with the pYD4 vector. Finally, the gap-tailed V_H_ gene and the digested pYD4-V_K_ library were co-transformed into EBY100 yeast cells with Lithium acetate as described previously [28] to form a light chain-shuffled library. The transformed EBY100 cells were cultured in 500 mL of SD-CAA at 30°C for 48 hours and then were induced in the medium SG-CAA at 18°C for 48 hours with shaking. In parallel, cells (10 μL) were plated on SD-CAA plates and cultured at 30°C for 48 hours for determination of library size. After sorting for improved cross-reactivity, the output of step 1 was scFv 28H4E10.

**Library Two:** Random mutation of scFv 28H4E10. A library of scFv 28H4E10 random mutants was constructed in pYD4 using error-prone PCR, cloned into pYD4, transformed, and induced as described above and previously [13]. After sorting for improved cross-reactivity in step 2, the scFv 6F15 was identified.

**Library Three:** Humanization of scFv 6F15 by light chain shuffling: A human V_K_ library was constructed by cloning a human V_K_ cDNA repertoire from BoNT/A, B, C, D, and E toxoid-immunized human donors into the vector pYD4 [27]. The recombinant pYD4-V_K_ repertoire was prepared with a plasmid Max Prep kit (Qiagen, Redwood City, CA) and digested with the restriction enzymes BssHII and NotI. The humanized 6F15 VH gene (hu6F15VH) was designed empirically based on framework homology, replacing the mouse amino acids that were anticipated to not affect the structure or binding affinity of the antibody with human ones. Briefly, the VH gene of 6F15 was first aligned with the human VH genes in the IMGT database [29]. The most homologous human VH was selected and the three CDR loops of 6F15 were inserted into the human frameworks. Then the parental murine 6F15 VH gene was aligned with the humanized VH gene comprising human frameworks and the 6F15 murine VH CDRs to identify amino acids in the frameworks that were different between the murine and humanized VH genes. Residues in the Vernier zone [30] or those involved in IgG folding and VH-VL packing [31, 32] were not changed from the murine to the human sequence, as we expected the structure or affinity would change significantly. Any other residues different between the mouse and human VH frameworks were replaced with human amino acids. Lastly, the humanized VH gene (huVH) was synthesized and amplified with a high-fidelity enzyme PicoMaxx (Agilent Technologies Inc., Santa Clara, CA) with gap-tailed primers homologous with the pYD4 vector. The gap-tailed humanized V_H_ gene and the pYD4-V_K_ library were co-transformed into EBY100 yeast cells with Lithium acetate as described previously [28] to form a light chain-shuffling library. After sorting for improved cross-reactivity, the output of step 3 was scFv hu6F15.1.

**Library Four:** Random mutation of hu6F15.1. A library of scFv hu6F15.1 random mutants was constructed in pYD4 using error-prone PCR, cloned into pYD4, transformed, and induced as described above and previously [13]. After sorting for improved cross-reactivity, the output of step 4 was scFv 6F15.3.

**Library Five:** Random mutation of hu6F15.3. A library of scFv hu6F15.3 random mutants was constructed in pYD4 using error-prone PCR, cloned into pYD4, transformed, and induced as described above and previously [13]. After sorting for improved cross-reactivity, the output of step 5 was scFv 6F15.4.

### 3. Library sorting

**Fig 2:**
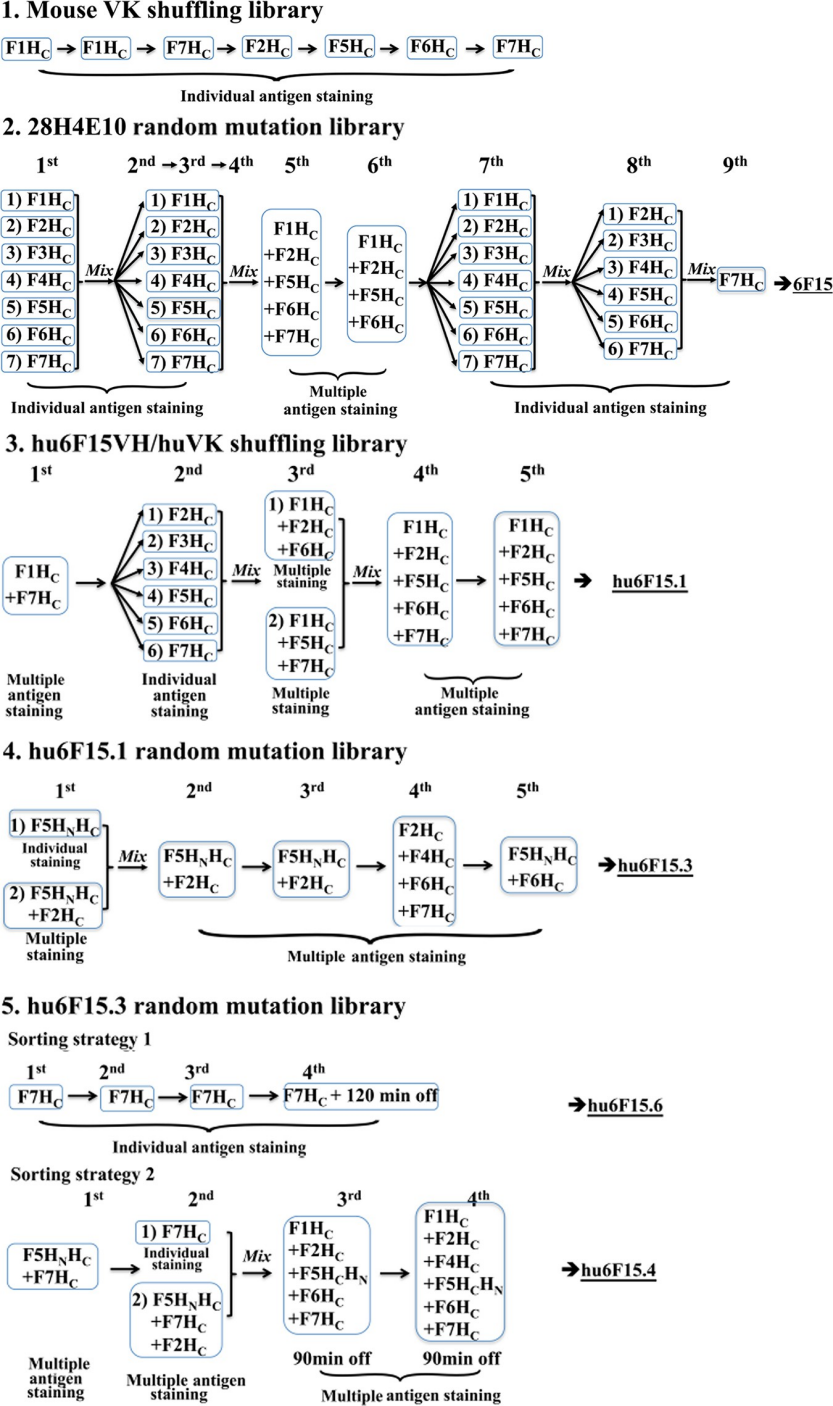
The detailed sorting strategies of each library. Each library was sorted using FACS at least four rounds using various strategies. Based on the different binding profile of the parental mAbs, libraries were stained with different strategies, including sequentially changed BoNT/F subtype, multiple BoNT/F subtype co-staining (multicolor FACS), or wild type mAb competence (off rate screen). The colonies from last round of sorting were plated on SD-CAA plates for further characterization including DNA sequence analysis and K_D_ and *k*_*off*_ measurements. The labels 1^st^– 9^th^ indicate the round of staining and sorting. Details of staining and sorting conditions are provided in the methods. Library 1. Light chain shuffling of murine scFv 28H4. The library was stained with the indicated individual BoNT/F subtype and sorted for six rounds. This cycle of molecular evolution yielded the scFv 28H4E10. Library 2. Random mutation of scFv 28H4E10. Nine rounds of sorting were performed on the 28H4E10 scFv library. For the first four rounds, aliquots of the library were individually stained with one of the seven BoNT/F H_C_ subtypes and sorted. The individual yeast outputs from sorting were combined and used for the next round of staining and sorting. For the 5^th^ and 6^th^ round of staining and sorting, BoNT/ F1, F2, F5, F6, and F7 HC (Round 5) or BoNT/F1, F2, F5, and F6 H_C_ (Round 6) labeled with unique fluorophores were mixed and used to stain the input yeast. Rounds 7 and 8 were performed as described for Rounds 1–4 and Round 9 was performed after staining with BoNT/F7 H_C_. This cycle of molecular evolution yielded the scFv 6F15. 3. Library 3. Humanization of scFv 6F15 by light chain shuffling. Five rounds of sorting were performed. For the first round of sorting, the library was stained with a mixture of BoNT/F1 and BoNT/F7 HC labeled with unique fluorophores. For Round 2, the aliquots of the output yeast from Round 1 were individually sorted with one of the indicated BoNT/F HC subtypes and the output from the individual sorting combined. For Rounds 3, 4, and 5, Yeast were stained with a mixture of the indicated BoNT/F HC subtypes labeled with unique fluorophores. This cycle of molecular evolution yielded the scFv hu6F15.1. 4. Library 4. Random mutation of hu6F15.1. Five rounds of sorting were performed. For the first round of staining and sorting, library aliquots were individually stained with either BoNT/F5 HC or a mixture of BoNT/F5 and BoNT/F2 HC and sorted. Sorting outputs were combined and used for the next round of sorting. For Rounds 2–5, yeast were stained with a mixture of the indicated BoNT/F H_C_ subtypes labeled with unique fluorophores. This cycle of molecular evolution yielded the scFv hu6F15.3. 5. Library 5. Random mutation of hu6F15.3. This library was stained and sorted using two approaches. In the first approach, five rounds of sorting were performed. In each round of staining, yeast were stained with a mixture of the indicated BoNT/F H_C_ subtypes labeled with unique fluorophores. A 90-minute period of dissociation of labeled antigen after staining was used during the 3^rd^ and 4^th^ rounds of selection prior to sorting. This cycle of molecular evolution yielded the scFv hu6F15.4. For the second approach, yeast were stained with only BoNT/F7 H_C_ for each round of sorting. A 120-minute period of dissociation of labeled antigen after staining was used during the 4^th^ round of selection prior to sorting. This cycle of molecular evolution yielded the scFv hu6F15.6.

The staining and sorting conditions varied for each of the five scFv yeast displayed libraries. For single antigen sorting, libraries were stained and isolated as described previously. Sort gates were set to collect only yeast that bound antigen and that displayed scFv as quantitated using an anti-SV5 monoclonal antibody [13] as shown in detail in Fig 2. For multicolor sorting, libraries were co-stained with three or five different fluorophore-conjugated antigens (F1H_C_-APCcy7, F2H_C_-PEcy7, F5 H_C_ -Alexa 647, F6 H_C_ -PercpCy5.5, F7 H_C_ -Alexa 488). Four parameters were adjusted in a series of experiments done to determine what conditions to use to stain libraries for sorting (1) the antigen concentration relative to scFv concentration; (2) the relative concentration of each antigen; (3) the total antigen amount; and (4) the total reaction volume such that the antigen amount always exceeded the amount of scFv present by 5-10-fold. Specifically:

1. The antigen concentration was adjusted to be 5-10-fold greater than yeast-displayed scFv molecules in order to saturate binding.
2. To determine the concentration of each antigen to use for library staining, the K_D_ of the parental antibody for each fluorophore-conjugated antigen was measured by flow cytometry. The concentration around the K_D_ of each antigen was used for library staining.
3. The total number of moles of each antigen in the solution was approximately equal to avoid bias in binding due to affinity differences.
4. The reaction volume used was determined based on the total scFv amount and the concentration of antigen with the lowest K_D_ value. Thus, for the antigens with high affinity, a higher concentration than K_D_ could be applied to control the incubation volume.

Libraries were stained at room temperature for one hour with different antigen combinations using a 10-20-fold excess of yeast cell over library size using a yield mask set to the maximum value for the first round to achieve more rapid sorting. Yeast cells binding multiple BoNT/F antigens were collected by intersecting the sort gates set on each of the individual dot-plots of antigen-binding versus yeast display level. Briefly, binding of library yeast after staining was displayed in a dot-plot, in which the x-axis indicated scFv expression level detected with PE-conjugated anti-SV5 IgG and the y-axis showed binding signals of one of the BoNT antigens used for staining. For each dot-plot, a sort gate was set to capture yeast that both bound BoNT/F antigen and were displaying scFv. These individual antigen sort gates were then intersected to capture and collect only those yeast contained in all of the sort gates and that bound multiple BoNT/F antigens. The same gating strategy was used in all multicolor sorting processes.

### 4. Mab affinity determination

Yeast displayed scFv affinity for BoNT was determined using serially diluted BoNT/F H_C_ fragments (100, 20, 4, 0.8, 0.16 nM) or a control with no BoNT/F H_C_ as previously described [13]. BoNT H_C_ binding was detected by staining with IgG 6F3 [13] (for BoNT/F7 H_C_, 1 μg/mL) or hu6F8 [13] (for BoNT/H_C_ of the other 6 subtypes, 1μg/mL) followed by PE-labeled goat anti-human IgG (1 μg/mL, Jackson Immuno Research Inc.) and Alexa-647 labeled anti-SV5 IgG (1 μg/mL). Mean fluorescence intensity (MFI) was measured using flow cytometry and K_D_ values were calculated as described previously [13].

For some yeast displayed scFv, *k*_*off*_ was also measured. Yeast displayed scFv were incubated with 50 nM of a BoNT/F H_C_ domain for one hour at room temperature. Yeast were then washed with cold FACS buffer three times to remove the unbound BoNT/F H_C_ and yeast resuspended in 200 μL of FACS buffer containing 100 nM of an IgG binding the same epitope as the yeast displayed scFv to bind the unbound and dissociated BoNT/F H_C_ and prevent rebinding. Samples were taken at times 0, 15, 30, 60, 120, 180, and 240 minutes. Each sample was incubated with 1 μg/mL of 6F3 or hu6F8 IgG-APC and SV5-488 and MFIs were determined by using flow cytometry. The half-life of antigen/scFv binding on the yeast surface was calculated based on the formula:

$$t½=\log(\frac{\mathrm{MFI}\;\mathrm{at}\;T=0}{\mathrm{MFI}\;\mathrm{at}\;\mathrm{elapsed}\;\mathrm{time}}).\;\mathrm{Then}\;k_{\mathrm{off}}\;\mathrm{was}\;\mathrm{calculated}\;\mathrm{based}\;\mathrm{on}:\;k_{\mathrm{off}}=\ln\;2/t_{½}.$$

K_D_ values of IgG’s in solution were measured using a KinExA flow fluorimeter as described previously [13, 17]. BoNT/F holotoxin or BoNT/F H_C_ domains were mixed with serially diluted IgG from less than 0.1-fold to greater than 10-fold above the K_D_ value. The samples were incubated at room temperature until equilibrium was reached. Samples were then passed over a flow cell that was packed with a N-hydroxysuccinimide-activated Sepharose 4 Fast Flow beads (GE Healthcare, Little Chalfont, UK) conjugated with the same IgG the K_D_ value of which was being measured. Finally, the bead-captured toxin was detected with another mAb that bound to a different epitope than the IgG being measured, either 6F3 or hu6F8. K_D_ values were calculated using KinExA Pro software (Sapidyne Instruments, Boise, ID, USA).

### 5. Thermostability assays

The melting temperature (Tm) of IgGs was measured with differential scanning fluorimetry (DSF) as described with modification [33]. Briefly, 1 μM of IgG was incubated with SYPRO orange dye (1:400, Invitrogen) in 40 μl of final volume with PBS in a DMSO-resistant 96-well plate (Thermo Fisher). Fluorescent intensity was measured at 610 nM using the quantitative real-time PCR instrument (Stratagene Mx3000P) with temperature scanning from 25°C to 95°C at 1°C min^−1^. Tm values were calculated using the Prism v6.0 software (Graphpad Software, La Jolla, CA, USA).

### 6. Polyspecificity assays

The polyspecificity of mAbs was measured using flow cytometry. First, the mAbs were tested for binding to the other BoNT serotypes. Yeast-displayed scFv were incubated with 50 nM of BoNT/A1, B1, C, D/C, E3 or G, or 50 nM of BoNT/F1 as a positive control for one hour at room temperature. Binding was detected by incubating with 1 μg/mL at 4°C for one hour of a combination of ING2 IgG (for BoNT/A1 detection), B6.1 IgG (for BoNT/B1 detection), 4C2/4C4.2 IgG (for BoNT/C and D/C detection), 4E17.1 IgG (for BoNT/E3 detection) and 7G1.1/7G3.1 IgG (for BoNT/G detection) or hu6F13.4 (for BoNT/F1 detection). After incubation, yeast were washed and incubated with PE-conjugated goat anti-human IgG (1 μg/mL) at 4°C for one hour and 1 μg/mL of SV5-Alexa-647. Yeast cells were washed with FACS buffer three times and were then analyzed for BoNT binding by using flow cytometry. MAbs were also analyzed for binding to mammalian cell lysates. Biotin-labeled cell lysate from CHO cells and HEK293 cells were prepared as described [34], diluted ten-fold, and incubated with yeast-displayed scFv at room temperature for one hour. The yeast cells were washed with FACS buffer three times and resuspended in FACS buffer containing PE-conjugated streptavidin (1 μg/mL) and SV5-647 (1 μg/mL) and incubated at 4°C for one hour. After washing three times with FACS buffer, the samples were analyzed by flow cytometry.

### 7. Epitope identification and fine epitope mapping

The fine epitope of 28H4, hu6F15.4 and hu6F15.6 were identified by isolation of BoNT/F1 H_C_ mutants that did not bind hu6F15.4 as previously described [27, 35]. Briefly, random mutations were introduced into the BoNT/F1 H_C_ gene by an error-prone PCR and the mutant repertoire cloned into the vector pYD4 and displayed on the surface of EBY100 yeast. Mutants with loss of hu6F15.4 binding were enriched through three rounds of FACS sorting by staining with hu6F15.4 and hu6F8. Yeast without hu6F15.4 binding but with hu6F8 binding were sequenced and the sequences were aligned to identify shared mutations, which were regarded as energetically important amino acids in the epitope. These amino acids, together with structurally adjacent residues on the BoNT/F1 H_C_ or F7 H_C_ surface were individually mutated to alanine and displayed on the yeast surface. Where the wild-type amino acid was alanine, the residue was changed to glycine. A range of concentrations of Fab fragments of 28H4, hu6F15.4 and hu6F15.6 were incubated with the BoNT/F H_C_ alanine mutants to determine the K_D_ for each alanine mutant. Experiments were performed in triplicate and the mean K_D_ values were obtained for calculation of the changes of Gibbs free energy (ΔΔG) to evaluate the contribution of each amino acid in the epitope as previously described [27, 35].

### 8. Molecular modeling and BoNT/F residue numbering

The Alphafold model of BoNT/F (AF-A7GBG3) was used to model the location of the epitopes of 28H4 and derivatives hu6F15.4 and hu6F15.6 on the holotoxin surface. The BoNT/F1H_C_ crystal structure (PDB ID, 3FUQ) and a model of the BoNT/F7H_C_ were used to visualize the fine epitopes for mAbs 28H4 hu6F15.4 and hu6F15.6. The BoNT/F7H_C_ model was constructed based on the BoNT/F1 crystal structure (PDB ID, 3FUQ) using the server SWISS-MODEL [36]. The overall root-mean-square deviation (RMSD) Structural Distance Measure (SDM) and Q-score were calculated with UCSF Chimera to evaluate the models [37–39]. The RMSD was also calculated using the position of either backbone residues or all heavy atoms. Pymol 2.0 was used to view, edit, and show the epitopes on BoNT/F or BoNT/FH_C_. To visualize differences in the protein sequences of BoNT/F1-F9 impacting the epitopes of 28H4, hu6F15.4 and hu6F15.6, BoNT/F sequences were aligned by the alignment software Bioedit 7.2 (RRID:SCR_007361). The numbering system generated from the alignment was used to number the amino acids of BoNT/F1, BoNT/F7, and the other BoNT/F subtypes.

## Results

### 1. Molecular evolution of scFv 28H4 to bind seven BoNT/F subtypes

As previously reported, scFv 28H4 was isolated from a yeast displayed library constructed from a mouse immunized with BoNT/F1 H_C_ and boosted with BoNT/F1 holotoxin [13]. scFv 28H4 bound the H_C_ domain of BoNT/F as determined using yeast displayed BoNT/F1 domains (S1 Fig and Table 1) with significantly higher affinity for BoNT/F1 H_C_ (K_D_ on yeast surface, 10.88 nM) than to BoNT/F3, F4, or F6 (K_D_ on yeast surface 237 nM, 212 nM, and >250 nM respectively). There was no detectable binding to BoNT/F2, F5, or F7 when stained with 100 nM of antigen (S1 Fig). To sequentially evolve mAb 28H4 to bind seven BoNT/F subtypes with a K_D_ < 1 nM, rounds of molecular evolution were used (Figs 1 and 2). The most cross-reactive individual scFv from each round of evolution served as the input for the next round of evolution. scFv libraries for each round of evolution contained at least 2 x 10^7^ scFv variants. To optimize binding to the seven different BoNT/F subtypes, different sorting strategies were used including switching subtypes used for staining after each round, sorting separately library aliquots stained with different subtypes, and then combining the outputs for the next round of sorting or staining with multiple subtypes in a single sample by labeling subtypes with different fluorophores.

**Table 1:** K_D_ values of yeast displayed scFv mAbs as measured by using flow cytometry.

	K_D_ values (nM)						
	28H4	28H4E10	6F15	hu6F15.1	hu6F15.3	hu6F15.4	hu6F15.6
**F1HC**	10.88 ±5.03	1.10 ± 0.49	0.93 ± 0.22	1.63 ±1.09	0.996 ± 0.41	1.02 ± 0.65	0.70 ± 0.3
**F2HC**	No binding	63.73 ±10.64	47.12 ± 6.52	34.22 ± 2.89	23.67 ± 2.12	23.48 ± 3.46	50.79 ± 0.37
**F3HC**	237 ±69.9	14.64 ± 3.13	12.31 ±1.27	10.75 ± 2.02	11.44 ±1.65	14.18 ± 4.03	16.58 ± 0.66
**F4HC**	212 ±45.1	13.04 ± 2.87	15.60 ± 4.01	12.82 ± 2.89	12.69 ±1.75	15.21 ± 6.19	12.46 ± 3.05
**F5HC**	No binding	36.82 ±14.33	28.84 ± 3.56	7.82 ± 2.56	8.87 ±1.66	9.28 ±1.80	16.88 ±1.26
**F6HC**	>250	32.08 ±10.71	28.96 ± 6.19	17.18 ± 3.24	19.64 ± 0.55	13.84 ±3.36	30.21 ± 3.71
**F7HC**	No binding	>250	12.35 ± 0.96	1.36 ± 0.51	1.55 ± 0.50	1.51 ± 0.38	0.47 ± 0.29

No binding = mean fluorescent intensity no different than control when stained with 250 nM BoNT. K_D_ > 250 nM = unable to saturate binding at a BoNT/F concentration of 500 nM. Measurements used for K_D_ calculations are shown in S1 Data.

### Library One: Affinity maturation of murine scFv 28H4

**Fig 3:**
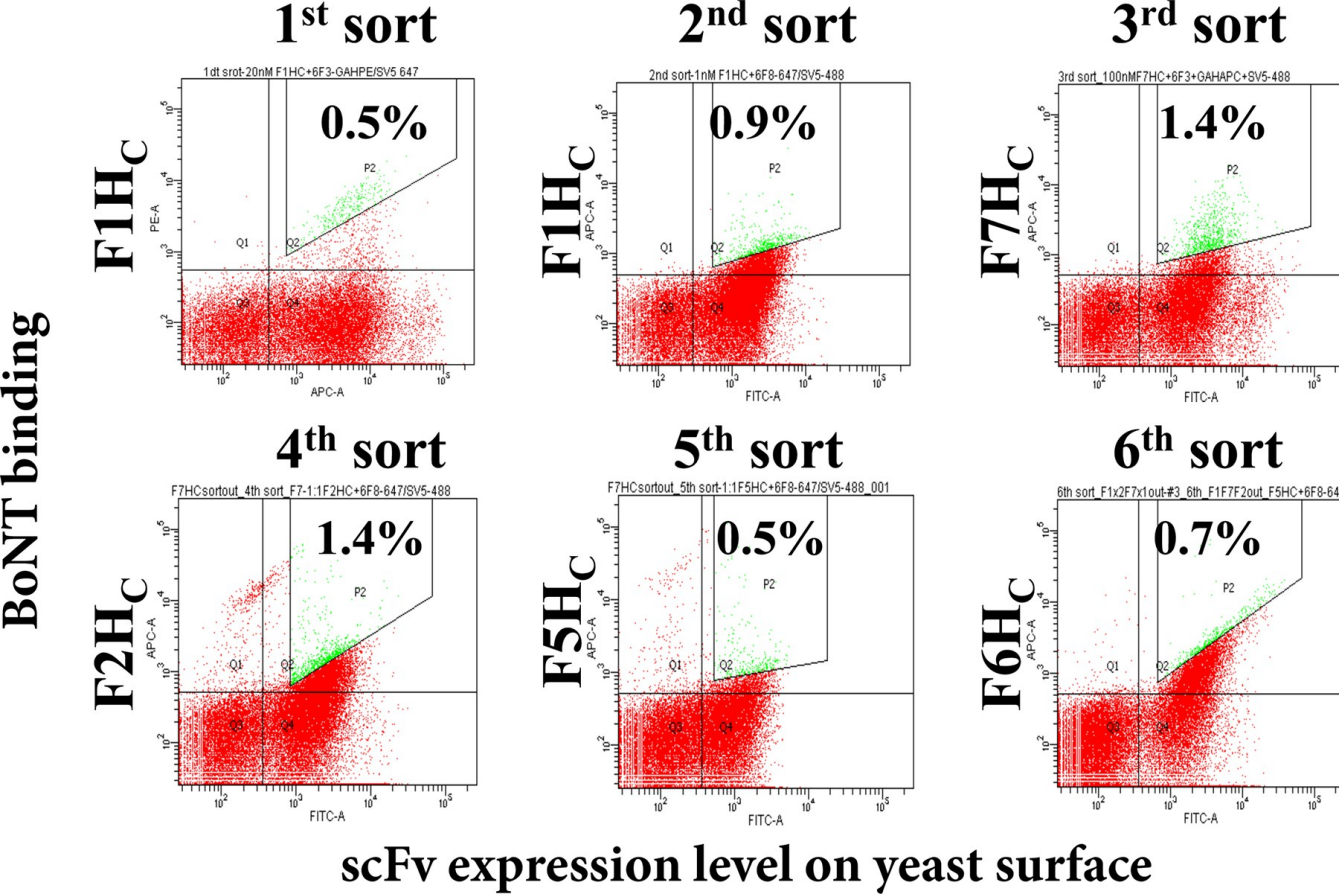
Dot plots of FACS of the mAb 28H4 scFv yeast displayed library stained with a single subtype for each round of sorting. The 28H4 library was sorted for six rounds sequentially staining with different BoNT subtypes BoNT/F1H_C_ (Rounds 1 and 2) BoNT/F7H_C_ (Round 3) BoNT/F2H_C_ (Round 4), BoNT/F5H_C_ (Round 5), and BoNT/F6H_C_ (Round 6). Each dot represents a single yeast with the BoNT subtype used for staining and the BoNT binding fluorescence shown on the y-axis and the level of scFv yeast surface display on the x-axis. The population gated for sorting and the percent of the total population sorted is shown with the gated yeast colored green.

A yeast displayed library of scFv 28H4 mutants was constructed where the 28H4 Vk light chain was replaced with a library of murine Vk light chains (light chain shuffling) [28]. The library was sorted for six rounds; for the first two rounds, the library was stained with BoNT/F1 H_C,_ and for rounds 3 through 6 the library was stained with BoNT/F7 H_C_ (round 3), BoNT/F2 H_C_ (round 4)_,_ BoNT/F5 H_C_ (round 5) and BoNT/F6 H_C_ (round 6) (Fig 3). After the last round of sorting, individual colonies were screened for binding to BoNT/F subtypes. The most cross-reactive scFv, 28HE10, had an at least 10-fold enhanced affinity to BoNT/F1H_C_ (K_D_, 1.10 nM vs. 10.88 nM), F3 H_C_ (K_D_, 14.64nM vs. 237 nM) F4 H_C_ (K_D,_ 13.4 nM vs. 212 nM) and F6 H_C_ (K_D_, 32.08 nM vs. >250 nM) (Table 1). 28HE10 also had measurable binding to BoNT/F2H_C_, F5H_C,_ and F7H_C_ (63.73 nM, 36 nM, and >250 nM respectively) whereas the parental scFv 28H5 had no measurable binding to these subtypes.

### Library Two: Affinity maturation of scFv 28H4E10

To further improve cross-reactivity, random mutations were introduced into the 28H4E10 scFv gene by using error-prone PCR [13]. The library was sorted for nine rounds, with rounds 5 and 6 performed by co-staining with BoNT subtypes labeled with different fluorophores (multicolor FACS). A more detailed description and visualization of multicolor staining and sorting is detailed below for Library Three. The final round of sorting was performed after staining with BoNT/F7. After the last round of sorting, individual colonies were screened for binding to BoNT/F subtypes. The resulting scFv, 6F15, had significantly improved affinity for BoNT/F7 H_C_ (K_D,_ 12.35 nM vs. >250 nM for 28H4E10) while retaining affinity similar to 28H4E10 for the other BoNT/F subtypes (Table 1).

### Library Three: Humanization of murine scFv 6F15

**Fig 4:**
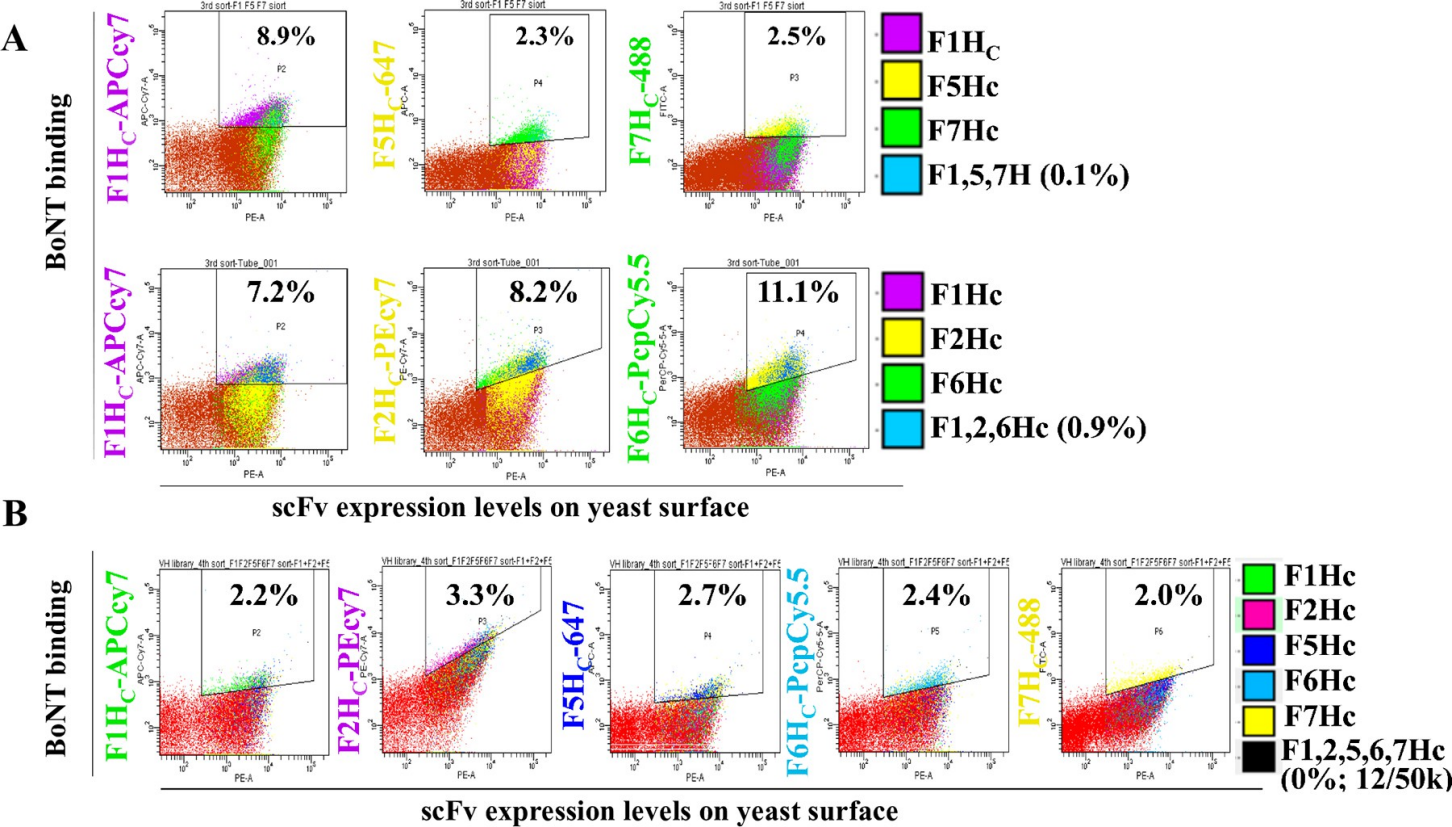
Dot plots of FACS of the mAb 28H4E10 scFv yeast displayed library using multi-color staining. **A.** For the third round of sorting, the scFv library was stained with a mixture of either BoNT subtypes BoNT/F1H_C_-APCcy-7, BoNT/F5H_C_-Alexa647, and BoNT/F7H_C_-Alexa488 or BoNT subtypes BoNT/F1H_C_-APCcy-7, BoNT/F2H_C_-PEcy7, and BoNT/F6H_C_-Pepcy5.5. Each dot represents a single yeast with the BoNT subtype used for staining and the BoNT binding fluorescence shown on the y-axis and the level of scFv yeast surface display on the x-axis. The population gated for sorting and the percent of the total population sorted is shown with the gated yeast colored according to the legend for each of the BoNT subtypes. For sorting the three gates were intersected and only yeast in all three gates (colored blue and with the percent of yeast in the intersected yeast indicated) were collected. **B.** For the fourth round of sorting, the scFv library was stained with a mixture of BoNT subtypes BoNT/F1H_C_-APCcy-7, BoNT/F2H_C_-PEcy7, BoNT/F5H_C_-Alexa647, BoNT/F6H_C_-Pepcy5.5, and BoNT/F7H_C_-Alexa488. Each dot represents a single yeast with the BoNT subtype used for staining and the BoNT binding fluorescence shown on the y-axis and the level of scFv yeast surface display on the x-axis. The population gated for sorting and the percent of the total population sorted is shown with the gated yeast colored according to the legend for each of the BoNT subtypes. For sorting the five gates were intersected and only yeast in all five gates (colored blue and with the percent of yeast in the intersected yeast indicated) were collected.

To support manufacturing and clinical development, 6F15 was humanized through sorting a library that consisted of a designed humanized V_H_ paired (shuffled) with a human V_K_ repertoire. For the first round of sorting, libraries were co-stained with BoNT/F1 and BoNT/F7, and for the second round of sorting, library aliquots were stained and sorted individually with the seven BoNT/F H_C_ subtypes to isolate colonies with improved affinity to each subtype. The sorted yeast cells were then combined for the next round of sorting with multicolor FACS staining to improve affinity to lower affinity subtypes while maintaining affinity to higher affinity subtypes. Yeast cells were stained with a combination of four labels, either BoNT/F1H_C_-APC-Cy7, F5H_C_-647, F7H_C_-488, and anti-SV5-PE or BoNT/F1H_C_-APC-Cy7, F2H_C_-PEcy7, F6H_C_-PerCP-Cy5.5, and SV5-PE. To collect yeast cells, for example, with binding to BoNT/F1, F5 and F7, three different dot-plots were generated plotting yeast scFv display level vs binding to BoNT/F1H_C_, BoNT/F5H_C_, or BoNT/F7H_C._ Sort gates were set on each of these populations and then intersected so that the cell sorter only collected yeast cells contained within all three sort gates and thus binding all three BoNT/F subtypes (Fig 4A). This sorting process was repeated for the yeast stained with BoNT/F1H_C_, BoNT/F2H_C_, and BoNT/F6H_C._ The two groups of sorted yeast cells were mixed for the next round of sorting, in which yeast cells were simultaneously stained with five different BoNT antigens including BoNT/F1H_C_-APC-Cy7, F2H_C_-PE-Cy7, F5H_C_-647, F6H_C_-PerCP-Cy5.5, F7H_C_-488, and SV5-PE, collecting the cell population (12 cells per 50,000 cells sorted) with F1H_C_, F2H_C_ F5H_C_, F6H_C,_ and F7H_C_ with the same gating strategy (Fig 4B). Analysis of multiple scFv after this final round of sorting yielded scFv hu6F15.1 consisting of a fully human V_K_ and a humanized V_H_. Compared with the murine 6F15, K_D_ values on yeast cells of Hu6F5.1 showed improved affinity to BoNT/F7H_C_ (12-fold, 1.36 nM vs. 12.35 nM), BoNT/F5H_C_ (3.7-fold, 7.82 nM vs. 28.84 nM) and BoNT/F6H_C_ (1.7-fold, 17.18 nM vs. 28.96 nM), while the affinity to BoNT/F1H_C_, F2H_C_, F3H_C_, and F4H_C_ remained relatively unchanged (Table 1).

Since the final therapeutic mAb format is IgG, and since we have observed variability between the affinity of yeast displayed scFv and IgG [13, 16, 17], we converted the parental scFv 28H4 and the humanized Hu6F5.1 to IgG and measured the IgG solution K_D_ to seven BoNT/F subtypes using flow fluorimetry in a KinExA. These studies confirmed that the parental 28H4 did not have a measurable affinity for BoNT/F2, F5, or F7. In contrast, the humanized and affinity matured Hu6F15.1 bound BoNT/F2, F5, or F7 with K_D_ values ranging from 0.6 to 3.76 nM and had 17-fold to 333-fold higher affinity for the other four BoNT subtypes (Table 2).

**Table 2:** K_D_ values of IgGs for F toxin domains as measured with KinExA (nM).

	K_D_ (nM) (95% confidence interval)				
	28H4	Hu6F15.1	hu6F15.3	hu6F15.4	hu6F15.6
**F1 holotoxin**	3.48 (4.17–2.89)	0.010 (0.016–0.005)	0.038 (0.042–0.034)	0.031 (0.035–0.025)	0.302 (0.389–0.201)
**F1 H**_**C**_	0.585 (0.716–0.395)	0.031 (0.037–0.025)	0.013 (0.014–0.011)	0.006 (0.007–0.004)	0.147 (0.181–0.102)
**F2 H**_**C**_	No binding	0.6 (0.624–0.542)	0.268 (0.285–0.236)	0.057 (0.068–0.048)	1.03 (1.06–0.985)
**F3 H**_**C**_	13.890 (14.96–12.24)	0.8 (0.856–0.733)	0.254 (0.275–0.233)	0.078 (0.084–0.73)	0.722 (0.854–0.607)
**F4 H**_**C**_	2.21 (2.57–1.82)	0.067 (0.096–0.043)	0.023 (0.044–0.004)	0.018 (0.019–0.014)	0.292 (0.338–0.198)
**F5 H**_**N**_ **H**_**C**_	No binding	0.551 (0.739–0.339)	0.37 (0.732–0.136)	0.23 (0.259–0.199)	5.57 (5.79–5.25)
**F6 H**_**C**_	12.10 (12.41–11.68)	0.600 (.714–0.383)	0.231 (0.239–0.214)	0.053 (0.055–0.051)	0.808 (0.885–0.716)
**F7 H**_**C**_	No binding	3.76 (4.04–3.50)	2.540 (4.12–1.15)	0.66 (0.969–0.307)	0.023 (0.03–0.164)

No binding = no signal detected with 250 nM of antigen. KinExA plots and K_D_ calculations are shown in S3 Data.

### Library Four: Affinity maturation of scFv Hu6F15.1

We target an affinity of less than 1 nM for lead therapeutic mAbs, which was not achieved for the BoNT/F7 subtype and barely achieved for the BoNT/F2, F3, and F5 subtypes. In addition, there was a 360-fold difference between the highest and lowest affinity subtype. To increase the affinity of Hu6F15.1 to BoNT/F2, F3, and F7, a random mutation library based on hu6F15.1 scFv was generated. The library was sorted with BoNT/F2H_C_-PE-Cy7 and F5HC-6F5.4-PE for three rounds, then with BoNT/F2H_C_-PE-Cy7, F4 holotoxin-6F5.4-PE, F6H_C_-percypCy5.5, and F7H_C_-488 for two rounds, and then with F5H_N_H_C_-6F5.4-PE and F6H_C_-PerCP-Cy5.5 for one round. Analysis of multiple scFv after the final round of sorting yielded scFv hu6F15.3. Although the K_D_ values on the surface of yeast measured with flow cytometry did not show significant improvement (Table 1), the IgG K_D_ measured by using flow fluorimetry showed significant improvement in the affinity of hu6F15.3 to BoNT/F2H_C_, BoNT/F3H_C_, BoNT/F5H_C_ and BoNT/F6H_C_ (K_D_, 230 pM-370 pM) and similar binding affinity to BoNT/F1H_C_ (K_D_, 12.83 pM) and BoNT/F4H_C_ (K_D_, 23.39 pM) (Table 2). The affinity to BoNT/F7H_C_, however, was still relatively low compared to other subtypes (K_D_, 2540 pM).

### Library Five: Affinity maturation of scFv Hu6F15.3

To improve the affinity of Hu6F15.3 to BoNT/F7, another random mutation library based on hu6F15.3 scFv was constructed. Two different sorting strategies were compared. In the first strategy, the scFv library was selected using BoNT/F7H_C_ for four rounds, using 1 nM BoNT/F7 H_C_ for the first two rounds, and 2 nM BoNT/F7H_C_ for the third and fourth round with a 120-minute dissociation step during the fourth round. Analysis of multiple scFv after the final round of sorting yielded scFv hu6F15.6 that had a K_D_ for BoNT/F7 on the surface of yeast. This was three-fold higher than the K_D_ of hu6F15.3 (0.47 nM vs. 1.55 nM, Table 1). However, hu6F15.6 had an approximate two-fold decrease in affinity for BoNT/F2H_C_, F5H_C_ and F6H_C_ (50.79 nM vs. 23.67 nM for BoNT/F2H_C_, 16.88 nM vs. 8.87 nM for F5H_C_, 30.21 nM vs. 19.64 nM for F6H_C_, Table 2). In the IgG format, hu6F15.6 had a 110-fold increase in affinity for BoNT/F7 H_C_ compared to Hu6F15.3 (22.85 pM vs. 2540 pM) (Table 2), however, the affinity for the other BoNT/F subtypes decreased by 3.5 to 15-fold indicating that a selection strategy using a single subtype had a negative impact on the binding affinity to other subtypes.

In the second sorting strategy, multicolor FACS was used to select the hu6F15.3 library to better maintain binding affinity to all BoNT/F subtypes. The first two rounds of sorting were done after staining with BoNT/F5 and BoNT/F7 (round 1) and BoNT/F5, BoNT/F7, and BoNT/F2 (round 2). The final two rounds of sorting were done after staining with BoNT/F1, BoNT/F2, BoNT/F5, BoNT/F6, and BoNT/F7 (round 3) and with BoNT/F1, BoNT/F2, BoNT/F4 BoNT/F5, BoNT/F6, and BoNT/F7 with a 90-minute dissociation step during these rounds. Analysis of multiple scFv after the final round of sorting yielded scFv hu6F15.4 that had a K_D_ value for BoNT/F7 on the surface of yeast. This was approximately the same as hu6F15.3 (1.51 nM vs. 1.55 nM, Table 1) but unlike hu6F15.6, the affinity of hu6F15.4 for the other BoNT/F subtypes also was unchanged compared to hu6F15.3 (Table 1). In the IgG format, hu6F15.4 had a 3.8-fold increase in affinity for BoNT/F7 H_C_ (660 pM vs. 2540 pM) (Table 2), and the affinity for the other BoNT/F subtypes also increased by 1.6 to 4.7-fold indicating that a selection strategy using multiple subtypes better-maintained subtype cross-reactivity (Table 2).

### 2. Thermostability and specificity of engineered mAbs

**Fig 5:**
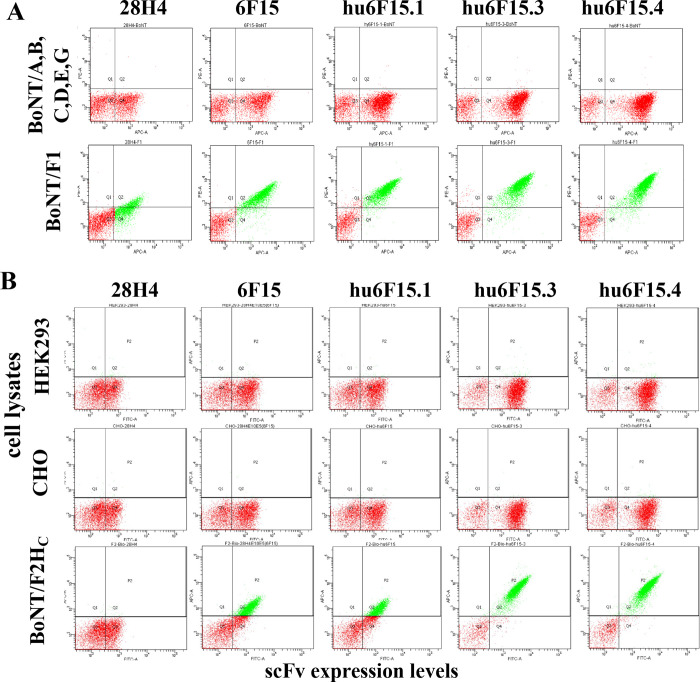
Specificity of mAbs with broadened specificity and improved affinity. **A.** Yeast displaying the indicated scFv were stained with either an equimolar mixture of BoNT serotypes A, B, C, D, E, and G or with BoNT/F1. Each yeast displayed scFv bound BoNT/F1 but did not bind the mixture of the other six serotypes. **B.** Yeast displaying the indicated scFv with stained with either biotinylated HEK293 or Chinese Hamster Ovary (CHO) cell lines or with biotinylated BoNT/F2H_C_ as a positive control. Binding was detected using streptavidin-PE. scFv did not bind HEK or CHO cell lines but did bind BoNT/F2 in proportion to their affinity.

The melting temperature (T_m_) of these mAbs was determined with differential scanning fluorimetry (DSF). By this method, the T_m_ of the hu6F15.4 was measured as 75.0°C (95% CI, 74.1°C-74.8°C) showing high thermostability, although 6°C lower than the parental mAb 28H4 (81.5°C, 95% CI, 81.5°C—81.6°C), DSF plot and raw data are shown in S2 Data. When tested with serotypes of BoNT/A1, B1, C, D/C, E3, and G, the mAb variants did not show any binding to these BoNT serotypes at concentrations as high as 50 nM (Fig 5A). mAb specificity was also evaluated by staining with biotin-labeled cell lysates from CHO cells and HEK293 cells, indicating that none of the mAb variants showed any binding to the cell lysates (Fig 5B). The data indicated that the increase in the cross-reactivity to BoNT/F subtypes 1–7 did not cause polyspecificity to proteins either from humans or hamster.

### 3. Visualizing the structural basis of increased subtype cross-reactivity

**Fig 6:**
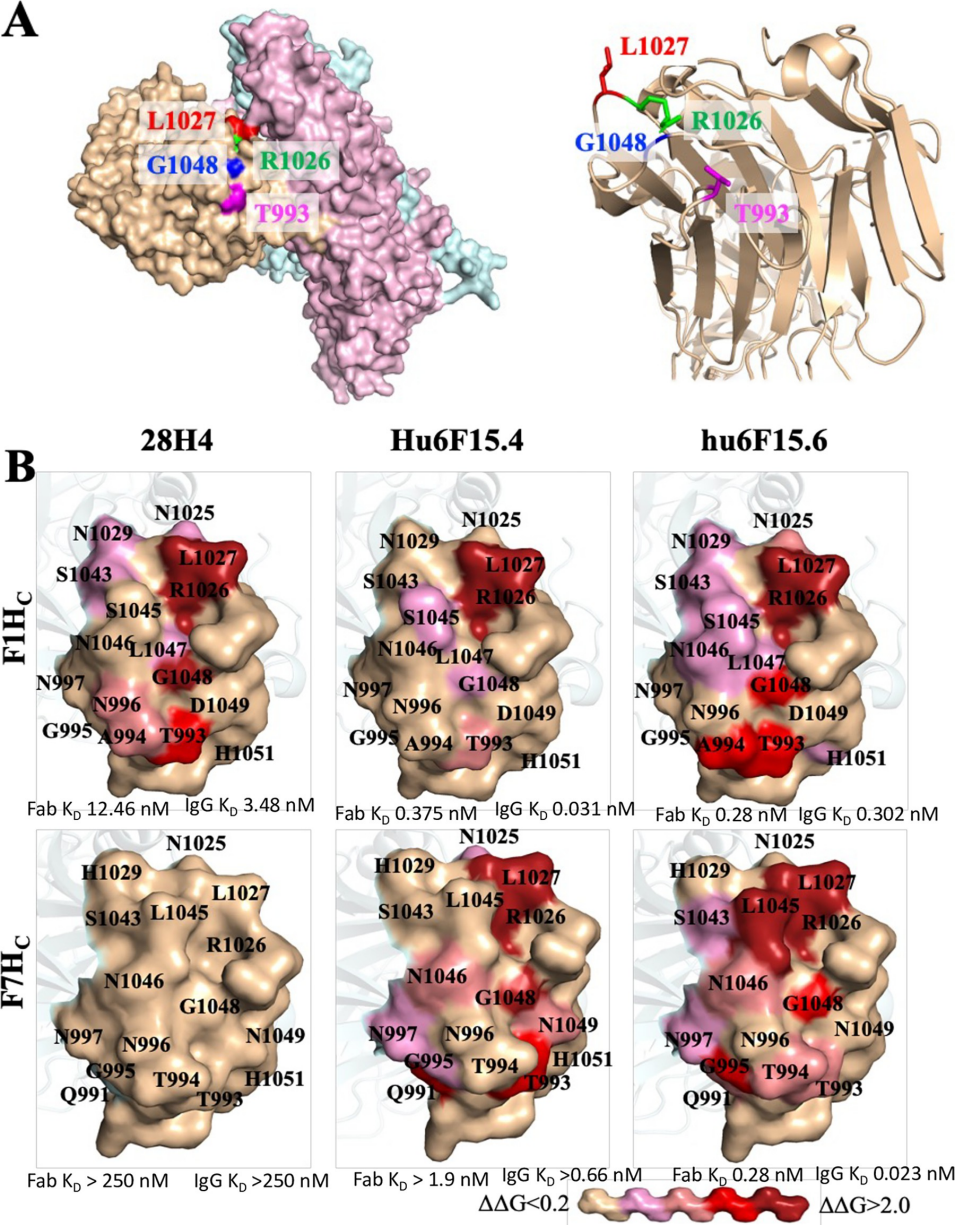
The epitopes of 28H4, hu6F15.4 and hu6F15.6 on BoNT/F1 and BoNT/F7. **A. The key amino acid residues in the epitope of 28H4, hu6F15.4 and hu6F15.6.** In the left panel, four key residues for the binding of 28H4 to BoNT/F1 (T993, R1026, L1027 and G1048) are shown on a surface model of the BoNT/F1 holotoxin (Alphafold, AF-A7GBG3). The BoNT/F1 H_C_ is shown in beige, the catalytic domain (LC) in blue, and the translocation domain in pink. In the right panel, the side chains of the same four key residues are shown on a ribbon diagram of the crystal structure of BoNT/F1 H_C_ (pdb, 3fuq). The four key amino acid residues are located on three different loops **B. The fine epitope maps of mAbs 28H4.** The contribution of amino acid side chains in mAb binding was measured and shown by coloring the surface side chain projection on either the BoNT/F1H_C_ structure (pdb, 3fuq) or a model of the BoNT/F7H_C_ based on 3fuq. Surface side chains were colored based on the values of ΔΔG from beige (no impact on mAb binding, ΔΔG<0.2) to dark red, ΔΔG>2.0 according to the key shown at the bottom of the Figure.

To better understand the structural basis of increased BoNT/F subtype cross-reactivity, the fine epitopes of 28H4, hu6F15.4 and hu6F15.6 on both BoNT/F1H_C_ and F7H_C_ were mapped. Hu6F15.4 and hu6F15.6 were selected for epitope mapping as they represented the final outputs of molecular evolution, had the same parental mAb, but differed in their subtype binding properties and selection methodology (hu6F15.4 selected simultaneously on multiple subtypes using multicolor sorting vs hu6F15.6 selected only on the BoNT/F7 subtype). Epitopes were mapped by isolation of BoNT/F1 H_C_ mutants that had significantly reduced or no binding to hu6F15.4 using methods previously described and detailed in the methods section [27, 35]. Briefly, random mutations were introduced into the BoNT/F1 H_C_ gene by error-prone PCR, the mutant repertoire cloned into the vector pYD4 and displayed on the surface of EBY100 yeast. BoNT/F1 H_C_ mutations with loss of hu6F15.4 binding were enriched through three rounds of FACS sorting and sequencing. Amino acids, together with adjacent residues identified on structural models of the BoNT/F1 H_C_ or F7 H_C_ surface, were individually mutated to alanine, displayed on the yeast surface and the changes of Gibbs free energy (ΔΔG) calculated to evaluate the contribution of each amino acid in the epitope as previously described [27, 35] (S2 Fig). The location of energetically important amino acids in the epitopes were then visualized on models of the BoNT/F1 holotoxin, the BoNT/F1 H_C_ crystal structure and a model of the BoNT/F1 H_C_ structure (Fig 6).

For the three mAbs, the number of amino acid residues with Δ Δ G > 0.2 ranged from 5 to 13 amino acids. Of these, three amino acids were different between BoNT/F1 and BoNT/F7, while the remaining six subtypes (BoNT/F2 -F6) had at most two amino acids, which differed from BoNT/F1. The 28H4 epitope consisted of amino acids located on four different BoNT/F H_C_ loops with 10 amino acids (solvent-accessible surface area, 1044.6 Å^2^) making energetically important contacts (Fig 6). Four amino acids, R1026, L1027, G1048 and T993 provided the largest energetic contributions with ΔΔG of 1.82 - >2.13 kcal/mol. Since 28H4 did not have detectable binding to BoNT/F7 H_C_, amino acid contributions to binding could not be measured. Molecular evolution of mAb 28H4 resulted in relatively similar epitopes for mAb hu6F15.6 on both BoNT/F1 H_C_ and BoNT/F7 H_C_ with epitopes of 13 and 12 amino acids. R1026, L1027 and G1048 remained high energy contacts for binding of hu6F15.6 to both BoNT/F subtypes. Hu6F15.6 picks up an additional high energy contact (A994) for binding to BoNT/F1 and three new low energy contacts (L1044, N1046 and I105, ΔΔG of 0.22–0.38 kcal/mol). Residue 994 has less contribution to binding to BoNT/F7 where the amino acid is different, T994 vs A994. Finally, a new high energy contact is created, L1045, for binding to BoNT/F7 as well as four new moderate energy contacts, Q991, G995, I1044 and N1046, ΔΔG of 0.59–1.4 kcal/mol). L1045 is different in BoNT/F1 (S1045) and is not used in binding of either 28H4 or hu6F15.6 to BoNT/F1.

The epitope of hu6F15.4 on BoNT/F1, in contrast, is much smaller than the epitopes of 28H4 and hu6F15.6 with only two high energy contacts R1026 and L1027 and three additional moderate or low energy contacts (G1048, S1045 and T993). For binding to BoNT/F7, the epitope size increases significantly, with a total of 11 contacts (7 new contacts, the loss of one contact and an increase in the binding energetics of two contacts.

This epitope mapping provides some insights into the basis of the specificity and affinity of hu6F15.4 and hu6F15.6 compared to 28H4 and their immediate molecular evolution predecessor hu6F15.3. Additional energetic contacts of hu6F15.6 compared to 28H4 likely account for the 45-fold higher affinity of the Fab for BoNT/F1. It is not possible to make a similar comparison to 28H4 of energetically important residues for hu6F15.4 and hu6F15.6 binding to BoNT/F7 since we could not detect binding of 28H4 to BoNT/F7. Of note, however, the epitope for hu6F15.6 binding to BoNT/F7 looks comparable to that of its binding to BoNT/F1 and 28H4 binding to BoNT/F1. The addition of the single new high energy contact L1045 and multiple moderate energy contacts likely account for the comparable and high binding affinity of hu6F15.6 to BoNT/F1 and BoNT/F7 (0.28 nM for both for Fab binding). The lower affinity binding of hu6F15.4 to BoNT/F7 (1.9 nM for the Fab) is likely due to the absence of the L1045 high energy contact. The very high affinity of hu6F15.6 for BoNT/F7 comes at a cost with respect to broad specificity. BoNT subtypes F2, F3, F4, F5, and F6 have amino acids in two positions much more like BoNT/F1 than BoNT/F7 including all having S1045 not L1045, and A994 or S994, not T994. Thus hu6F15.6 cannot make these high and moderate energy contacts to these subtypes. This likely accounts for hu6F15.6 having a 2.4-fold to 18-fold lower affinity for IgG binding to BoNT/F2, F3, F4, F5, and F6 than hu6F15.4. Finally, the epitope mapping that shows far fewer energetically important amino acids does not well explain the basis of the higher affinity of hu6F15.4 compared to 28H4 nor the comparable affinity with hu6F15.6. One possible explanation is that hu6F15.4 makes more contacts with the BoNT/F main chain, interactions that would not be quantitated by alanine mapping.

## Discussion

High affinity is crucial for neutralizing mAbs to clear and neutralize botulinum neurotoxins (BoNT) because of their extreme potency [40]. It has been established that a combination of at least three mAbs with high affinity is required to neutralize BoNT with a potency that allows a clinically reasonable dose, while any single mAb even with extremely high affinity (K_D_ = 56 pM) does not achieve this potency [13, 16–18]. In addition, cross-specificity of mAbs is necessary since each BoNT serotype usually contains multiple subtypes. To achieve the minimum number of three antibodies in the BoNT antitoxin, binding of antibodies to all subtypes is required. Cross-specificity could be achieved either through immunization of animals with combinations of different antigens or through antibody engineering via mutagenesis and selection For example, antibodies to HPV 16, 18, 31, and 45 were raised in mice immunized with a minor capsid protein L2-RG1 from HPV16 and 18 [41], while chain shuffling and structure-based “hot spot” mutagenesis enabled a mAb to HIV to obtain broad neutralization [42]. Most recently, a trispecific mAb that targeted CD4+ cells and two HIV surface antigens was developed to neutralize various HIV strains, efficiently preventing HIV infection in primates [43]. Similarly, a mAb with high affinity to BoNT/A1 but with lower affinity to BoNT/A2 was engineered to have increased affinity against BoNT/A2 while maintaining high-affinity binding to BoNT/A1 [1].

The high level of sequence dissimilarity among the BoNT/F subtypes (up to 36% dissimilar) led to the varied binding affinity of mAbs to different subtypes and identification of only three mAbs binding seven BoNT/F subtypes [13]. To provide an additional lead mAb for clinical development, we humanized and broadened the subtype specificity of murine mAb 28H4. The parental mAb 28H4 had no detectable binding to BoNT/F subtypes F2, F5, and F7 at BoNT concentrations of 250 nM suggesting an affinity lower than 1–10 μM. In fact, it could be presumed that 28H4 did not bind these subtypes. Despite this, using serial rounds of mutagenesis and sorting on multiple BoNT subtypes, a humanized mAb hu6F15.4 was generated that bound BoNT/F1, F2, F3, F4, F5, F6, and F7 with K_D_ ranging from 5.8 to 660 pM. For subtypes F1, F3, F4, and F6, affinity increased from 100 to 229-fold for hu6F15.4 compared to the parental 28H4. For subtypes F2, F5, and F7, where the parental affinity was no better than 1 μM, affinity increased at least 1,500 to 17,500-fold. Improving subtype cross-reactivity also did not come at the expense of loss of specificity; none of the higher affinity mAbs showed significant binding to any of the other BoNT serotypes or to the proteins in human or hamster cell lysates (Fig 5).

We did not systematically evaluate different staining and sorting strategies except for the final round of affinity maturation where we compared a strategy staining only with BoNT/F7 to a strategy using multicolor staining where the yeast library was simultaneously stained with BoNT subtypes labeled with different fluorophores (multicolor FACS). Multicolor FACS led to a more homogeneous range of subtype affinities and thus better subtype cross-reactivity compared to staining with only a single subtype. While multicolor flow cytometry has been routinely used in immunological research and for disease diagnosis [44, 45], our current study is the first report to apply multicolor FACS to isolate cross-reactive mAbs from yeast-displayed scFv libraries stained with up to six different dye-conjugated antigens. Multicolor FACS turned out to be quite efficient for isolation of cross-specificity BoNT/F mAb and could be more generally applied for generation or modification of multi-specific therapeutic mAbs.

In conclusion, multicolor FACS technology is a powerful method that could facilitate the isolation of mAb variants with the desired affinity for multiple antigens and could be generally used to generate multi-specific antibodies, to humanize non-human antibodies, and to improve antibody affinity to different homologous antigens.

## Supporting information

S1 Fig Binding specificity of parental mAb 28H4. **A. Dot plots of the binding of mAb 28H4 to yeast displayed BoNT/F domains.** Yeast displayed BoNT/F1 H_C_, H_N_ or LC were stained with IgG 28H4 and binding detected with anti-human PE. The display level of each domain on the surface of yeast was quantitated with the mAb SV5-FITC that bound a C-terminal SV5 tag on the yeast displayed domain. 28H4 binds the BoNT/F H_C_. **B. Dot plots of the binding of mAb 28H4 to yeast displayed BoNT/F domains of different BoNT F subtypes.** Yeast displayed BoNT/F1-7 H_C_ were stained with IgG 28H4 and binding detected with anti-human PE. The display level of each domain on the surface of yeast was quantitated with the mAb SV5-FITC that bound a C-terminal SV5 tag on the yeast displayed domain.
(PDF)

S2 Fig The fine epitopes of mAbs 28H4, Hu6F15.4 and hu6F15.6 on BoNT/F H_C_. The energetic contribution of amino acid residues in the epitopes of 28H4, hu6F15.4 and hu6F15.6 Fab binding was determined by measuring the change in Gibbs free energy (ΔΔG) occurring when the wild-type amino acid in the yeast displayed BoNT/F1 or BoNT/F7 H_C_ was changed to alanine (where alanine was the wild-type amino acid the residue was changed to glycine). The wild-type amino acid and its numbering is indicated in the amino acid column and the ΔΔG value for each amino acid is indicated with that Table cell colored according to the magnitude of the change in ΔΔG as shown in the Figure legend. Asterisk indicates amino acids that differ between BoNT/ F1 and F7. For BoNT/F subtypes F2, F3, F4, F5 and F6, the amino acid at each position is also indicated with amino acids differing between BoNT/F1 and the indicated subtype shown in red font.
(PDF)

S1 Data Supporting data for Table 1, K_D_ calculations of scFv. (XLSX)

S2 Data Supporting data for Tm calculations, DSF raw data and plots. (XLSX)

S3 Data Supporting data for Table 2, KinExA plots and K_D_ calculations of IgG. (ZIP)

## Abbreviations

BoNT: Botulinum neurotoxin
BoNT/F: Botulinum neurotoxin serotype F
CDR: Complementarity-determining regions
CHO: Chinese hamster ovary cells
DSF: Differential scanning fluorimetry
E. coli: *Escherichia coli*
FACS: fluorescent activated cell sorting
HC: the heavy chain of BoNT
LC: botulinum neurotoxin light chain
H_C_: the C-terminal portion of the botulinum neurotoxin heavy chain, or the binding domain
H_N_: the N-terminal portion of the botulinum neurotoxin heavy chain or Translocation domain
IMAC: immobilized metal affinity chromatography
IgG: immunoglobulin G
IPTG: isopropyl-β-D-thiogalactopyranoside
K_D_: dissociation equilibrium constant
KinExA: kinetic exclusion assay
k_on_: association rate constant
k_off_: dissociation rate constant
mAb: monoclonal antibody
MFI: mean fluorescence intensity
RMSD: root-mean-square deviation
scFv: single-chain variable fragment
SD-CAA: selective growth dextrose casamino acids media
SG-CAA: selective growth galactose casamino acids media
SDS-PAGE: sodium dodecyl sulfate polyacrylamide gel electrophoresis
SDM: structural distance measure
SDR: specificity determining residues
USAMRIID: United States Army Medical Research Institute of Infectious Disease
V_H_: Heavy chain variable region
Vk: Kappa variable, light chain
YPD: the rich media for yeast culture with yeast extract, peptone and dextrose

## References

[1] Garcia-Rodriguez C, Geren I, Lou J, Conrad F, Forsyth C, Wen W, et al. Neutralizing human monoclonal antibodies binding multiple serotypes of botulinum neurotoxin. Protein Engineering, Design & Selection. 2011;24(3):321–31. doi: 10.1093/protein/gzq111 21149386 PMC3038462

[2] Meyer RF, Miller L, Bennett RW, MacMillan JD. Development of a monoclonal antibody capable of interacting with five serotypes of Staphylococcus aureus enterotoxin. Applied Enviro Micro. 1984;47(2):283–7. doi: 10.1128/aem.47.2.283-287.1984 6712209 PMC239660

[3] Flyak AI, Shen X, Murin CD, Turner HL, David JA, Fusco ML, et al. Cross-Reactive and Potent Neutralizing Antibody Responses in Human Survivors of Natural Ebolavirus Infection. Cell. 2016;164(3):392–405. doi: 10.1016/j.cell.2015.12.022 26806128 PMC4733404

[4] Rockx B, Corti D, Donaldson E, Sheahan T, Stadler K, Lanzavecchia A, et al. Structural Basis for Potent Cross-Neutralizing Human Monoclonal Antibody Protection against Lethal Human and Zoonotic Severe Acute Respiratory Syndrome Coronavirus Challenge. J Virol. 2008;82(7):3220–35. doi: 10.1128/JVI.02377-07 18199635 PMC2268459

[5] Simmons CP, Bernasconi NL, Suguitan AL Jr, Mills K, Ward JM, Chau NVV, et al. Prophylactic and therapeutic efficacy of human monoclonal antibodies against H5N1 influenza. PLoS Med. 2007;4(5). doi: 10.1371/journal.pmed.0040178 17535101 PMC1880850

[6] Chen RE, Winkler ES, Case JB, Aziati ID, Bricker TL, Joshi A, et al. In vivo monoclonal antibody efficacy against SARS-CoV-2 variant strains. Nature. 2021;596(7870):103–8. doi: 10.1038/s41586-021-03720-y 34153975 PMC8349859

[7] Gill MD. Bacterial toxins: a table of lethal amounts. Microbiol Rev. 1982;46:86–94. doi: 10.1128/mr.46.1.86-94.1982 6806598 PMC373212

[8] Lacy DB, Stevens RC. Sequence homology and structural analysis of the Clostridial neurotxins. J Mol Biol. 1999;291:1091–104. 10518945 10.1006/jmbi.1999.2945

[9] Moller V, Scheibel I. Preliminary report on the isolation of an apparently new type of CI. botulinum. Acta pathologica et microbiologica Scandinavica. 1960;48:80. doi: 10.1111/j.1699-0463.1960.tb04741.x . 14423425

[10] Hill KK, Smith TJ. Genetic diversity within Clostridium botulinum serotypes, botulinum neurotoxin gene clusters and toxin subtypes. Current topics in microbiology and immunology. 2013;364:1–20. doi: 10.1007/978-3-642-33570-9_1 . 23239346

[11] Hill KK, Smith TJ, Helma CH, Ticknor LO, Foley BT, Svensson RT, et al. Genetic diversity among Botulinum Neurotoxin-producing clostridial strains. J Bacteriol. 2007;189(3):818–32. Epub 2006/11/23. JB.01180-06 [pii] doi: 10.1128/JB.01180-06 ; PubMed Central PMCID: PMC1797315. 17114256 PMC1797315

[12] Raphael BH, Choudoir MJ, Lúquez C, Fernández R, Maslanka SE. Sequence diversity of genes encoding botulinum neurotoxin type F. Applied Environ Micro. 2010;76(14):4805–12. doi: 10.1128/AEM.03109-09 20511432 PMC2901728

[13] Fan Y, Garcia-Rodriguez C, Lou J, Wen W, Conrad F, Zhai W, et al. A three monoclonal antibody combination potently neutralizes multiple botulinum neurotoxin serotype F subtypes. PLoS One. 2017;12(3):e0174187. doi: 10.1371/journal.pone.0174187 28323873 PMC5360321

[14] Smith TJ, Lou J, Geren IN, Forsyth CM, Tsai R, Laporte SL, et al. Sequence variation within botulinum neurotoxin serotypes impacts antibody binding and neutralization. Infect Immun. 2005;73(9):5450–7. doi: 10.1128/IAI.73.9.5450-5457.2005 ; PubMed Central PMCID: PMC1231122. 16113261 PMC1231122

[15] Cangene Corp., BAT® [Botulism Antitoxin Heptavalent (A, B, C, D, E, F, G)—(Equine)] Sterile Solution for Injection. Available online at: https://www.fda.gov/vaccines-blood-biologics/approved-blood-products/bat-botulism-antitoxin-heptavalent-b-c-d-e-f-g-equine 2013 [accessed on 27 February 2022].

[16] Garcia-Rodriguez C, Razai A, Geren IN, Lou J, Conrad F, Wen W-H, et al. A three monoclonal antibody combination potently neutralizes multiple botulinum neurotoxin serotype E subtypes. Toxins. 2018;10(3):105. doi: 10.3390/toxins10030105 29494481 PMC5869393

[17] Nowakowski A, Wang C, Powers DB, Amersdorfer P, Smith TJ, Montgomery VA, et al. Potent neutralization of botulinum neurotoxin by recombinant oligoclonal antibody. PNAS. 2002;99(17):11346–50. doi: 10.1073/pnas.172229899 12177434 PMC123259

[18] Garcia-Rodriguez C, Yan S, Geren IN, Knopp KA, Dong J, Sun Z, et al. A Four-Monoclonal Antibody Combination Potently Neutralizes Multiple Botulinum Neurotoxin Serotypes C and D. Toxins. 2021;13(9):641. doi: 10.3390/toxins13090641 34564645 PMC8472335

[19] Nayak SU, Griffiss JM, McKenzie R, Fuchs EJ, Jurao RA, An AT, et al. Safety and pharmacokinetics of XOMA 3AB, a novel mixture of three monoclonal antibodies against botulinum toxin A. Antimicrob Agents Chemother. 2014;58(9):5047–53. doi: 10.1128/AAC.02830-14 PubMed Central PMCID: PMC4135817. 24913160 PMC4135817

[20] Snow DM, Riling K, Kimbler A, Espinoza Y, Wong D, Pham K, et al. Safety and pharmacokinetics of a four monoclonal antibody combination against botulinum C and D neurotoxins. Antimicrob Agents Chemother. 2019;63(12). doi: 10.1128/AAC.01270-19 31591130 PMC6879217

[21] Guptill J, Raja S, Juel V, Walter E, Cohen-Wolkowiez M, Hill H, et al. Safety, Tolerability, and Pharmacokinetics of NTM-1632, a Novel Mixture of Three Monoclonal Antibodies against Botulinum Toxin B. Antimicrob Agents Chemother. 2021;65(7):e02329–20. 10.1128/AAC.02329-20 PMC8218613 33875433

[22] Raja SM, Guptill JT, Juel VC, Walter EB, Cohen-Wolkowiez M, Hill H, et al. First-in-Human Clinical Trial to Assess the Safety, Tolerability and Pharmacokinetics of Single Doses of NTM-1633, a Novel Mixture of Monoclonal Antibodies against Botulinum Toxin E. Antimicrob Agents Chemother. 2022;66(4):e0173221. Epub 20220321. doi: 10.1128/aac.01732-21 ; PubMed Central PMCID: PMC9017376. 35311524 PMC9017376

[23] Meng Q, Garcia-Rodriguez C, Manzanarez G, Silberg MA, Conrad F, Bettencourt J, et al. Engineered domain-based assays to identify individual antibodies in oligoclonal combinations targeting the same protein. Analytical biochemistry. 2012;430(2):141–50. doi: 10.1016/j.ab.2012.08.005 ; PubMed Central PMCID: PMC4209713. 22922799 PMC4209713

[24] Meng Q, Li M, Silberg MA, Conrad F, Bettencourt J, To R, et al. Domain-based assays of individual antibody concentrations in an oligoclonal combination targeting a single protein. Analytical biochemistry. 2012;421(2):351–61. doi: 10.1016/j.ab.2011.09.030 ; PubMed Central PMCID: PMC4209596. 22037290 PMC4209596

[25] Sikorra S, Skiba M, Dorner MB, Weisemann J, Weil M, Valdezate S, et al. Botulinum Neurotoxin F Subtypes Cleaving the VAMP-2 Q58–K59 Peptide Bond Exhibit Unique Catalytic Properties and Substrate Specificities. Toxins. 2018;10(8):311. doi: 10.3390/toxins10080311 30071628 PMC6116196

[26] Peck MW, Smith TJ, Anniballi F, Austin JW, Bano L, Bradshaw M, et al. Historical Perspectives and Guidelines for Botulinum Neurotoxin Subtype Nomenclature. Toxins. 2017;9(1). doi: 10.3390/toxins9010038 28106761 PMC5308270

[27] Fan Y, Dong J, Lou J, Wen W, Conrad F, Geren I, et al. Monoclonal Antibodies that Inhibit the Proteolytic Activity of Botulinum Neurotoxin Serotype/B. Toxins. 2015;7(9):3405–23. doi: 10.3390/toxins7093405 26343720 PMC4591640

[28] Boder ET, Wittrup KD. Yeast surface display for screening combinatorial polypeptide libraries. Nature Biotech. 1997;15(6):553–7. doi: 10.1038/nbt0697-553 9181578

[29] Brochet X, Lefranc M-P, Giudicelli V. IMGT/V-QUEST: the highly customized and integrated system for IG and TR standardized VJ and VDJ sequence analysis. Nucleic Acids Res. 2008;36(suppl_2):W503–W8. 18503082 10.1093/nar/gkn316 PMC2447746

[30] Foote J, Winter G. Antibody framework residues affecting the conformation of the hypervariable loops. J Mol Biol. 1992;224(2):487–99. doi: 10.1016/0022-2836(92)91010-m 1560463

[31] Chothia C, Novotný J, Bruccoleri R, Karplus M. Domain association in immunoglobulin molecules: the packing of variable domains. J Mol Biol. 1985;186(3):651–63. 4093982 10.1016/0022-2836(85)90137-8

[32] Chothia C, Lesk AM. Canonical structures for the hypervariable regions of immunoglobulins. J Mol Biol. 1987;196(4):901–17. doi: 10.1016/0022-2836(87)90412-8 3681981

[33] Niesen FH, Berglund H, Vedadi M. The use of differential scanning fluorimetry to detect ligand interactions that promote protein stability. Nature Protocols. 2007;2(9):2212–21. doi: 10.1038/nprot.2007.321 17853878

[34] Xu Y, Roach W, Sun T, Jain T, Prinz B, Yu T-Y, et al. Addressing polyspecificity of antibodies selected from an in vitro yeast presentation system: a FACS-based, high-throughput selection and analytical tool. Protein Engineering, Design & Selection. 2013;26(10):663–70. 10.1093/protein/gzt047 24046438

[35] Fan Y, Geren IN, Dong J, Lou J, Wen W, Conrad F, et al. Monoclonal antibodies targeting the alpha-exosite of botulinum neurotoxin serotype/A inhibit catalytic activity. PLoS One. 2015;10(8):e0135306. doi: 10.1371/journal.pone.0135306 26275214 PMC4537209

[36] Waterhouse A., Bertoni M., Bienert S., Studer G., Tauriello G., Gumienny R., et al. SWISS-MODEL: homology modelling of protein structures and complexes. Nucleic Acids Res. 46, W296–W303 (2018). doi: 10.1093/nar/gky427 29788355 PMC6030848

[37] Johnson MS, Sutcliffe MJ, Blundell TL. Molecular anatomy: phyletic relationships derived from three-dimensional structures of proteins. J Mol Evol. 1990;30(1):43–59. doi: 10.1007/BF02102452 2107323

[38] Krissinel E, Henrick K. Secondary-structure matching (SSM), a new tool for fast protein structure alignment in three dimensions. Acta Crystallogr D Biol Crystallogr. 2004;60(Pt 12 Pt 1):2256–68. Epub 2004/12/02. doi: 10.1107/S0907444904026460 . 15572779

[39] Pettersen EF, Goddard TD, Huang CC, Couch GS, Greenblatt DM, Meng EC, et al. UCSF Chimera—a visualization system for exploratory research and analysis. J Comput Chem. 2004;25(13):1605–12. Epub 2004/07/21. doi: 10.1002/jcc.20084 . 15264254

[40] Arnon SS, Schechter R, Inglesby TV, Henderson DA, Bartlett JG, Ascher MS, et al. Botulinum toxin as a biological weapon: medical and public health management. JAMA: the journal of the American Medical Association. 2001;285(8):1059–70. Epub 2001/03/07. doi: 10.1001/jama.285.8.1059 . 11209178

[41] Khiavi FM, Arashkia A, Nasimi M, Mahdavi M, Golkar M, Roohvand F, et al. Immunization of mice by a multimeric L2-based linear epitope (17–36) from HPV type 16/18 induced cross reactive neutralizing antibodies. Res Pharm Sci. 2017;12(4):265. doi: 10.4103/1735-5362.212043 28855937 PMC5566000

[42] Sui J, Aird DR, Tamin A, Murakami A, Yan M, Yammanuru A, et al. Broadening of neutralization activity to directly block a dominant antibody-driven SARS-coronavirus evolution pathway. PLoS Pathog. 2008;4(11):e1000197. doi: 10.1371/journal.ppat.1000197 18989460 PMC2572002

[43] Xu L, Pegu A, Rao E, Doria-Rose N, Beninga J, McKee K, et al. Trispecific broadly neutralizing HIV antibodies mediate potent SHIV protection in macaques. Science. 2017;358(6359):85–90. doi: 10.1126/science.aan8630 28931639 PMC5978417

[44] Perfetto SP, Chattopadhyay PK, Roederer M. Seventeen-colour flow cytometry: unravelling the immune system. Nature Rev Immunol. 2004;4(8):648–55. doi: 10.1038/nri1416 15286731

[45] Poulsen TR, Meijer P-J, Jensen A, Nielsen LS, Andersen PS. Kinetic, affinity, and diversity limits of human polyclonal antibody responses against tetanus toxoid. J Immunol. 2007;179(6):3841–50. doi: 10.4049/jimmunol.179.6.3841 17785821

