# Supplementary material for: Multicolor fluorescence activated cell sorting to generate humanized monoclonal antibody binding seven subtypes of BoNT/F
Source: PLoS One. 2022 Sep 1;17(9):e0273512. doi: 10.1371/journal.pone.0273512 (PMC9436041; doi:10.1371/journal.pone.0273512)
Supplement: S1 Fig — A. Dot plots of the binding of mAb 28H4 to yeast displayed BoNT/F domains. Yeast displayed BoNT/F1 HC, HN or LC were stained with IgG 28H4 and binding detected with anti-human PE. The display level of each domain on the surface of yeast was quantitated with the mAb SV5-FITC that bound a C-terminal SV5 tag on the yeast displayed domain. 28H4 binds the BoNT/F HC. B. Dot plots of the binding of mAb 28H4 to yeast displayed BoNT/F domains of different BoNT F subtypes. Yeast displayed BoNT/F1-7 HC were stained with IgG 28H4 and binding detected with anti-human PE. The display level of each domain on the surface of yeast was quantitated with the mAb SV5-FITC that bound a C-terminal SV5 tag on the yeast displayed domain. (PDF) [file pone.0273512.s001.pdf]

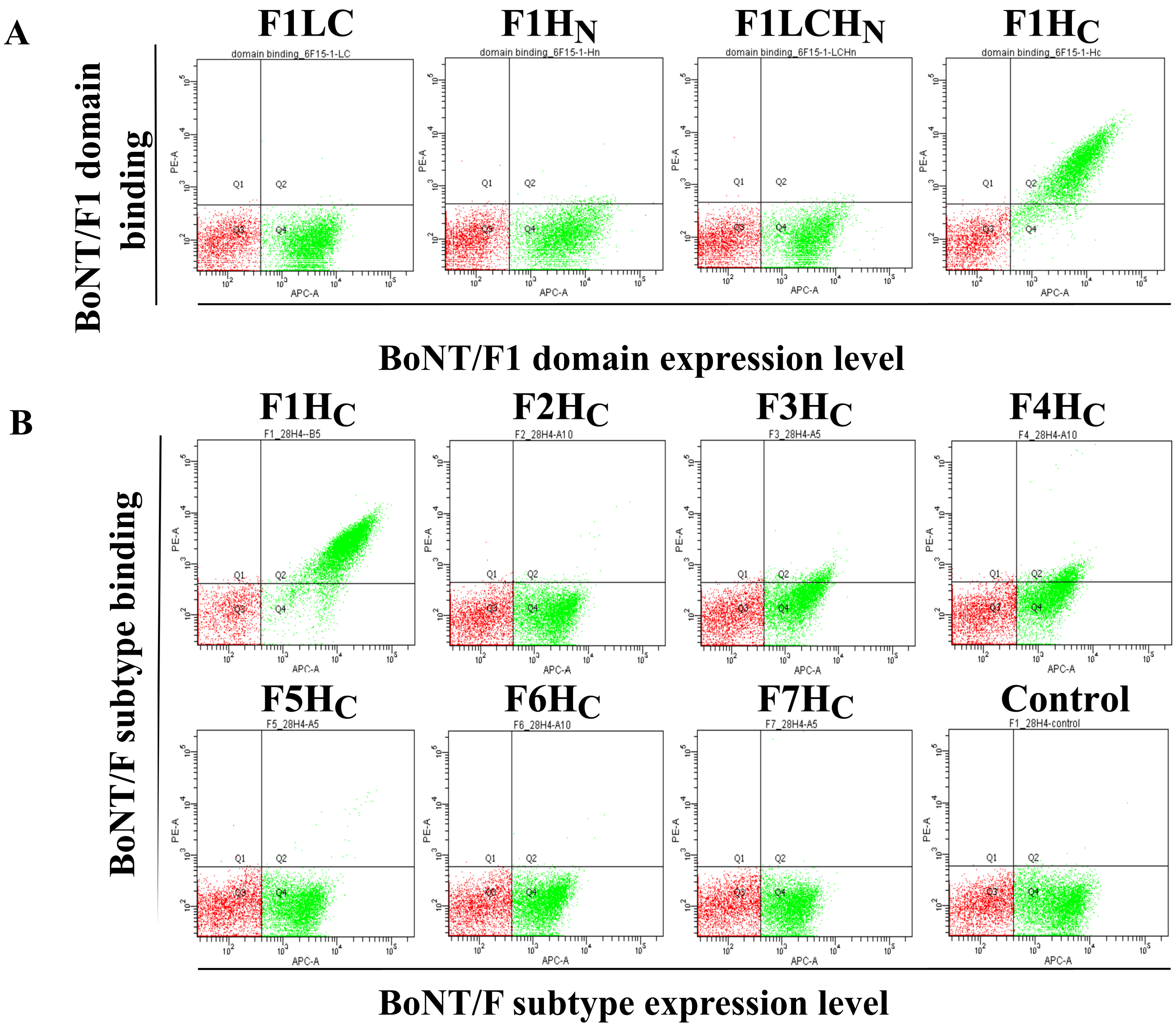
A
F1LC
F1HN
F1LCHN
F1HC
domain binding_6F15-1-LC
domain binding_6F15-1-Hn
domain binding_6F15-1-LCHn
domain binding_6F15-1-Hc
BoNT/F1 domain binding
BoNT/F1 domain expression level
B
F1HC
F2HC
F3HC
F4HC
F5HC
F6HC
F7HC
Control
F1_28H4--B5
F2_28H4-A10
F3_28H4-A5
F4_28H4-A10
F5_28H4-A5
F6_28H4-A10
F7_28H4-A5
F1_28H4-control
BoNT/F subtype binding
BoNT/F subtype expression level
PE-A
APC-A
Q1
Q2
Q3
Q4
