# Supplementary material for: Multicolor fluorescence activated cell sorting to generate humanized monoclonal antibody binding seven subtypes of BoNT/F
Source: PLoS One. 2022 Sep 1;17(9):e0273512. doi: 10.1371/journal.pone.0273512 (PMC9436041; doi:10.1371/journal.pone.0273512)
Supplement: S2 Fig — The energetic contribution of amino acid residues in the epitopes of 28H4, hu6F15.4 and hu6F15.6 Fab binding was determined by measuring the change in Gibbs free energy (ΔΔG) occurring when the wild-type amino acid in the yeast displayed BoNT/F1 or BoNT/F7 HC was changed to alanine (where alanine was the wild-type amino acid the residue was changed to glycine). The wild-type amino acid and its numbering is indicated in the amino acid column and the ΔΔG value for each amino acid is indicated with that Table cell colored according to the magnitude of the change in ΔΔG as shown in the Figure legend. Asterisk indicates amino acids that differ between BoNT/ F1 and F7. For BoNT/F subtypes F2, F3, F4, F5 and F6, the amino acid at each position is also indicated with amino acids differing between BoNT/F1 and the indicated subtype shown in red font. (PDF) [file pone.0273512.s002.pdf]

BoNT/F1H_C				BoNT/F7H_C			BoNT/F2	BoNT/F3	BoNT/F4	BoNT/F5	BoNT/F6
Amino acids	28H4	hu6F15.4	hu6F15.6	Amino acids	hu6F15.4	hu6F15.6	Amino acids	Amino acids	Amino acids	Amino acids	Amino acids
	ΔΔG	ΔΔG	ΔΔG		ΔΔG	ΔΔG					
1026R	>2.13	3.70	3.96	R	>2.0	2.59	R	R	R	R	R
1027L	>2.13	3.79	3.96	L	>2.0	>2.59	L	L	L	L	L
1048G	>2.13	0.50	1.82	G	>2.0	1.60	G	G	G	G	G
1045S	-0.12	0.24	0.07	L*	0.02	2.26	S	S	S	S	S
991Q	-0.03	-0.01	-0.04	Q	1.37	0.59	Q	Q	Q	Q	Q
993T	1.82	0.86	1.73	T	1.33	0.89	T	T	T	T	T
994A	0.73	-0.10	1.2	T*	0.03	0.64	S	S	**A**	S	S
996N	0.87	0.01	0.53	N	0.05	-0.11	N	N	N	N	N
1025N	0.31	0.12	0.62	N	0.39	0.14	N	N	N	N	N
995G	-0.21	-0.18	0.06	G	0.21	1.40	G	G	G	G	G
1044I	-0.22	-0.07	0.29	I	0.56	0.86	I	I	I	I	I
1047L	0.21	0.03	0.21	L	-0.50	-0.30	L	L	L	L	L
1046N	0.04	0.03	0.38	N	0.67	0.82	N	N	N	N	N
1051H	-0.10	-0.02	0.22	H	-0.84	-0.21	H	H	H	H	H
1029N	0.40	-0.02	0.20	H*	0.17	0.09	N	N	N	N	N
1043S	0.20	0.04	0.30	S	0.17	0.27	S	S	S	S	S
1048D	-0.18	0.07	0.10	N*	0.98	-0.12	D	D	D	D	D
1028G	0.18	0.01	0.07	G	-0.33	-0.02	G	G	G	G	G
997N	0.06	0.04	0.06	N	0.45	0.27	K	K	N	K	K

ΔΔG 0.2-0.5

ΔΔG 0.5-1.0

ΔΔG 1.0-2.0

ΔΔG >2.0

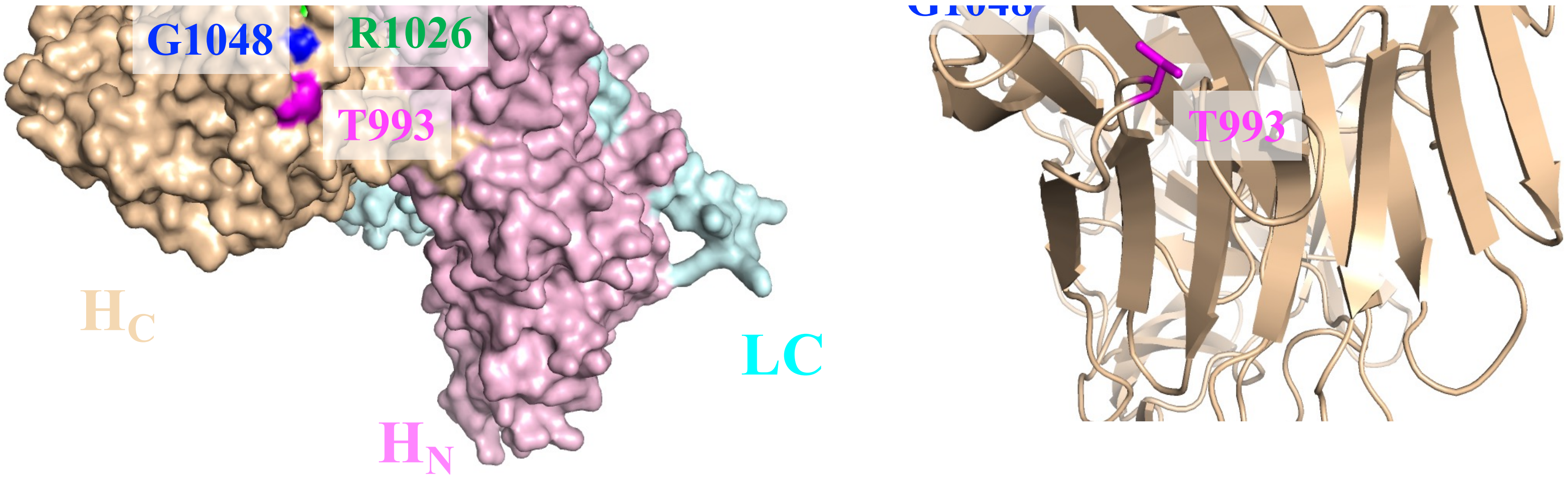
G1048
R1026
T993
H_C
H_N
LC
T993

B

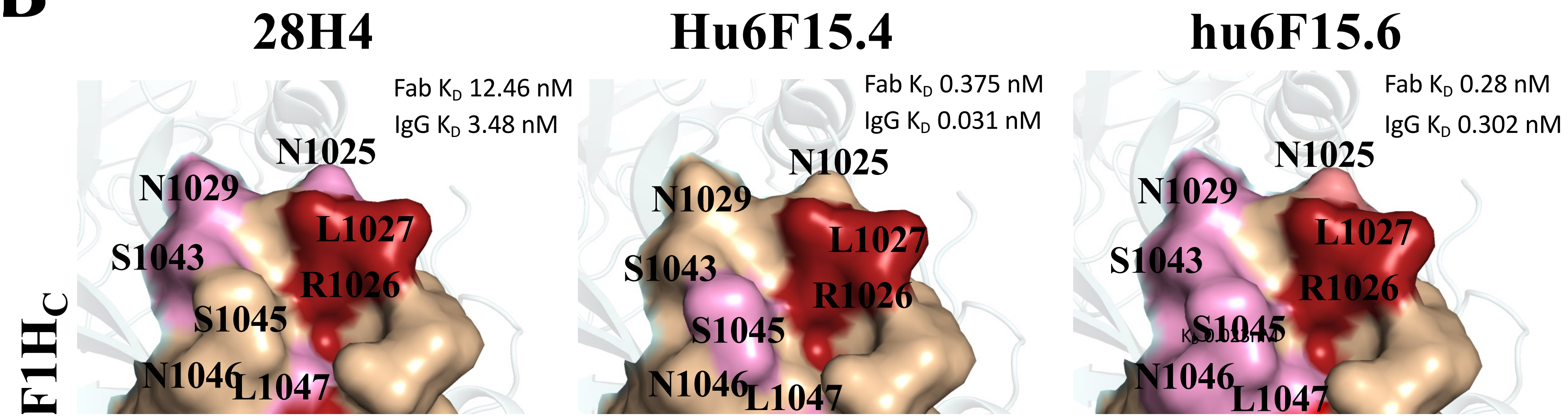
28H4
Hu6F15.4
hu6F15.6
$F1H_C$
Fab K_D 12.46 nM
IgG K_D 3.48 nM
Fab K_D 0.375 nM
IgG K_D 0.031 nM
Fab K_D 0.28 nM
IgG K_D 0.302 nM
N1025
N1029
L1027
S1043
R1026
S1045
N1046
L1047

A

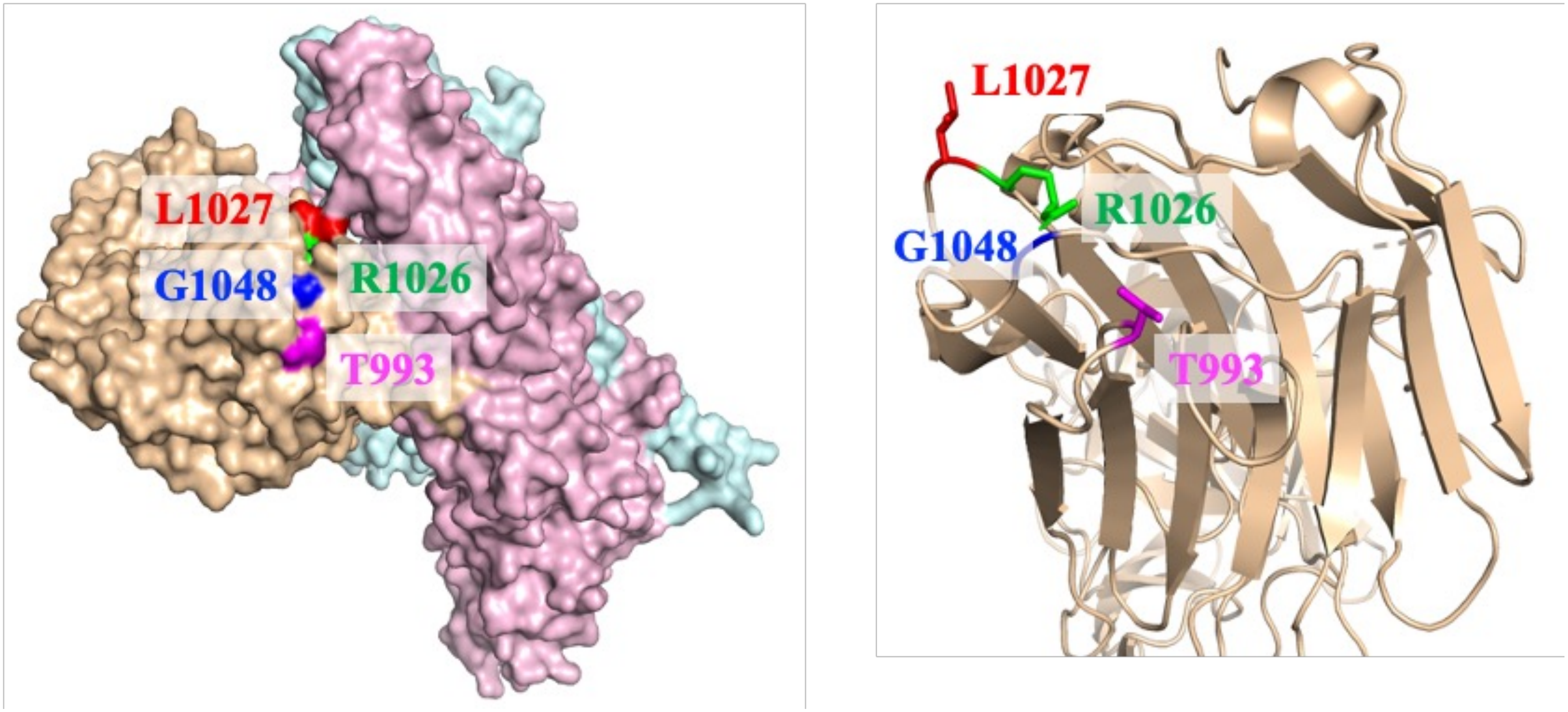

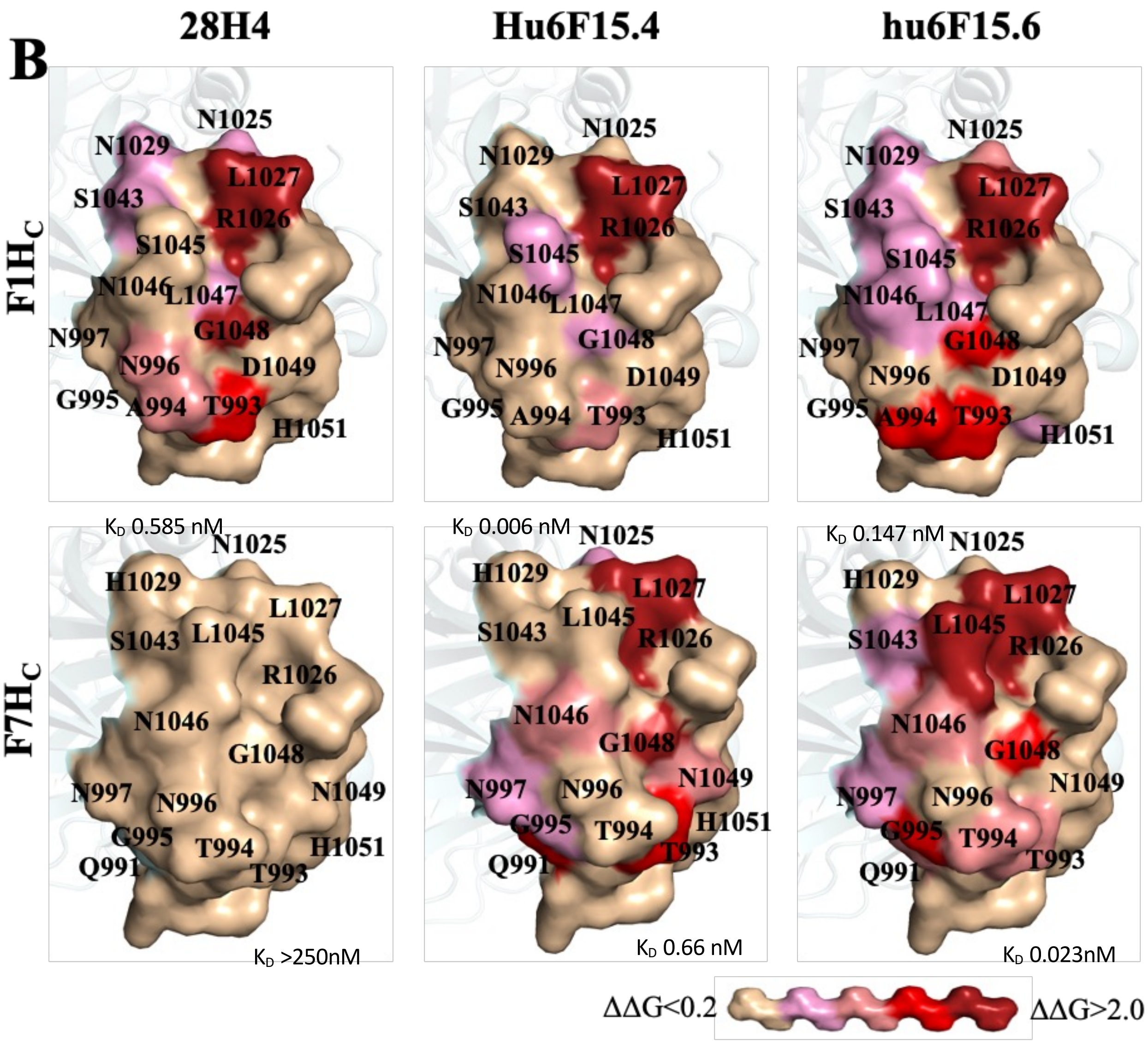
B
28H4
Hu6F15.4
hu6F15.6
F1H$_C$
F7H$_C$
N1025
N1029
L1027
S1043
R1026
S1045
N1046
L1047
G1048
N997
N996
D1049
G995
A994
T993
H1051
K_D 0.585 nM
K_D 0.006 nM
K_D 0.147 nM
H1029
L1045
N1049
T994
Q991
K_D >250nM
K_D 0.66 nM
K_D 0.023nM
ΔΔG<0.2
ΔΔG>2.0
