# Supplementary material for: Multicolor fluorescence activated cell sorting to generate humanized monoclonal antibody binding seven subtypes of BoNT/F
Source: PLoS One. 2022 Sep 1;17(9):e0273512. doi: 10.1371/journal.pone.0273512 (PMC9436041; doi:10.1371/journal.pone.0273512)

Experiment(x)

**Experiment Name:**	RF 6F15.3 vs F7 HC	**Start Time:**	Wed Sep 21 13:39:37 2016
**Experiment Type:**	Equilibrium	**End Time:**	Wed Sep 21 15:27:35 2016
**Constant Binding Partner (CBP)**		**Buffer:**	PBS/BSA
**Molecular Concentration:**	12.00nM	**Label:**	6F3-647
**Valency:**	1	**Label Conc:**	0
**Binding Site Concentration:**	12.00nM		

Comments(x)

beads: 6F15.3 9/21/16

sample volume: 500 ul

detection: 6F3-647

CBP: 12 nM BoNT F7 HC 12/2/10

titrant: 6F15.3 IgG 7/28/16

titration: 7 samples: 400 nM - 400 fM (1:10); + CBP only

samples:

1) NSB

2) 100% (CBP only)

3-9) titration of 6F15.3 IgG

Timing(x)

Bead Handling (Custom Beads)

Draw Source	Time (sec)	Volume (uL)	Rate (mL/min)	Stir
Backflush	20	0	0.0000	
Buffer	20	500	1.5000	✓
Particle Reservoir 1	20	333	1.0000	✓
Buffer	30	500	1.0000	
Waste	2	8	0.2500	
Buffer	20	0	0.0000	
Buffer	9	150	1.0000	

Sample Timing

Draw Source	Time (sec)	Volume (uL)	Rate (mL/min)	Time Stamp
Sample Set 1,309-316	120	500	0.2500	
Buffer	30	125	0.2500	
Rack 2: Tube 60	120	500	0.2500	
Buffer	30	125	0.2500	
Buffer	90	1500	1.0000	

Analysis(x)

Baseline / Endpoints:

5 to 10 (sec) from beginning

10 to 5 (sec) from end

Ignore	Binding Signal (V)	Concentration
✓	0.0420	0
	0.8974	0
	0.0528	400.00nM
	0.0939	40.00nM
	0.5080	4.00nM
	0.8226	400.00pM
	0.9031	40.00pM
	0.8726	4.00pM
	0.8917	400.00fM

**Kd:**	2.54nM
**Active CBP:**	4.14nM
**CBP %Activity:**	34.48
**Ratio:**	1.6316
**Sig 100%:**	0.89
**NSB:**	0.04
**%Error:**	1.27

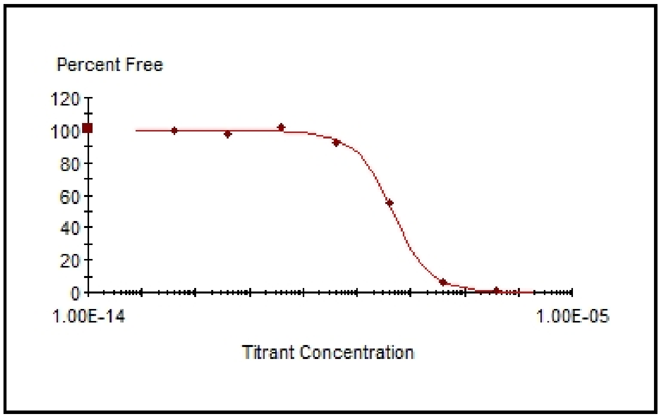

**Kd:**	2.54nM
**95% confidence interval**	
**Kd High:**	4.12nM
**Kd Low:**	1.15nM

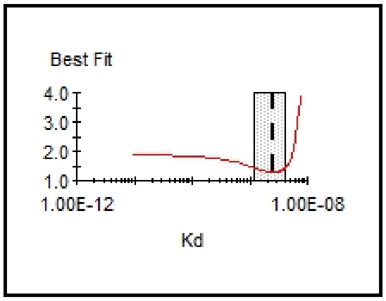

**Active CBP:**	4.14nM
**CBP %Activity:**	34.48
**95% confidence interval**	
**CBP High:**	7.24nM
**%Activity:**	60.35
**CBP Low:**	Less than 1.34nM
**%Activity:**	Less than 11.20

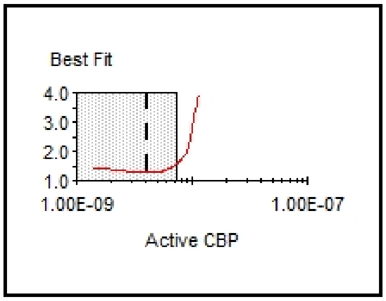

Data Traces(x)

Cycles: 1
Incubation delay (min): 0
Mix Time:

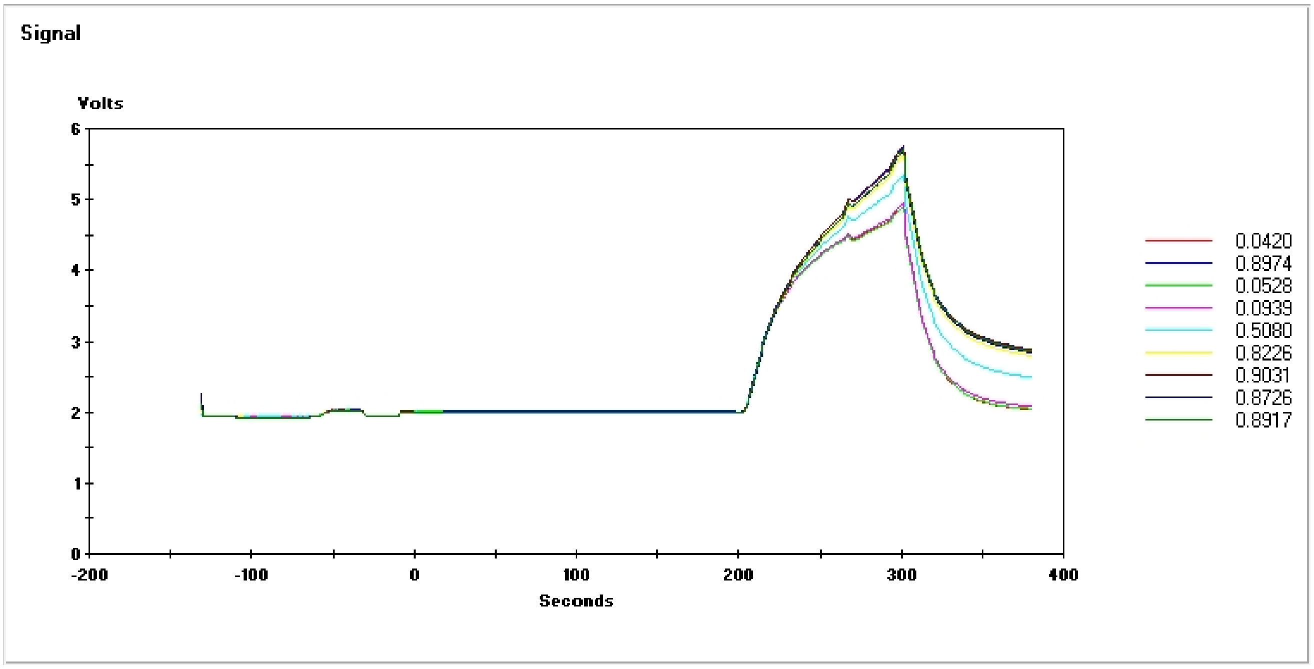

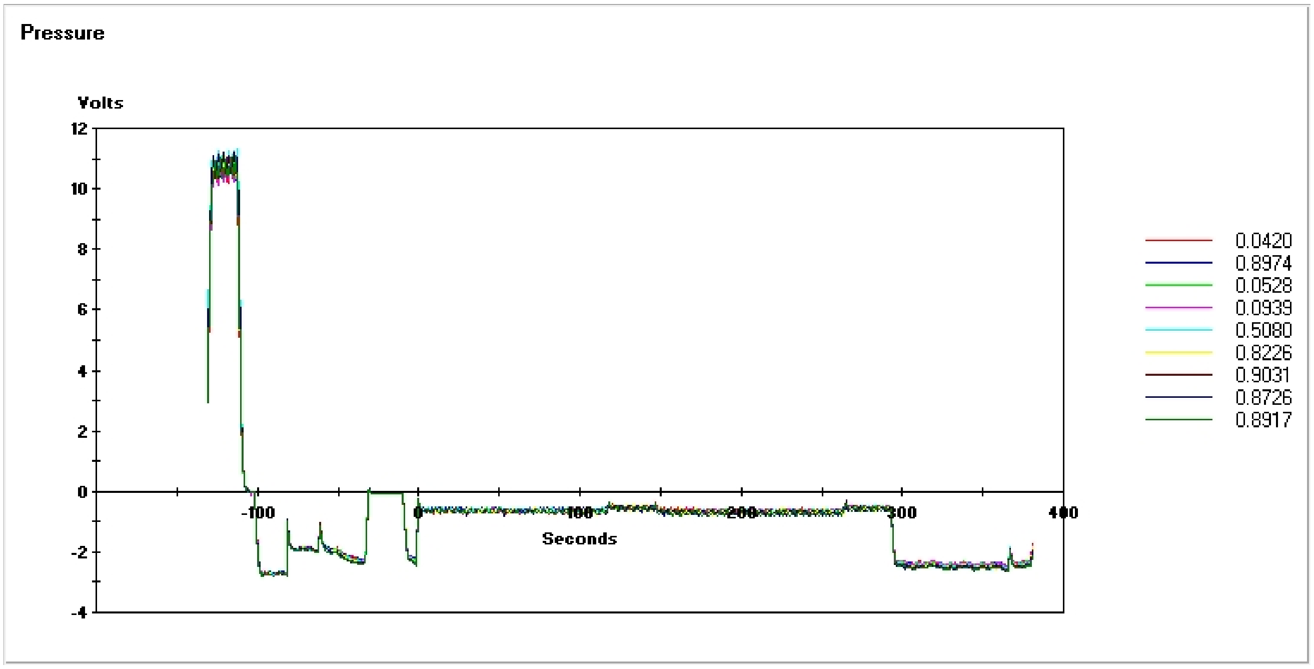

Supplement: S3 Data — (ZIP) [file pone.0273512.s005.zip › IgG KD measurements KinExA/RF 6F15.3 vs F7 HC.pdf]
