# Supplementary material for: Multicolor fluorescence activated cell sorting to generate humanized monoclonal antibody binding seven subtypes of BoNT/F
Source: PLoS One. 2022 Sep 1;17(9):e0273512. doi: 10.1371/journal.pone.0273512 (PMC9436041; doi:10.1371/journal.pone.0273512)

Experiment(x)

**Experiment Name:**	RF Hu6F15.4 vs F5 HNHC-MBP{3}	**Start Time:**	Wed Aug 30 10:25:00 2017
**Experiment Type:**	Equilibrium	**End Time:**	Wed Aug 30 14:59:37 2017
**Constant Binding Partner (CBP)**		**Buffer:**	PBS/BSA
**Molecular Concentration:**	100.00pM	**Label:**	6F5.4-647
**Valency:**	1	**Label Conc:**	0
**Binding Site Concentration:**	100.00pM		

Comments(x)

beads: Hu6F15.3 8/28/17

sample volume: 6 ml

detection: 6F5.4-647

CBP: 100 pM BoNT F5 HNHC-MBP 11/16/15

titrant: Hu6F15.4 IgG 2/1/17

titration: 7 samples: 40 nM - 40 fM (1:10)

samples:

1) NSB

2-8) titration

Timing(x)

Bead Handling (Custom Beads)

Draw Source	Time (sec)	Volume (uL)	Rate (mL/min)	Stir
Backflush	20	0	0.0000	
Buffer	20	500	1.5000	✓
Particle Reservoir 1	18	300	1.0000	✓
Buffer	30	500	1.0000	
Waste	2	8	0.2500	
Buffer	20	0	0.0000	
Buffer	9	150	1.0000	

Sample Timing

Draw Source	Time (sec)	Volume (uL)	Rate (mL/min)	Time Stamp
Sample Set 1,201-207	1440	6000	0.2500	
Buffer	30	125	0.2500	
Rack 2: Tube 60	120	500	0.2500	
Buffer	30	125	0.2500	
Buffer	90	1500	1.0000	

Analysis (x)

Baseline / Endpoints:
5 to 10 (sec) from beginning
10 to 5 (sec) from end

Ignore	Binding Signal (V)	Concentration
✓	0.0805	NSB
	0.1376	40.00nM
	0.1479	4.00nM
	0.2698	400.00pM
	0.4418	40.00pM
	0.4900	4.00pM
	0.4871	400.00fM
	0.4913	40.00fM

**Kd:**	229.84pM
**Active CBP:**	38.49pM
**CBP % Activity:**	38.49
**Ratio:**	0.1675
**Sig 100%:**	0.49
**NSB:**	0.13
**%Error:**	0.78

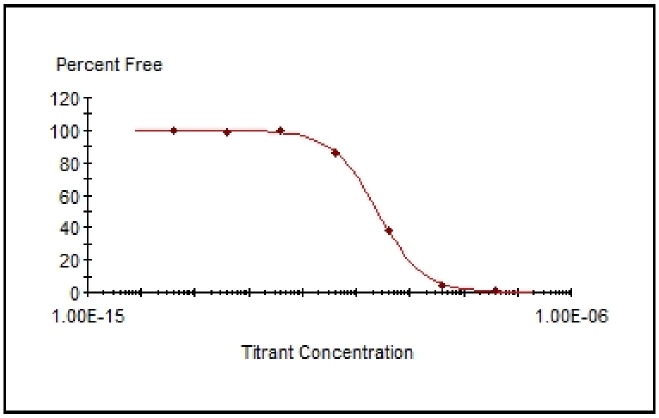

**Kd:**	229.84pM
**95% confidence interval**	
**Kd High:**	258.94pM
**Kd Low:**	198.65pM

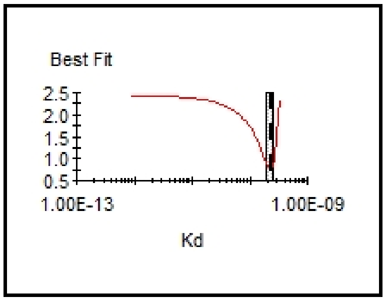

**Active CBP:**	38.49pM
**CBP %Activity:**	38.49
**95% confidence interval**	
**CBP High:**	131.25pM
**%Activity:**	131.25
**CBP Low:**	Less than 139.06fM
**%Activity:**	Less than 0.14

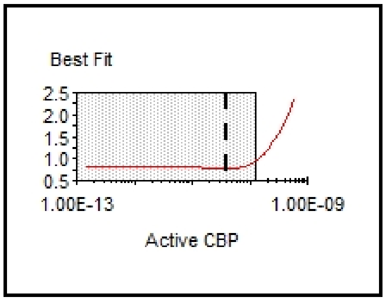

Data Traces(x)

Cycles: 1
Incubation delay (min): 0
Mix Time:

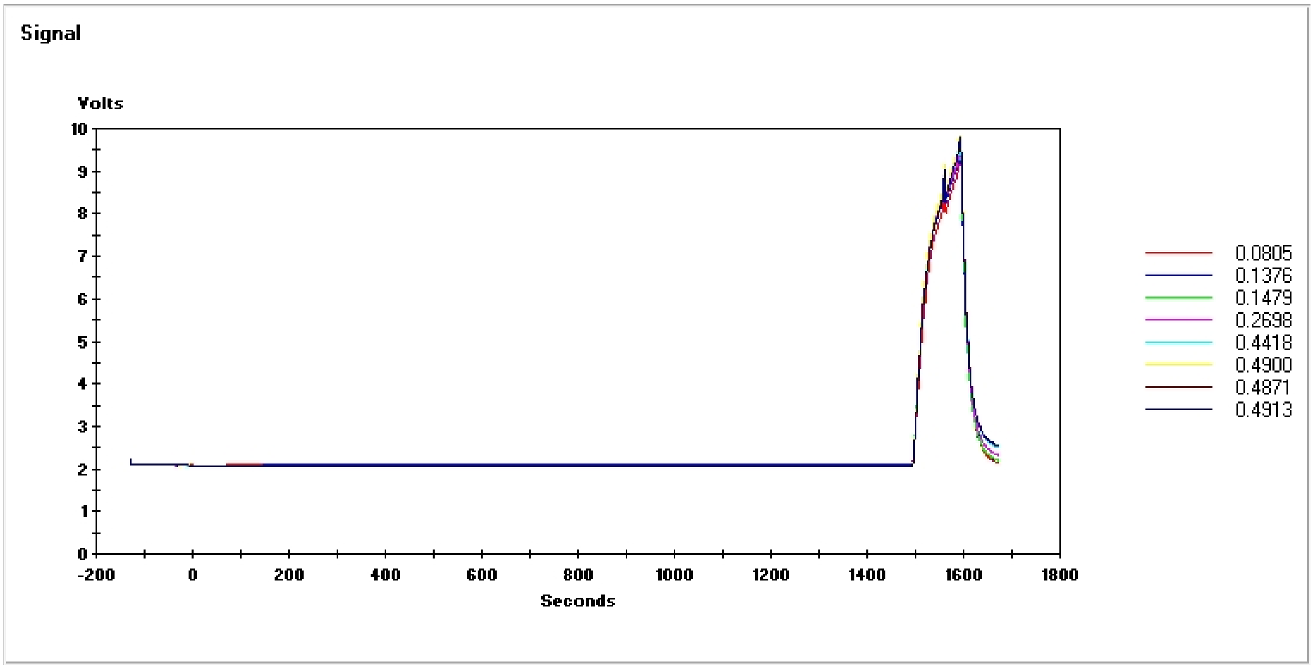

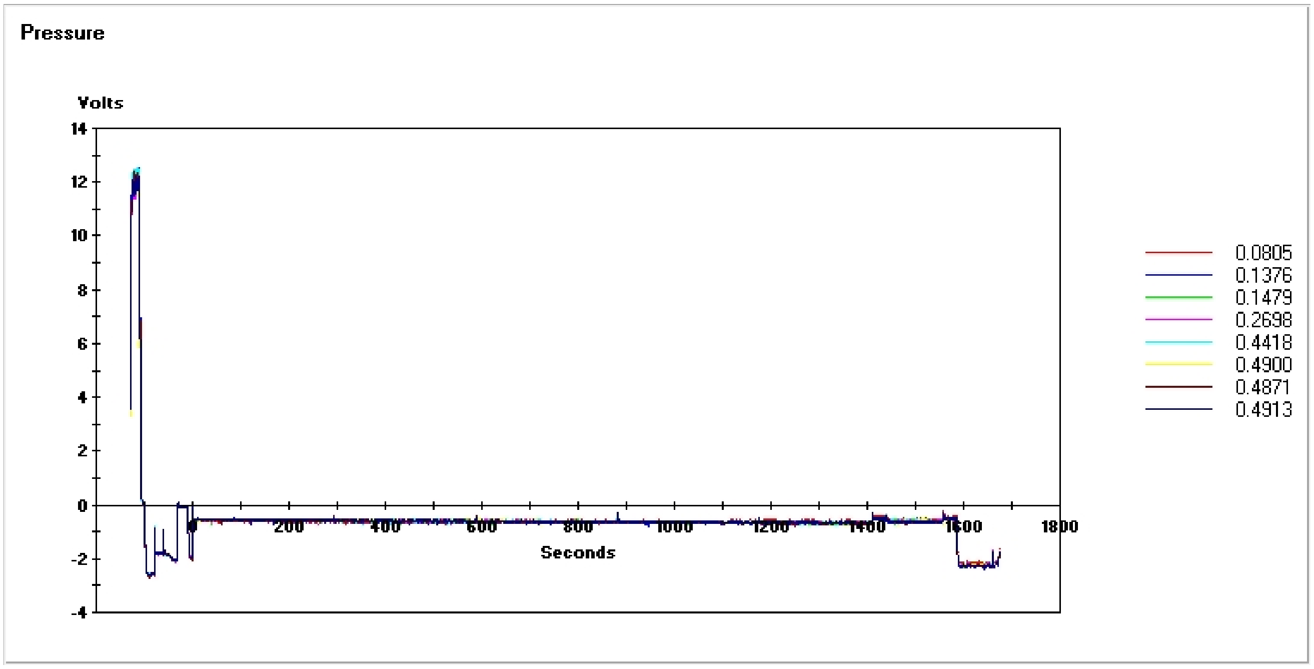

Supplement: S3 Data — (ZIP) [file pone.0273512.s005.zip › IgG KD measurements KinExA/RF Hu6F15.4 vs F5 HNHC-MBP{3}.pdf]
