# Supplementary material for: Multicolor fluorescence activated cell sorting to generate humanized monoclonal antibody binding seven subtypes of BoNT/F
Source: PLoS One. 2022 Sep 1;17(9):e0273512. doi: 10.1371/journal.pone.0273512 (PMC9436041; doi:10.1371/journal.pone.0273512)

Experiment(x)

**Experiment Name:**	RF 28H4 vs F4 HC-MBP	**Start Time:**	Tue Aug 15 16:21:38 2017
**Experiment Type:**	Equilibrium	**End Time:**	Tue Aug 15 17:57:32 2017
**Constant Binding Partner (CBP)**		**Buffer:**	PBS/BSA
**Molecular Concentration:**	800.00pM	**Label:**	6F8-647
**Valency:**	1	**Label Conc:**	0
**Binding Site Concentration:**	800.00pM		

Comments(x)

beads: Hu6F15.3 8/10/17

sample volume: 500 ul

detection: 6F8-647

CBP: 800 pM BoNT F4 HC-MBP 12/17/14 (diluted to 100 nM 2/28/17)

titrant: 28H4 IgG 11/3/16

titration: 7 samples: 100 nM - 100 fM (1:10)

samples:

1) NSB

2-8) titration

Timing(x)

Bead Handling (Custom Beads)

Draw Source	Time (sec)	Volume (uL)	Rate (mL/min)	Stir
Backflush	20	0	0.0000	
Buffer	20	500	1.5000	✓
Particle Reservoir 1	20	333	1.0000	✓
Buffer	30	500	1.0000	
Waste	2	8	0.2500	
Buffer	20	0	0.0000	
Buffer	9	150	1.0000	

Sample Timing

Draw Source	Time (sec)	Volume (uL)	Rate (mL/min)	Time Stamp
Sample Set 1,301-307	120	500	0.2500	
Buffer	30	125	0.2500	
Rack 2: Tube 60	120	500	0.2500	
Buffer	30	125	0.2500	
Buffer	90	1500	1.0000	

Analysis (x)

Baseline / Endpoints:
5 to 10 (sec) from beginning
10 to 5 (sec) from end

Ignore	Binding Signal (V)	Concentration
✓	0.2291	NSB
	0.2212	100.00nM
	0.3587	10.00nM
	0.7165	1.00nM
	0.8969	100.00pM
	0.9541	10.00pM
	0.9378	1.00pM
	0.9730	100.00fM

Kd: 2.21nM
Active CBP: 2.13pM
CBP %Activity: 0.27
Ratio: 0.0010
Sig 100%: 0.95
NSB: 0.21
%Error: 1.80

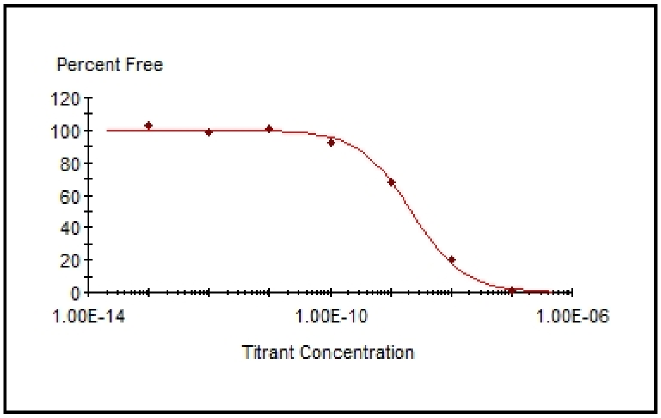

Kd: 2.21nM
95% confidence interval
Kd High: 2.57nM
Kd Low: 1.82nM

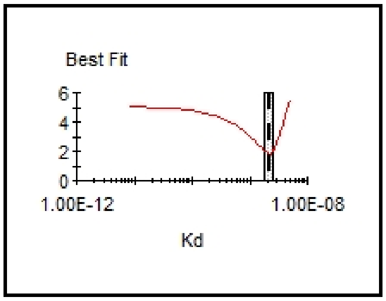

Active CBP: 2.13pM
CBP %Activity: 0.27
95% confidence interval
CBP High: Greater than 590.45pM
%Activity: Greater than 73.81
CBP Low: Less than 7.71fM
%Activity: Less than 0.00

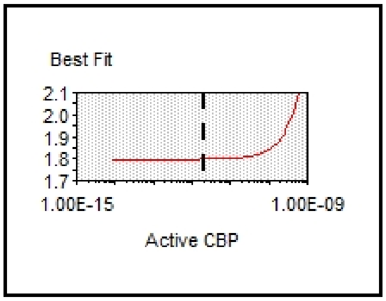

Data Traces(x)

Cycles: 1
Incubation delay (min): 0
Mix Time:

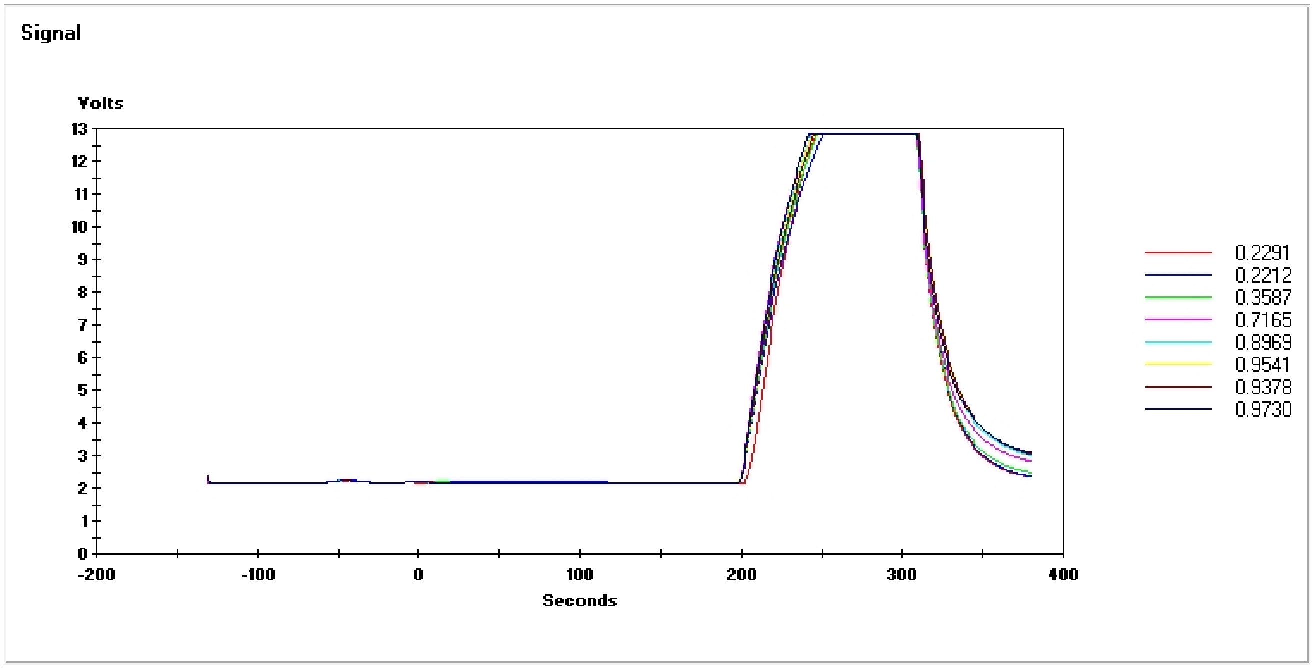

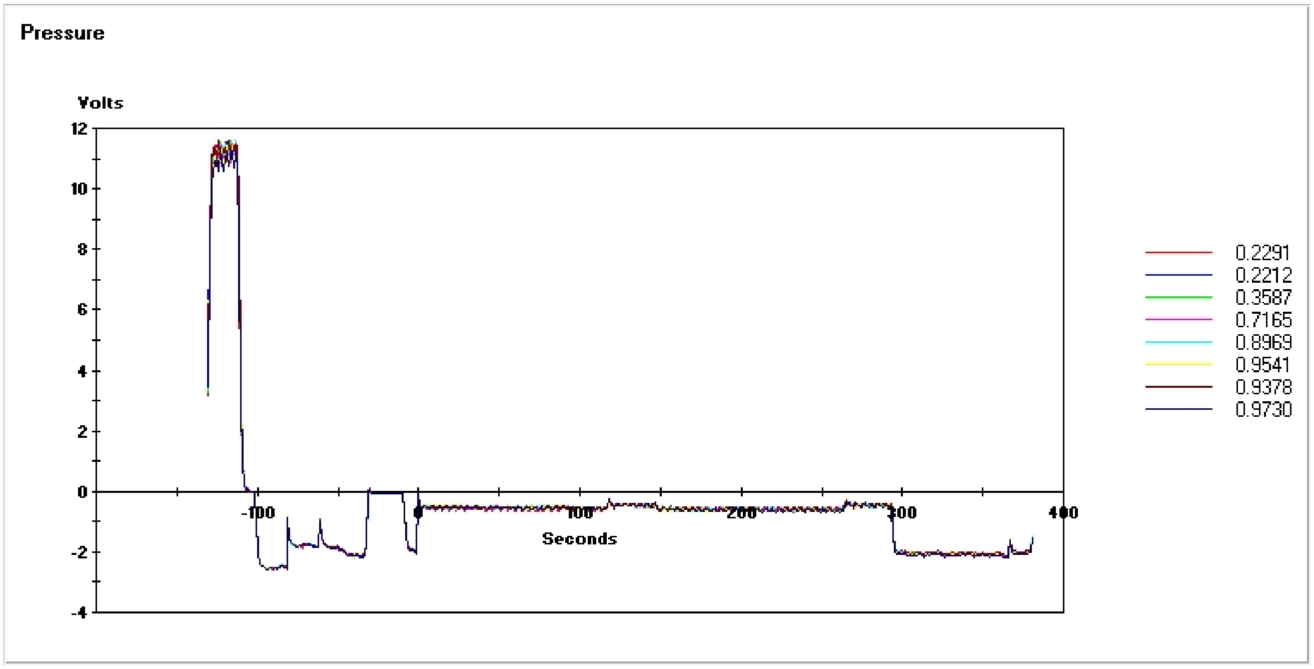

Supplement: S3 Data — (ZIP) [file pone.0273512.s005.zip › IgG KD measurements KinExA/RF 28H4 vs F4 HC-MBP.pdf]
