# Supplementary material for: Multicolor fluorescence activated cell sorting to generate humanized monoclonal antibody binding seven subtypes of BoNT/F
Source: PLoS One. 2022 Sep 1;17(9):e0273512. doi: 10.1371/journal.pone.0273512 (PMC9436041; doi:10.1371/journal.pone.0273512)

Experiment(x)

**Experiment Name:**	RF 6F15.3 vs F5 HNHC	**Start Time:**	Thu Sep 15 15:39:17 2016
**Experiment Type:**	Equilibrium	**End Time:**	Thu Sep 15 17:27:15 2016
**Constant Binding Partner (CBP)**		**Buffer:**	PBS/BSA
**Molecular Concentration:**	8.00nM	**Label:**	6F5.4-647
**Valency:**	1	**Label Conc:**	0
**Binding Site Concentration:**	8.00nM		

Comments(x)

beads: 6F15.3 8/29/16

sample volume: 500 ul

detection: 6F5.4-647

CBP: 8 nM BoNT F5 HNHC 11/16/15

titrant: 6F15.3 IgG 7/28/16

titration: 7 samples: 400 nM - 400 fM (1:10); + CBP only

samples:

1) NSB

2) 100% (CBP only)

3-9) titration of 6F15.2 IgG

Timing(x)

Bead Handling (Custom Beads)

Draw Source	Time (sec)	Volume (uL)	Rate (mL/min)	Stir
Backflush	20	0	0.0000	
Buffer	20	500	1.5000	✓
Particle Reservoir 1	20	333	1.0000	✓
Buffer	30	500	1.0000	
Waste	2	8	0.2500	
Buffer	20	0	0.0000	
Buffer	9	150	1.0000	

Sample Timing

Draw Source	Time (sec)	Volume (uL)	Rate (mL/min)	Time Stamp
Sample Set 1,309-316	120	500	0.2500	
Buffer	30	125	0.2500	
Rack 2: Tube 60	120	500	0.2500	
Buffer	30	125	0.2500	
Buffer	90	1500	1.0000	

Analysis(x)

Baseline / Endpoints:
5 to 10 (sec) from beginning
10 to 5 (sec) from end

Ignore	Binding Signal (V)	Concentration
✓	0.1143	0
	0.8382	0
	0.1450	400.00nM
	0.1578	40.00nM
	0.2314	4.00nM
	0.6756	400.00pM
	0.7630	40.00pM
	0.7835	4.00pM
	0.7542	400.00fM

**Kd:**	369.70pM
**Active CBP:**	1.78nM
**CBP % Activity:**	22.31
**Ratio:**	4.8268
**Sig 100%:**	0.79
**NSB:**	0.15
**%Error:**	3.24

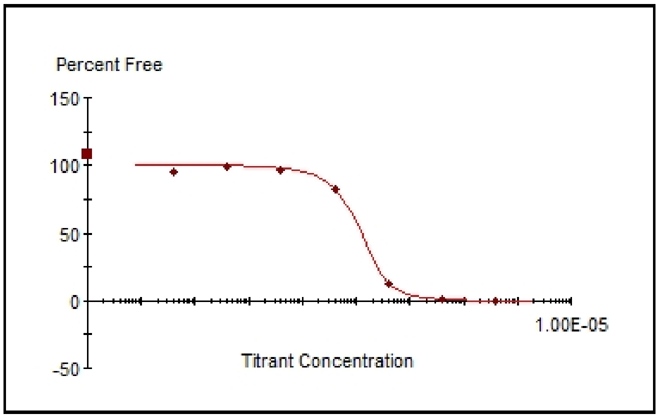

**Kd:**	369.70pM
**95% confidence interval**	
**Kd High:**	731.79pM
**Kd Low:**	135.82pM

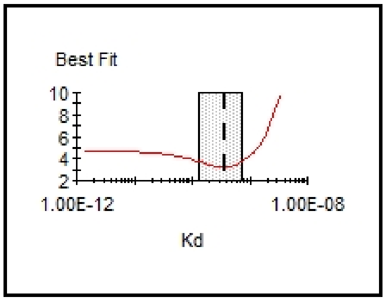

**Active CBP:**	1.78nM
**CBP %Activity:**	22.31
**95% confidence interval**	
**CBP High:**	3.79nM
**%Activity:**	47.34
**CBP Low:**	709.02pM
**%Activity:**	8.86

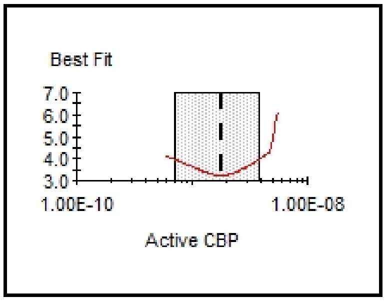

Data Traces(x)

Cycles: 1
Incubation delay (min): 0
Mix Time:

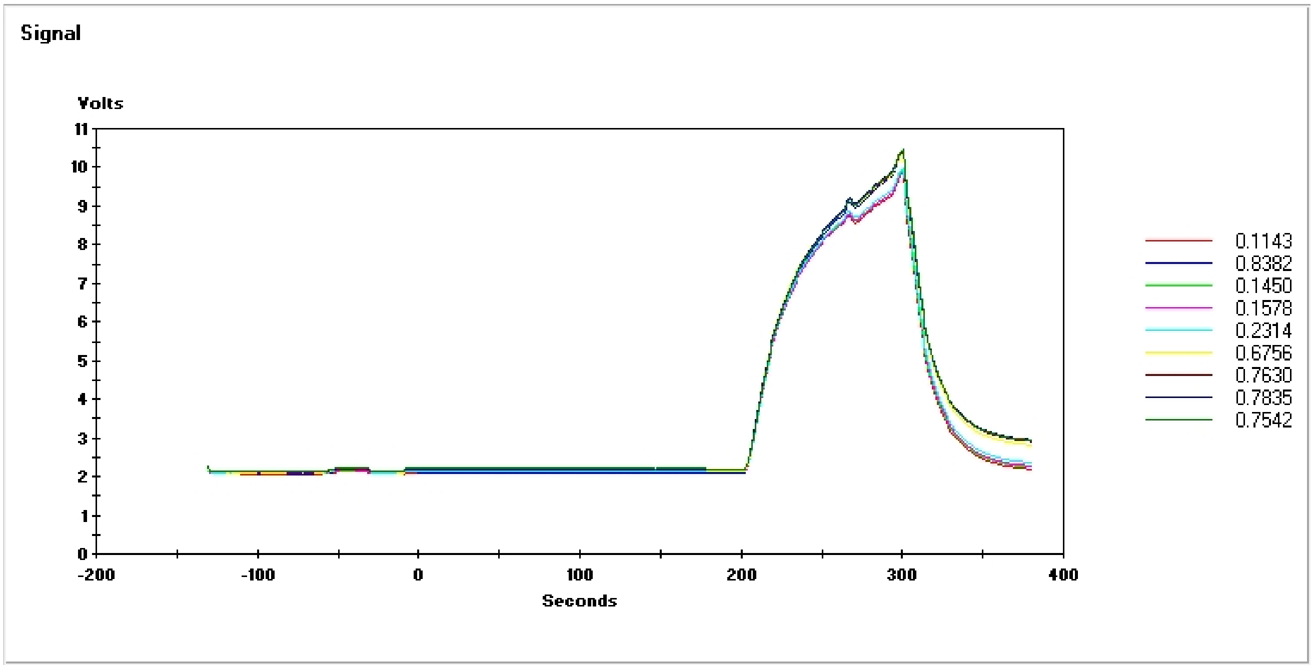

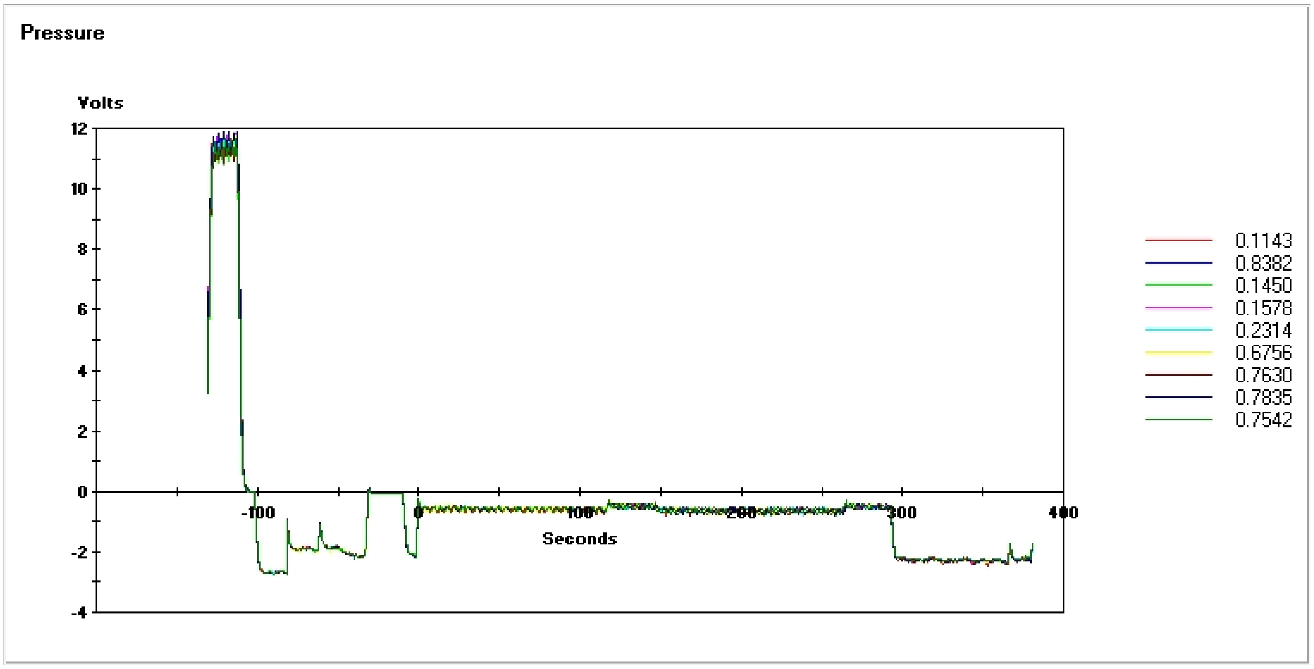

Supplement: S3 Data — (ZIP) [file pone.0273512.s005.zip › IgG KD measurements KinExA/RF 6F15.3 vs F5 HNHC.pdf]
