# Supplementary material for: Multicolor fluorescence activated cell sorting to generate humanized monoclonal antibody binding seven subtypes of BoNT/F
Source: PLoS One. 2022 Sep 1;17(9):e0273512. doi: 10.1371/journal.pone.0273512 (PMC9436041; doi:10.1371/journal.pone.0273512)

Experiment(x)

**Experiment Name:**	RF Hu6F15.6 vs F5 HNHC-MBP{3}	**Start Time:**	Wed Aug 30 14:59:42 2017
**Experiment Type:**	Equilibrium	**End Time:**	Wed Aug 30 19:34:17 2017
**Constant Binding Partner (CBP)**		**Buffer:**	PBS/BSA
**Molecular Concentration:**	100.00pM	**Label:**	6F5.4-647
**Valency:**	1	**Label Conc:**	0
**Binding Site Concentration:**	100.00pM		

Comments(x)

beads: Hu6F15.3 8/28/17

sample volume: 6 ml

detection: 6F5.4-647

CBP: 100 pM BoNT F5 HNHC-MBP 11/16/15

titrant: Hu6F15.6 IgG 2/3/17

titration: 7 samples: 40 nM - 40 fM (1:10)

samples:

1) NSB

2-8) titration

Timing(x)

Bead Handling (Custom Beads)

Draw Source	Time (sec)	Volume (uL)	Rate (mL/min)	Stir
Backflush	20	0	0.0000	
Buffer	20	500	1.5000	✓
Particle Reservoir 1	18	300	1.0000	✓
Buffer	30	500	1.0000	
Waste	2	8	0.2500	
Buffer	20	0	0.0000	
Buffer	9	150	1.0000	

Sample Timing

Draw Source	Time (sec)	Volume (uL)	Rate (mL/min)	Time Stamp
Sample Set 1,208-214	1440	6000	0.2500	
Buffer	30	125	0.2500	
Rack 2: Tube 60	120	500	0.2500	
Buffer	30	125	0.2500	
Buffer	90	1500	1.0000	

Analysis(x)

Baseline / Endpoints:
5 to 10 (sec) from beginning
10 to 5 (sec) from end

	Binding	
Ignore	Signal (V)	Concentration
	0.0785	4.00uM
	0.1305	40.00nM
	0.3093	4.00nM
	0.4479	400.00pM
	0.4763	40.00pM
	0.4749	4.00pM
	0.4723	400.00fM
	0.4772	40.00fM

**Kd:**	5.57nM
**Active CBP:**	5.51pM
**CBP %Activity:**	5.51
**Ratio:**	0.0010
**Sig 100%:**	0.48
**NSB:**	0.08
**%Error:**	0.54

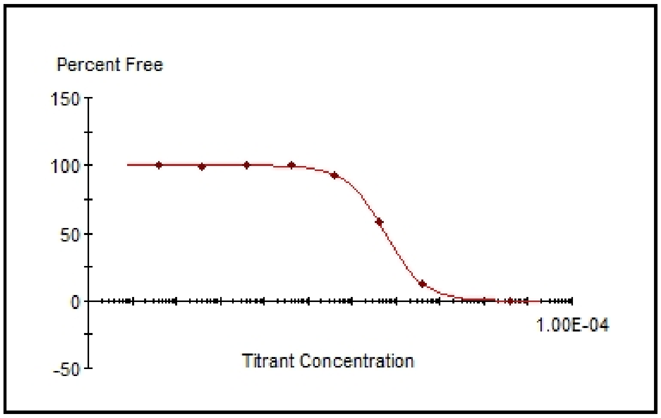

**Kd:**	5.57nM
**95% confidence interval**	
**Kd High:**	5.79nM
**Kd Low:**	5.25nM

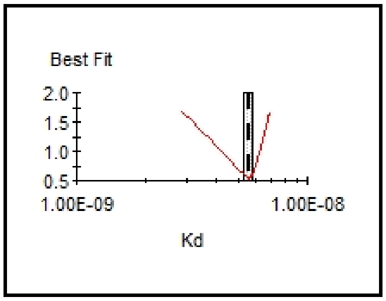

**Active CBP:**	5.51pM
**CBP %Activity:**	5.51
**95% confidence interval**	
**CBP High:**	710.46pM
**%Activity:**	710.46
**CBP Low:**	Less than 19.92fM
**%Activity:**	Less than 0.02

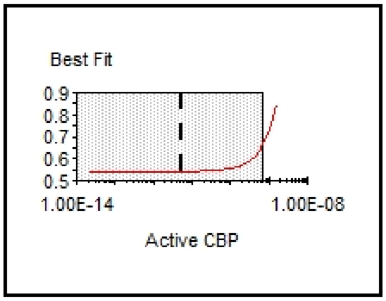

Data Traces(x)

Cycles: 1
Incubation delay (min): 0
Mix Time:

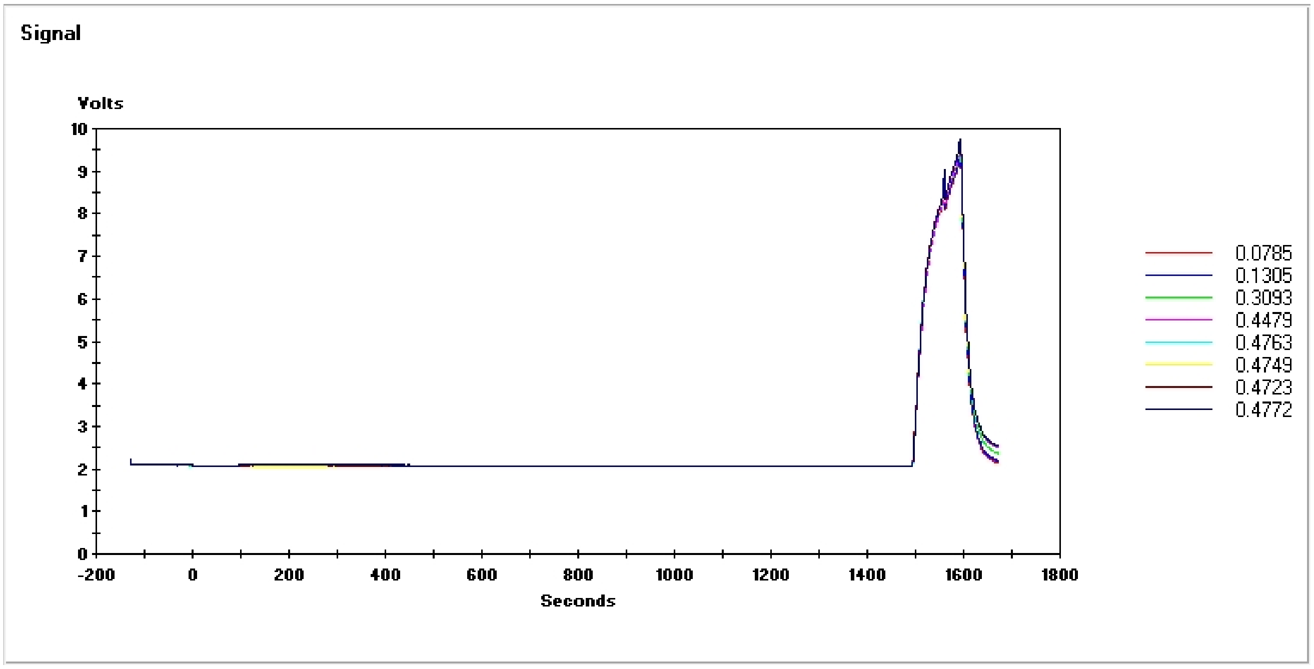

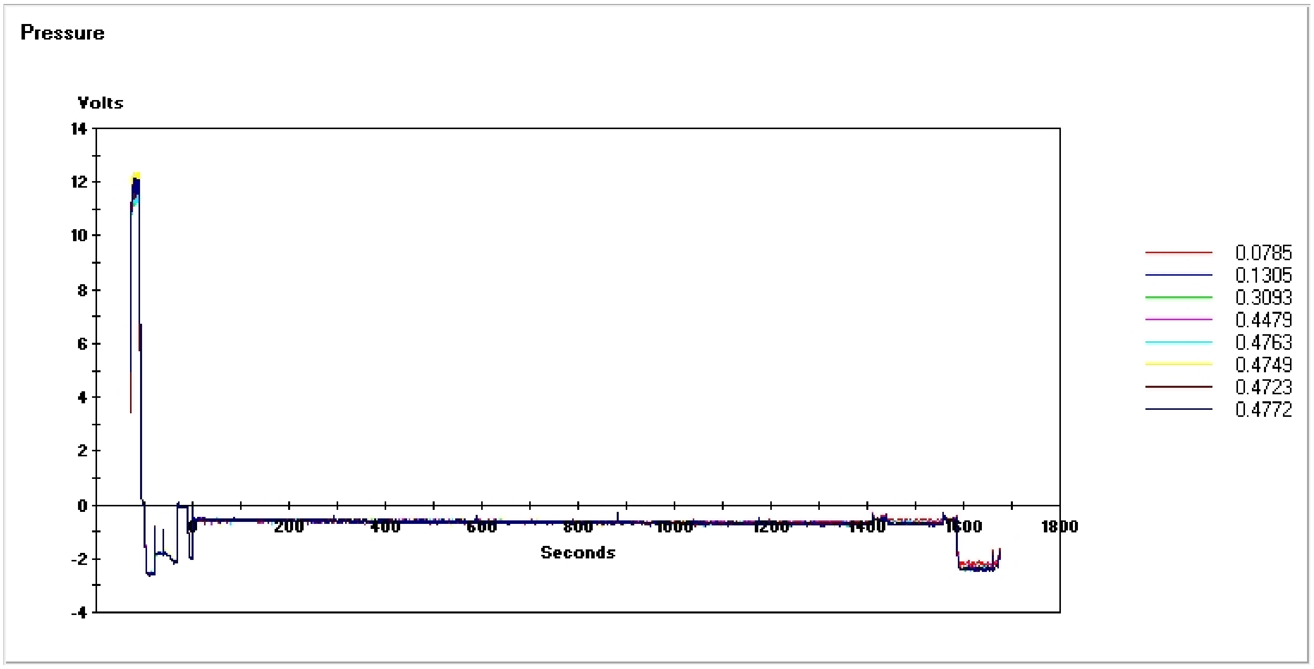

Supplement: S3 Data — (ZIP) [file pone.0273512.s005.zip › IgG KD measurements KinExA/RF Hu6F15.6 vs F5 HNHC-MBP{3}.pdf]
