# Supplementary material for: Multicolor fluorescence activated cell sorting to generate humanized monoclonal antibody binding seven subtypes of BoNT/F
Source: PLoS One. 2022 Sep 1;17(9):e0273512. doi: 10.1371/journal.pone.0273512 (PMC9436041; doi:10.1371/journal.pone.0273512)

**Experiment(x)**

| | | | |
|---|---|---|---|
| **Experiment Name:** | RF 6F15.3 vs F3HC{2} | **Start Time:** | Thu Sep 01 11:19:18 2016 |
| **Experiment Type:** | Equilibrium | **End Time:** | Thu Sep 01 13:07:14 2016 |
| **Constant Binding Partner (CBP)** | | **Buffer:** | PBS/BSA |
| **Molecular Concentration:** | 500.00pM | **Label:** | 6F8-647 |
| **Valency:** | 1 | **Label Conc:** | 0 |
| **Binding Site Concentration:** | 500.00pM | | |

**Comments(x)**

beads: 6F15.3 8/29/16

sample volume: 500 ul

detection: 6F8-647

CBP: 500 pM BoNT F2 HC-MBP 7/29/11

titrant: 6F15.3 IgG 7/28/16

titration: 7 samples: 40 nM - 40 fM (1:10); + CBP only

samples:

1) NSB

2) 100% (CBP only)

3-9) titration of 6F15.3 IgG

**Timing(x)**

**Bead Handling (Custom Beads)**

| Draw Source | Time (sec) | Volume (uL) | Rate (mL/min) | Stir |
|---|---|---|---|---|
| Backflush | 20 | 0 | 0.0000 | |
| Buffer | 20 | 500 | 1.5000 | ✓ |
| Particle Reservoir 1 | 20 | 333 | 1.0000 | ✓ |
| Buffer | 30 | 500 | 1.0000 | |
| Waste | 2 | 8 | 0.2500 | |
| Buffer | 20 | 0 | 0.0000 | |
| Buffer | 9 | 150 | 1.0000 | |

**Sample Timing**

| Draw Source | Time (sec) | Volume (uL) | Rate (mL/min) | Time Stamp |
|---|---|---|---|---|
| Sample Set 1,309-316 | 120 | 500 | 0.2500 | |
| Buffer | 30 | 125 | 0.2500 | |
| Rack 2: Tube 60 | 120 | 500 | 0.2500 | |
| Buffer | 30 | 125 | 0.2500 | |
| Buffer | 90 | 1500 | 1.0000 | |

**Analysis(x)**

**Baseline / Endpoints:**
5 to 10 (sec) from beginning
10 to 5 (sec) from end

| Ignore | Binding Signal (V) | Concentration |
|---|---|---|
| ✓ | 0.1610 | 0 |
| | 1.1906 | 0 |
| | 0.1868 | 40.00nM |
| | 0.2378 | 4.00nM |
| | 0.5806 | 400.00pM |
| | 1.0335 | 40.00pM |
| | 1.1700 | 4.00pM |
| | 1.1731 | 400.00fM |
| | 1.2064 | 40.00fM |

| | |
|---|---|
| **Kd:** | 254.03pM |
| **Active CBP:** | 245.90fM |
| **CBP % Activity:** | 0.05 |
| **Ratio:** | 0.0010 |
| **Sig 100%:** | 1.19 |
| **NSB:** | 0.18 |
| **%Error:** | 1.00 |

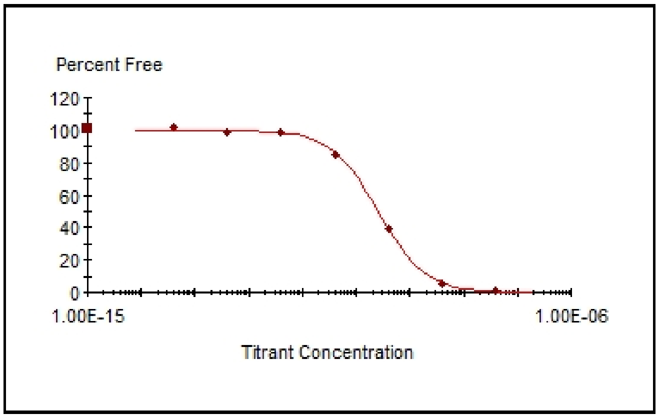


| | |
|---|---|
| **Kd:** | 254.03pM |
| **95% confidence interval** | |
| **Kd High:** | 274.63pM |
| **Kd Low:** | 232.80pM |

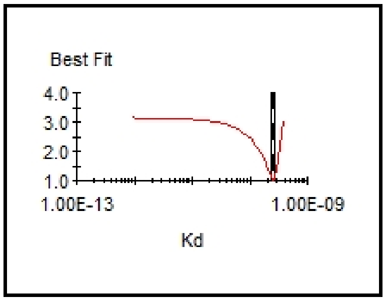


| | |
|---|---|
| **Active CBP:** | 245.90fM |
| **CBP %Activity:** | 0.05 |
| **95% confidence interval** | |
| **CBP High:** | 54.50pM |
| **%Activity:** | 10.90 |
| **CBP Low:** | Less than 888.36aM |
| **%Activity:** | Less than 0.00 |

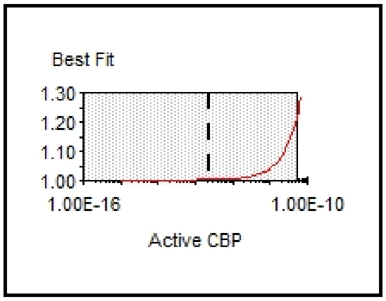

Data Traces(x)

Cycles: 1
Incubation delay (min): 0
Mix Time:

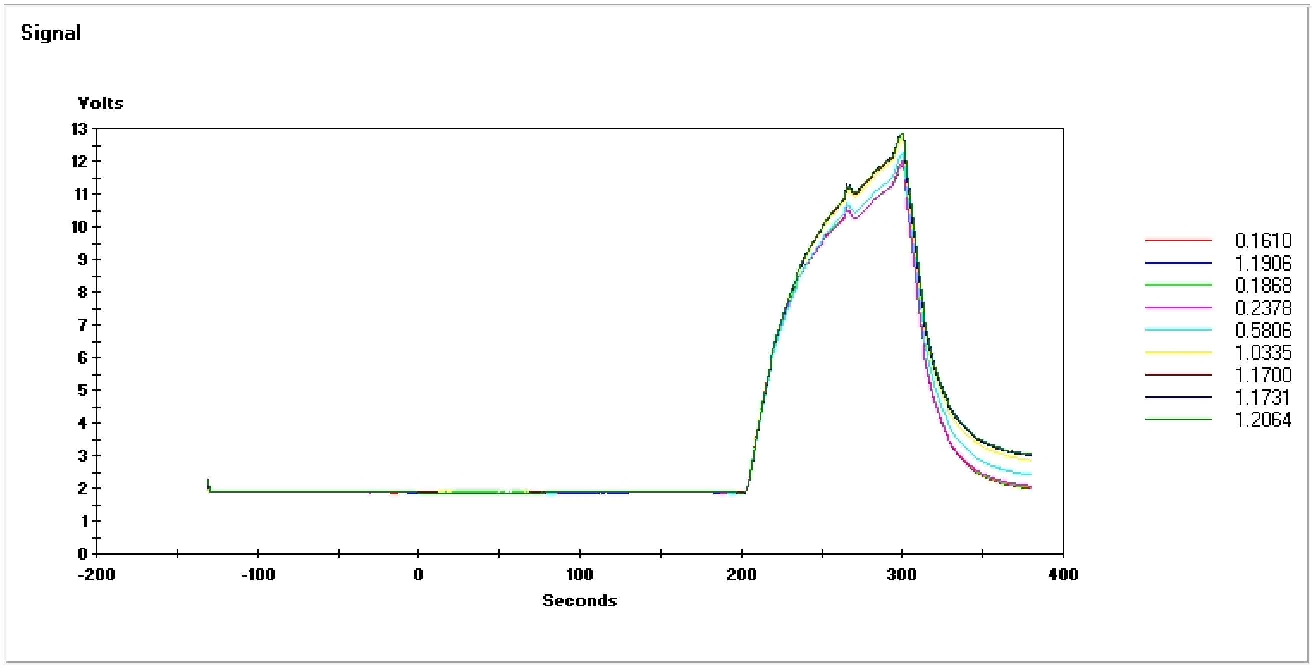


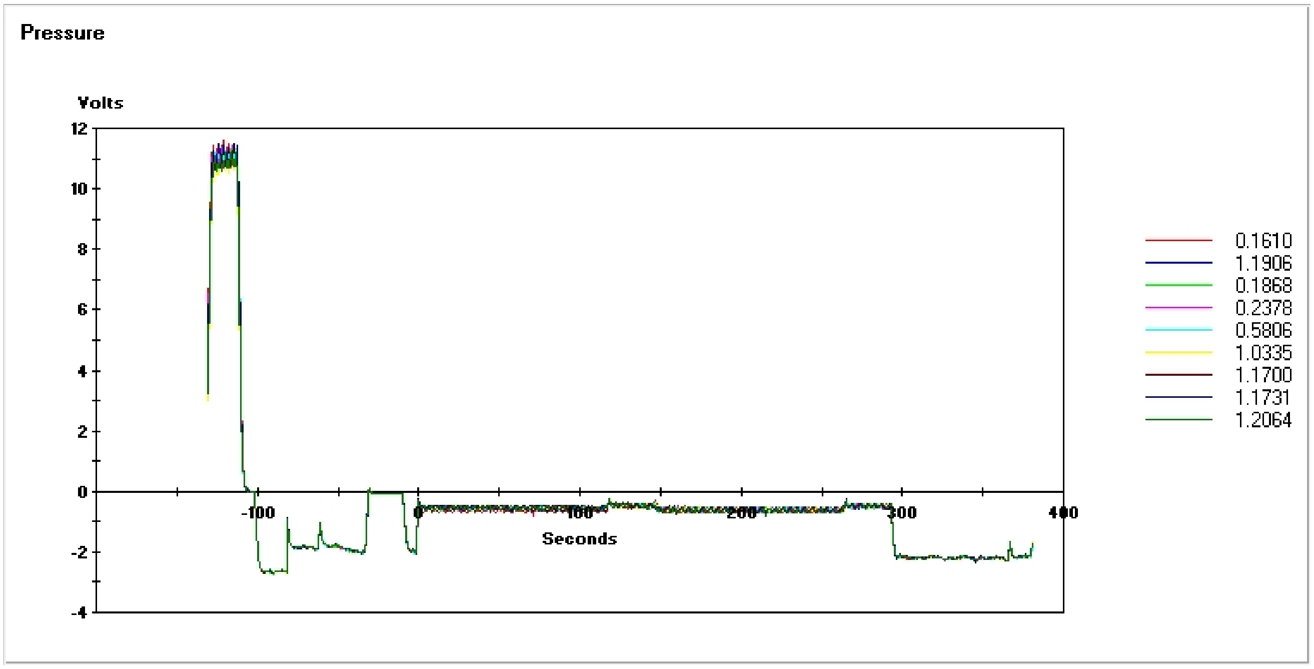

Supplement: S3 Data — (ZIP) [file pone.0273512.s005.zip › IgG KD measurements KinExA/RF 6F15.3 vs F3HC{2}.pdf]
