# Supplementary material for: Multicolor fluorescence activated cell sorting to generate humanized monoclonal antibody binding seven subtypes of BoNT/F
Source: PLoS One. 2022 Sep 1;17(9):e0273512. doi: 10.1371/journal.pone.0273512 (PMC9436041; doi:10.1371/journal.pone.0273512)

Experiment(x)

**Experiment Name:**	RF hu6F15.1 vs BoNT F1	**Start Time:**	Wed Nov 19 17:33:11 2014
**Experiment Type:**	Equilibrium	**End Time:**	Wed Nov 19 20:09:12 2014
**Constant Binding Partner (CBP)**		**Buffer:**	PBS/BSA
**Molecular Concentration:**	500.00pM	**Label:**	4E17.2-647
**Valency:**	1	**Label Conc:**	0
**Binding Site Concentration:**	500.00pM		

Comments(x)

beads: hu6F15.1 11/6/14

sample volume: 3 ml

detection: 4E17.2-647

receptor: 500 pM BoNT F1 100251 11/19/14

titrant: 6F15.1 IgG 11/5/14

titration: 5 samples: 50 nM -5 pM (1:10); + BoNT F1 only

samples:

1) NSB

2) 100% (BoNT F1 only)

3-7) titration of hu6F15.1 IgG

Timing(x)

Bead Handling (Custom Beads)

Draw Source	Time (sec)	Volume (uL)	Rate (mL/min)	Stir
Backflush	20	0	0.0000	
Buffer	20	500	1.5000	✓
Particle Reservoir 1	18	300	1.0000	✓
Buffer	30	500	1.0000	
Waste	2	8	0.2500	
Buffer	20	0	0.0000	
Buffer	9	150	1.0000	

Sample Timing

Draw Source	Time (sec)	Volume (uL)	Rate (mL/min)	Time Stamp
Sample Set 1,225-230	720	3000	0.2500	
Buffer	30	125	0.2500	
Standards: Tube 3	120	500	0.2500	
Buffer	30	125	0.2500	
Buffer	90	1500	1.0000	

Analysis(x)

Baseline / Endpoints:
45 to 50 (sec) from beginning
10 to 5 (sec) from end

Ignore	Binding Signal (V)	Concentration
✓	0.2184	NSB
	0.7421	0
	0.2567	50.00nM
	0.2768	5.00nM
	0.2906	500.00pM
	0.6746	50.00pM
	0.7406	5.00pM

**Kd:**	10.43pM
**Active CBP:**	329.00pM
**CBP % Activity:**	65.80
**Ratio:**	31.5423
**Sig 100%:**	0.74
**NSB:**	0.27
**%Error:**	1.11

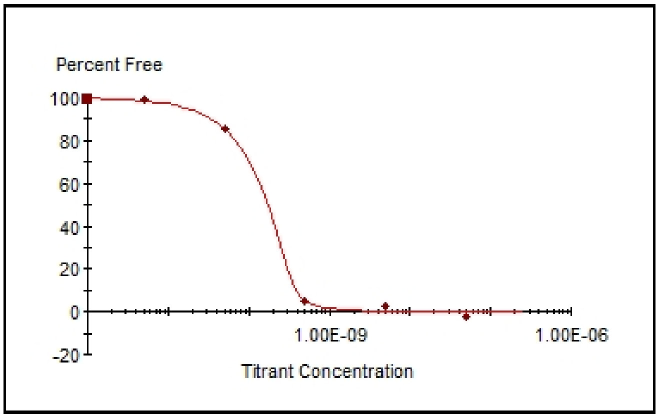

**Kd:**	10.43pM
**95% confidence interval**	
**Kd High:**	16.37pM
**Kd Low:**	5.65pM

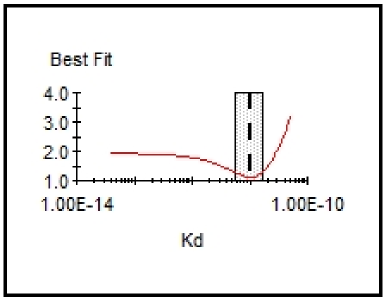

**Active CBP:**	329.00pM
**CBP %Activity:**	65.80
**95% confidence interval**	
**CBP High:**	405.41pM
**%Activity:**	81.08
**CBP Low:**	275.15pM
**%Activity:**	55.03

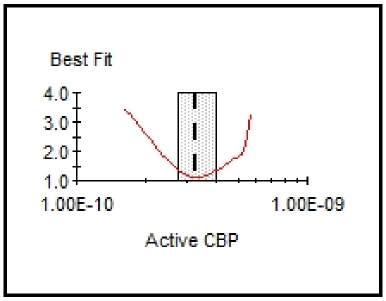

Data Traces(x)

Cycles: 1
Incubation delay (min): 0
Mix Time:

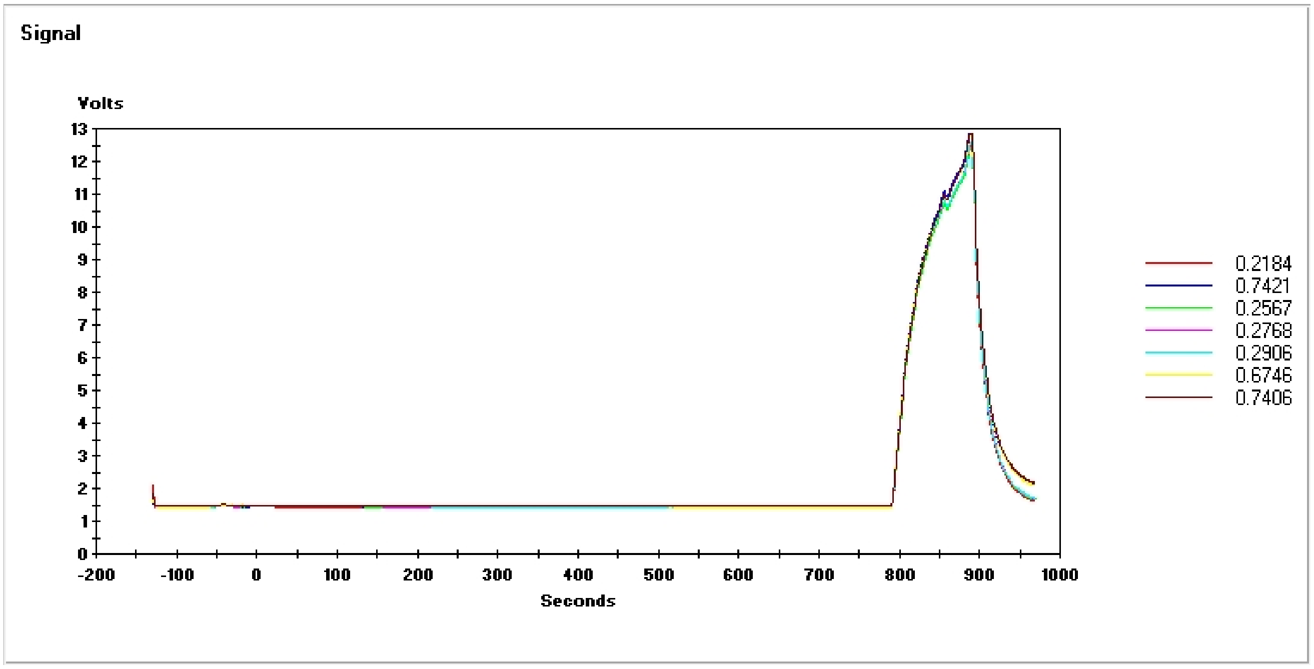

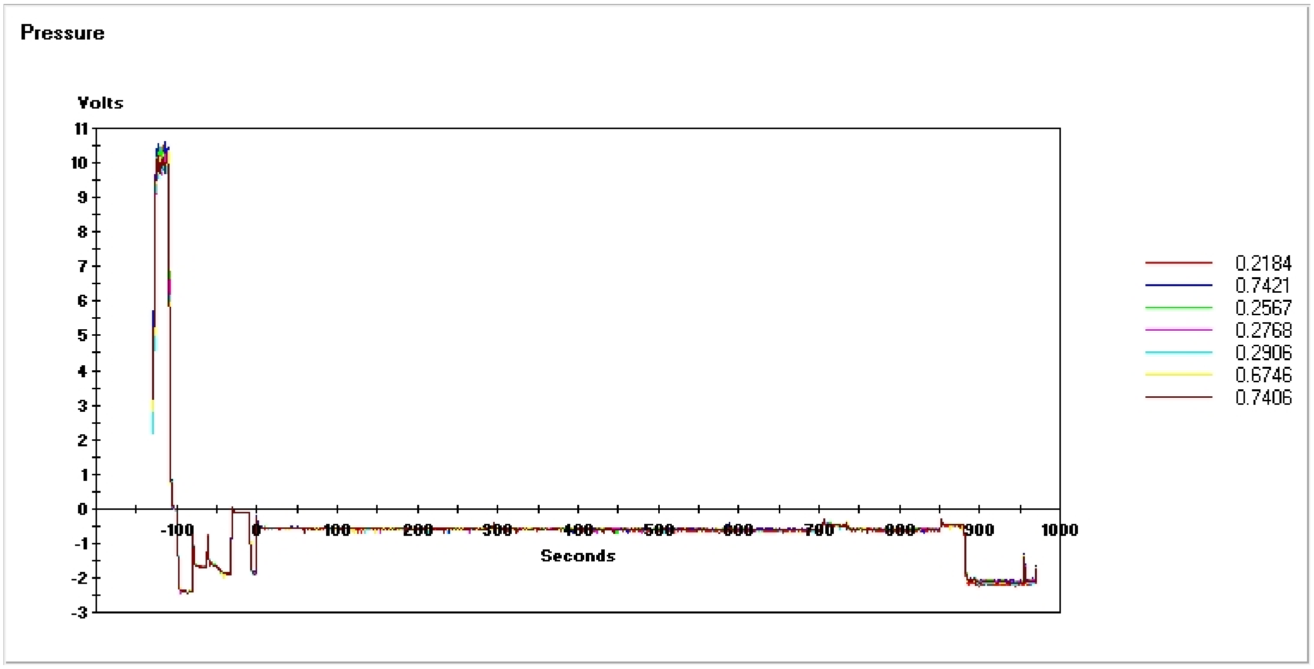

Supplement: S3 Data — (ZIP) [file pone.0273512.s005.zip › IgG KD measurements KinExA/RF hu6F15.1 vs BoNT F1.pdf]
