# Supplementary material for: Multicolor fluorescence activated cell sorting to generate humanized monoclonal antibody binding seven subtypes of BoNT/F
Source: PLoS One. 2022 Sep 1;17(9):e0273512. doi: 10.1371/journal.pone.0273512 (PMC9436041; doi:10.1371/journal.pone.0273512)

Experiment(x)

**Experiment Name:**	RF 6F15.3 vs F1	**Start Time:**	Mon Oct 24 10:56:54 2016
**Experiment Type:**	Equilibrium	**End Time:**	Mon Oct 24 16:06:13 2016
**Constant Binding Partner (CBP)**		**Buffer:**	PBS/BSA
**Molecular Concentration:**	100.00pM	**Label:**	6F5.4-647
**Valency:**	1	**Label Conc:**	0
**Binding Site Concentration:**	100.00pM		

Comments(x)

beads: 6F15.3 9/21/16

sample volume: 6 ml

detection: 6F5.4-647

CBP: 100 pM BoNT F1 100251 10/21/16

titrant: 6F15.3 IgG 7/28/16

titration: 7 samples: 10 nM - 10 fM (1:10); + CBP only

samples:

1) NSB

2) 100% (CBP only)

3-9) titration of 6F15.3 IgG

Timing(x)

Bead Handling (Custom Beads)

Draw Source	Time (sec)	Volume (uL)	Rate (mL/min)	Stir
Backflush	20	0	0.0000	
Buffer	20	500	1.5000	✓
Particle Reservoir 1	20	333	1.0000	✓
Buffer	30	500	1.0000	
Waste	2	8	0.2500	
Buffer	20	0	0.0000	
Buffer	9	150	1.0000	

Sample Timing

Draw Source	Time (sec)	Volume (uL)	Rate (mL/min)	Time Stamp
Sample Set 1,201-208	1440	6000	0.2500	
Buffer	30	125	0.2500	
Rack 2: Tube 60	120	500	0.2500	
Buffer	30	125	0.2500	
Buffer	90	1500	1.0000	

Analysis(x)

Baseline / Endpoints:
5 to 10 (sec) from beginning
10 to 5 (sec) from end

	**Binding**	
**Ignore**	**Signal (V)**	**Concentration**
✓	0.1137	0
	1.0722	0
	0.1342	10.00nM
	0.1727	1.00nM
	0.5122	100.00pM
	0.9956	10.00pM
	1.0709	1.00pM
	1.0588	100.00fM
	1.0596	10.00fM

**Kd:**	38.35pM
**Active CBP:**	72.38pM
**CBP %Activity:**	72.38
**Ratio:**	1.8873
**Sig 100%:**	1.08
**NSB:**	0.13
**%Error:**	0.26

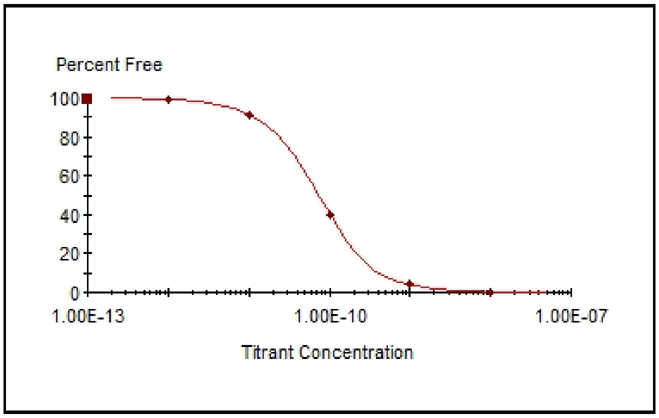

**Kd:**	38.35pM
**95% confidence interval**	
**Kd High:**	42.07pM
**Kd Low:**	34.47pM

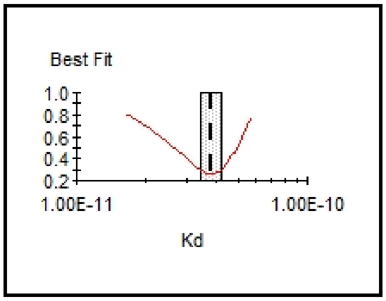

**Active CBP:**	72.38pM
**CBP %Activity:**	72.38
**95% confidence interval**	
**CBP High:**	84.07pM
**%Activity:**	84.07
**CBP Low:**	61.39pM
**%Activity:**	61.39

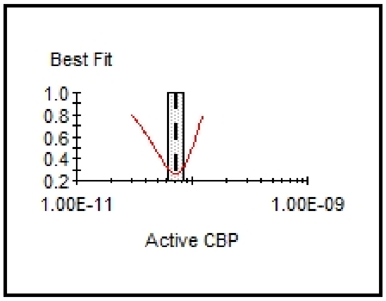

Data Traces(x)

Cycles: 1
Incubation delay (min): 0
Mix Time:

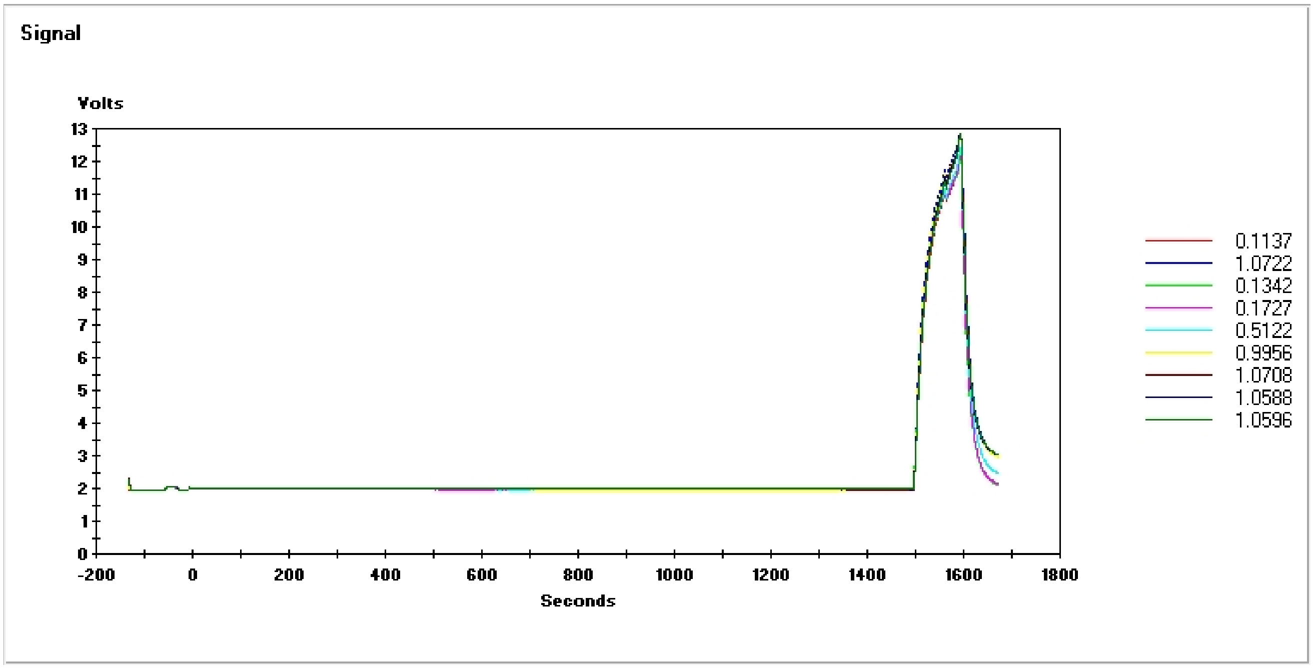

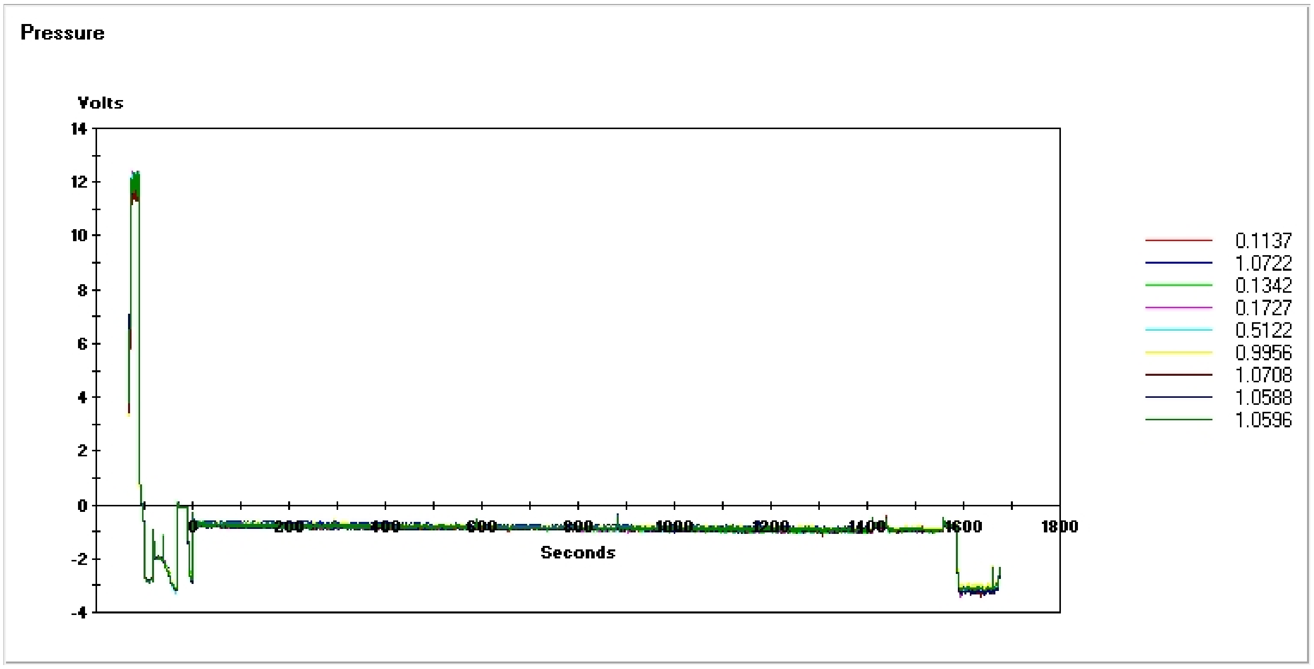

Supplement: S3 Data — (ZIP) [file pone.0273512.s005.zip › IgG KD measurements KinExA/RF 6F15.3 vs F1.pdf]
