# Supplementary material for: Multicolor fluorescence activated cell sorting to generate humanized monoclonal antibody binding seven subtypes of BoNT/F
Source: PLoS One. 2022 Sep 1;17(9):e0273512. doi: 10.1371/journal.pone.0273512 (PMC9436041; doi:10.1371/journal.pone.0273512)

**Experiment(x)**

| | | | |
|---|---|---|---|
| **Experiment Name:** | RF Hu6F15.6 vs F1 | **Start Time:** | Thu Sep 14 14:20:35 2017 |
| **Experiment Type:** | Equilibrium | **End Time:** | Thu Sep 14 18:55:12 2017 |
| **Constant Binding Partner (CBP)** | | **Buffer:** | PBS/BSA |
| **Molecular Concentration:** | 80.00pM | **Label:** | 6F5.4-647 |
| **Valency:** | 1 | **Label Conc:** | 0 |
| **Binding Site Concentration:** | 80.00pM | | |

**Comments(x)**

beads: Hu6F15.3 8/28/17

sample volume: 6 ml

detection: 6F5.4-647

CBP: 80 pM BoNT F1 holotoxin 100251 9/12/17

titrant: Hu6F15.6 IgG 2/3/17

titration: 7 samples: 100 nM - 100 fM (1:10)

samples:

1) NSB

2-8) titration
beads: Hu6F15.3 9/8/17

**Timing(x)**

**Bead Handling (Custom Beads)**

| Draw Source | Time (sec) | Volume (uL) | Rate (mL/min) | Stir |
|---|---|---|---|---|
| Backflush | 20 | 0 | 0.0000 | |
| Buffer | 20 | 500 | 1.5000 | ✓ |
| Particle Reservoir 2 | 18 | 300 | 1.0000 | ✓ |
| Buffer | 30 | 500 | 1.0000 | |
| Waste | 2 | 8 | 0.2500 | |
| Buffer | 20 | 0 | 0.0000 | |
| Buffer | 9 | 150 | 1.0000 | |

**Sample Timing**

| Draw Source | Time (sec) | Volume (uL) | Rate (mL/min) | Time Stamp |
|---|---|---|---|---|
| Sample Set 1,215-221 | 1440 | 6000 | 0.2500 | |
| Buffer | 30 | 125 | 0.2500 | |
| Rack 2: Tube 59 | 120 | 500 | 0.2500 | |
| Buffer | 30 | 125 | 0.2500 | |
| Buffer | 90 | 1500 | 1.0000 | |

**Analysis(x)**

**Baseline / Endpoints:**
5 to 10 (sec) from beginning
10 to 5 (sec) from end

| Ignore | Binding Signal (V) | Concentration |
|---|---|---|
| ✓ | 0.0269 | NSB |
| | 0.0671 | 100.00nM |
| | 0.0569 | 10.00nM |
| | 0.1122 | 1.00nM |
| | 0.2231 | 100.00pM |
| | 0.2724 | 10.00pM |
| | 0.2650 | 1.00pM |
| | 0.2788 | 100.00fM |

| | |
|---|---|
| **Kd:** | 302.41pM |
| **Active CBP:** | 38.71pM |
| **CBP % Activity:** | 48.39 |
| **Ratio:** | 0.1280 |
| **Sig 100%:** | 0.27 |
| **NSB:** | 0.06 |
| **%Error:** | 2.59 |

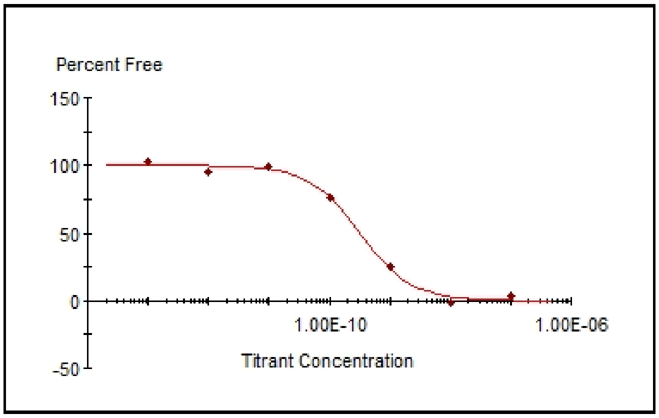


| | |
|---|---|
| **Kd:** | 302.41pM |
| **95% confidence interval** | |
| **Kd High:** | 389.44pM |
| **Kd Low:** | 201.15pM |

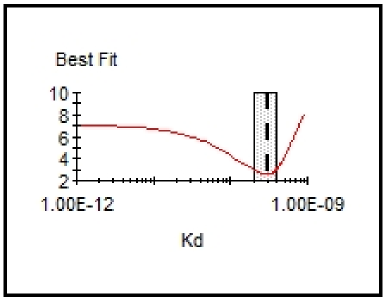


| | |
|---|---|
| **Active CBP:** | 38.71pM |
| **CBP %Activity:** | 48.39 |
| **95% confidence interval** | |
| **CBP High:** | 304.20pM |
| **%Activity:** | 380.26 |
| **CBP Low:** | Less than 139.86fM |
| **%Activity:** | Less than 0.17 |

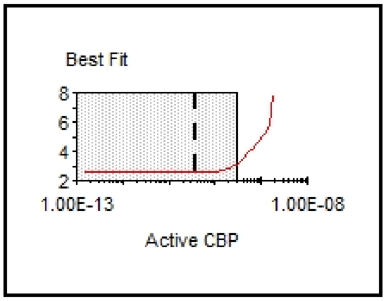

Data Traces(x)

Cycles: 1
Incubation delay (min): 0
Mix Time:

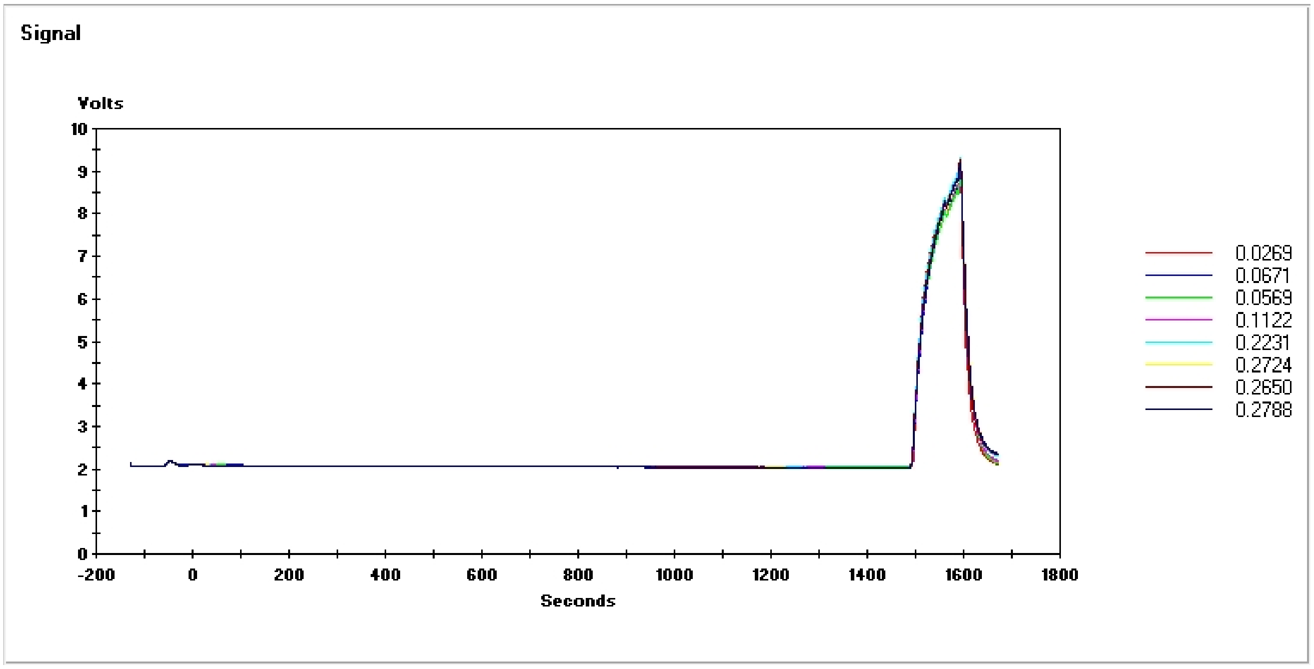


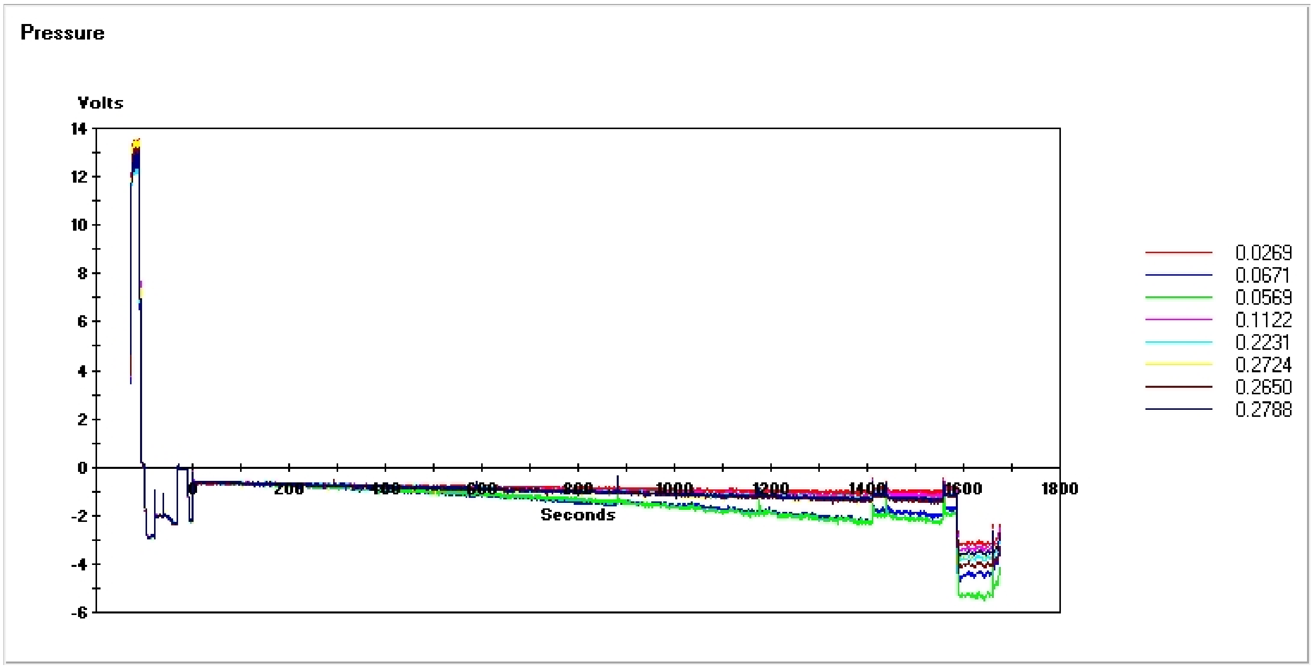

Supplement: S3 Data — (ZIP) [file pone.0273512.s005.zip › IgG KD measurements KinExA/RF Hu6F15.6 vs F1.pdf]
