# Supplementary material for: Multicolor fluorescence activated cell sorting to generate humanized monoclonal antibody binding seven subtypes of BoNT/F
Source: PLoS One. 2022 Sep 1;17(9):e0273512. doi: 10.1371/journal.pone.0273512 (PMC9436041; doi:10.1371/journal.pone.0273512)

Experiment(x)

**Experiment Name:**	RF 6F15.3 vs F6HC	**Start Time:**	Tue Sep 13 18:31:47 2016
**Experiment Type:**	Equilibrium	**End Time:**	Tue Sep 13 20:19:44 2016
**Constant Binding Partner (CBP)**		**Buffer:**	PBS/BSA
**Molecular Concentration:**	2.00nM	**Label:**	6F8-647
**Valency:**	1	**Label Conc:**	0
**Binding Site Concentration:**	2.00nM		

Comments(x)

beads: 6F15.3 8/29/16

sample volume: 500 ul

detection: 6F8-647

CBP: 2 nM BoNT F6 HC-MBP 7/29/11

titrant: 6F15.3 IgG 7/28/16

titration: 7 samples: 100 nM - 100 fM (1:10); + CBP only

samples:

1) NSB

2) 100% (CBP only)

3-9) titration of 6F15.3 IgG

Timing(x)

Bead Handling (Custom Beads)

Draw Source	Time (sec)	Volume (uL)	Rate (mL/min)	Stir
Backflush	20	0	0.0000	
Buffer	20	500	1.5000	✓
Particle Reservoir 1	20	333	1.0000	✓
Buffer	30	500	1.0000	
Waste	2	8	0.2500	
Buffer	20	0	0.0000	
Buffer	9	150	1.0000	

Sample Timing

Draw Source	Time (sec)	Volume (uL)	Rate (mL/min)	Time Stamp
Sample Set 1,309-316	120	500	0.2500	
Buffer	30	125	0.2500	
Rack 2: Tube 60	120	500	0.2500	
Buffer	30	125	0.2500	
Buffer	90	1500	1.0000	

Analysis(x)

Baseline / Endpoints:
5 to 10 (sec) from beginning
10 to 5 (sec) from end

Ignore	Binding Signal (V)	Concentration
✓	0.1673	0
	1.4903	0
	0.1772	100.00nM
	0.2138	10.00nM
	0.4216	1.00nM
	1.0882	100.00pM
	1.4228	10.00pM
	1.4749	1.00pM
	1.4706	100.00fM

**Kd:**	230.98pM
**Active CBP:**	564.52fM
**CBP % Activity:**	0.03
**Ratio:**	0.0024
**Sig 100%:**	1.48
**NSB:**	0.18
**%Error:**	0.41

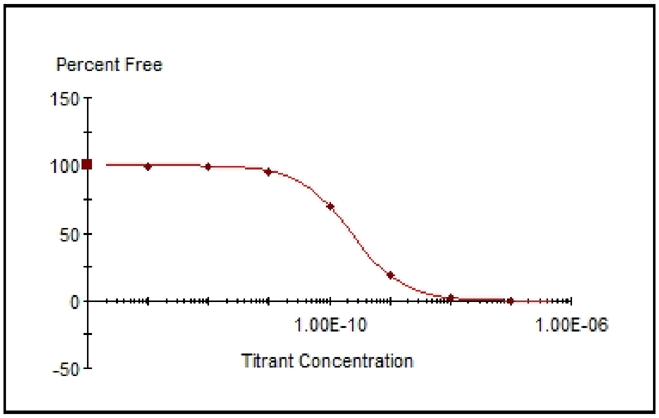

**Kd:**	230.98pM
**95% confidence interval**	
**Kd High:**	238.50pM
**Kd Low:**	214.45pM

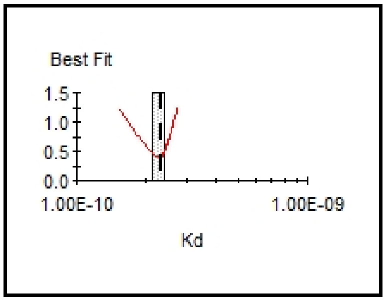

**Active CBP:**	564.52fM
**CBP %Activity:**	0.03
**95% confidence interval**	
**CBP High:**	34.67pM
**%Activity:**	1.73
**CBP Low:**	Less than 2.04fM
**%Activity:**	Less than 0.00

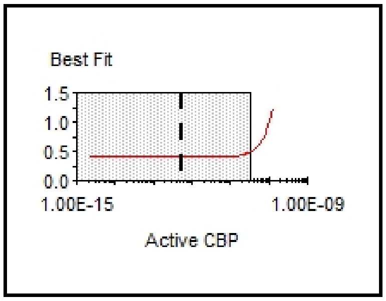

Data Traces(x)

Cycles: 1
Incubation delay (min): 0
Mix Time:

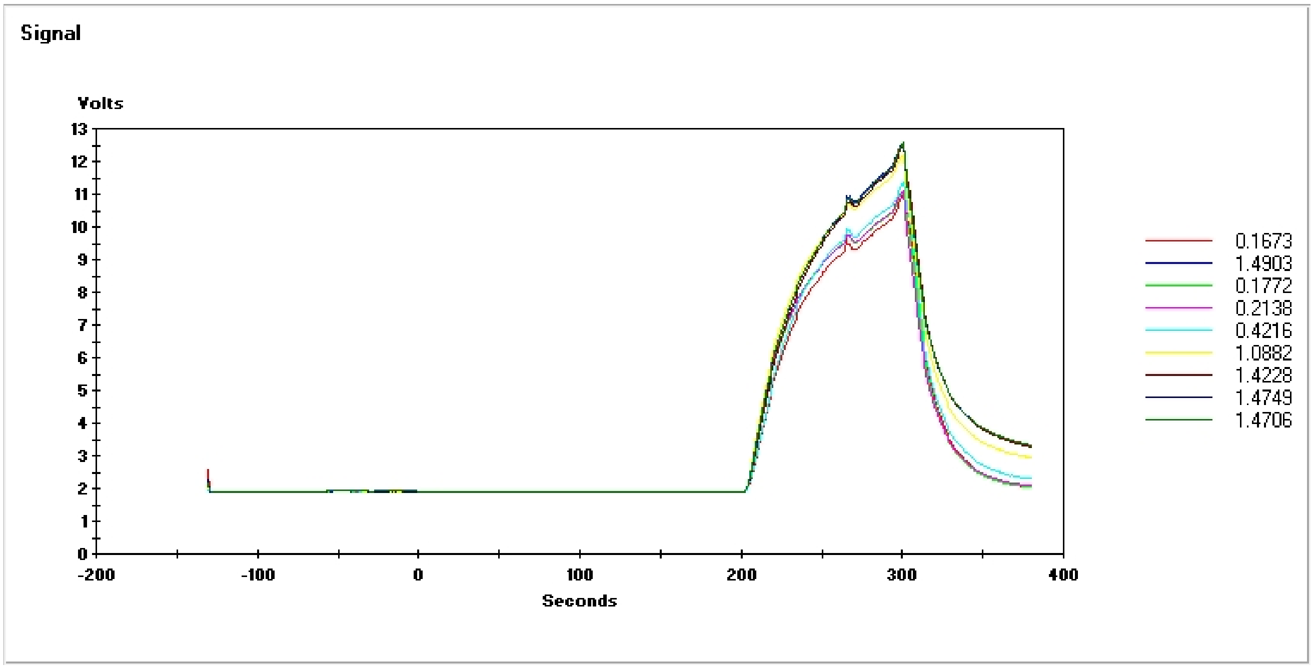

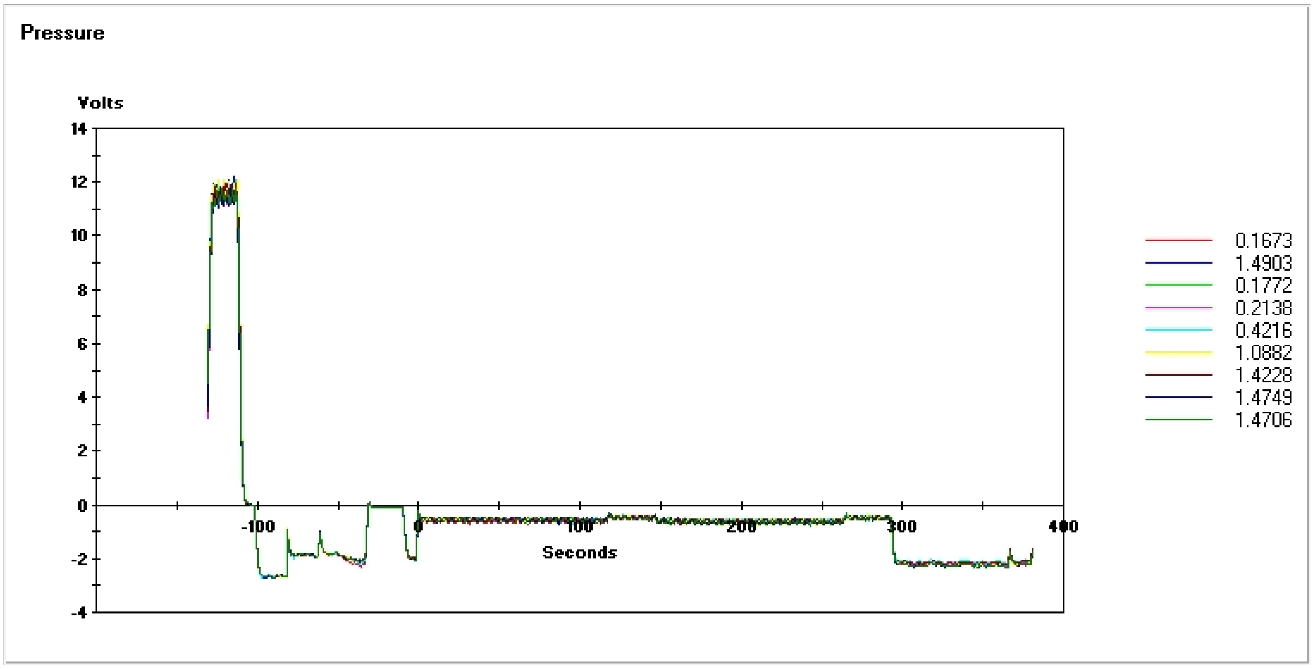

Supplement: S3 Data — (ZIP) [file pone.0273512.s005.zip › IgG KD measurements KinExA/RF 6F15.3 vs F6HC.pdf]
