# Supplementary material for: Multicolor fluorescence activated cell sorting to generate humanized monoclonal antibody binding seven subtypes of BoNT/F
Source: PLoS One. 2022 Sep 1;17(9):e0273512. doi: 10.1371/journal.pone.0273512 (PMC9436041; doi:10.1371/journal.pone.0273512)

Experiment(x)

**Experiment Name:**	RF Hu6F15.1 vs F7 HC-MBP	**Start Time:**	Sat Oct 07 12:59:20 2017
**Experiment Type:**	Equilibrium	**End Time:**	Sat Oct 07 14:34:58 2017
**Constant Binding Partner (CBP)**		**Buffer:**	PBS/BSA
**Molecular Concentration:**	10.00nM	**Label:**	6F3-647
**Valency:**	1	**Label Conc:**	0
**Binding Site Concentration:**	10.00nM		

Comments(x)

beads: Hu6F15.3 9/8/17

sample volume: 500 ul

detection: 6F3-647

CBP: 10 nM BoNT F7 HC-MBP 12/17/14

titrant: Hu6F15.1 IgG 3/21/15

titration: 5 samples: 2 uM - 2 pM (1:10)

samples:

1) NSB

2-8) titration
titration: 7 samples: 2 uM - 2 pM (1:10)

Timing(x)

Bead Handling (Custom Beads)

Draw Source	Time (sec)	Volume (uL)	Rate (mL/min)	Stir
Backflush	20	0	0.0000	
Buffer	20	500	1.5000	✓
Particle Reservoir 1	18	300	1.0000	✓
Buffer	30	500	1.0000	
Waste	2	8	0.2500	
Buffer	20	0	0.0000	
Buffer	9	150	1.0000	

Sample Timing

Draw Source	Time (sec)	Volume (uL)	Rate (mL/min)	Time Stamp
Sample Set 1,301-307	120	500	0.2500	
Buffer	30	125	0.2500	
Rack 2: Tube 60	120	500	0.2500	
Buffer	30	125	0.2500	
Buffer	90	1500	1.0000	

Analysis(x)

Baseline / Endpoints:

175 to 100 (sec) from beginning

10 to 5 (sec) from end

Binding

**Ignore**	**Signal (V)**	**Concentration**
✓	0.0232	NSB
	0.0349	2.00uM
	0.0639	200.00nM
	0.2420	20.00nM
	0.9003	2.00nM
	1.1051	200.00pM
	1.1299	20.00pM
	1.1443	2.00pM

**Kd:**	3.76nM
**Active CBP:**	4.47nM
**CBP %Activity:**	44.66
**Ratio:**	1.1867
**Sig 100%:**	1.14
**NSB:**	0.04
**%Error:**	0.41

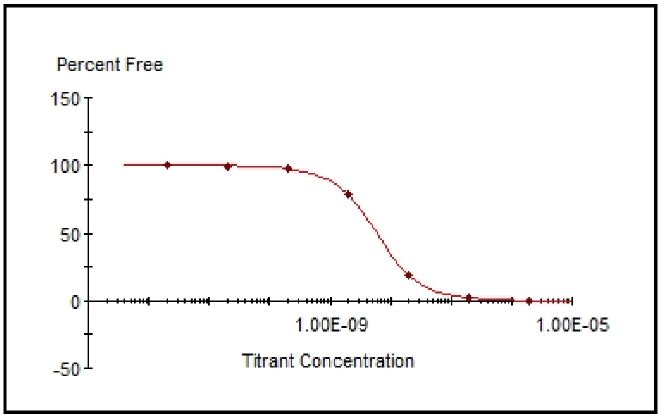

**Kd:**	3.76nM
**95% confidence interval**	
**Kd High:**	4.04nM
**Kd Low:**	3.50nM

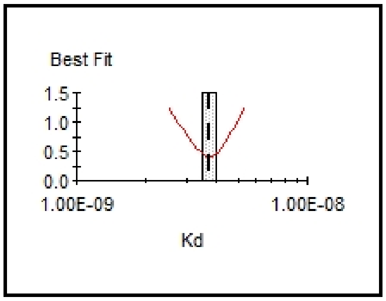

**Active CBP:**	4.47nM
**CBP %Activity:**	44.66
**95% confidence interval**	
**CBP High:**	5.17nM
**%Activity:**	51.74
**CBP Low:**	3.76nM
**%Activity:**	37.63

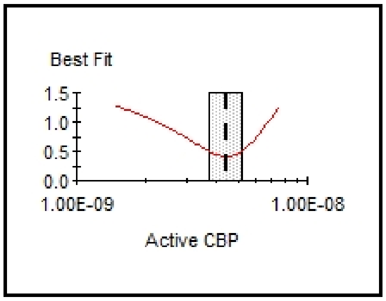

Data Traces(x)

Cycles: 1
Incubation delay (min): 0
Mix Time:

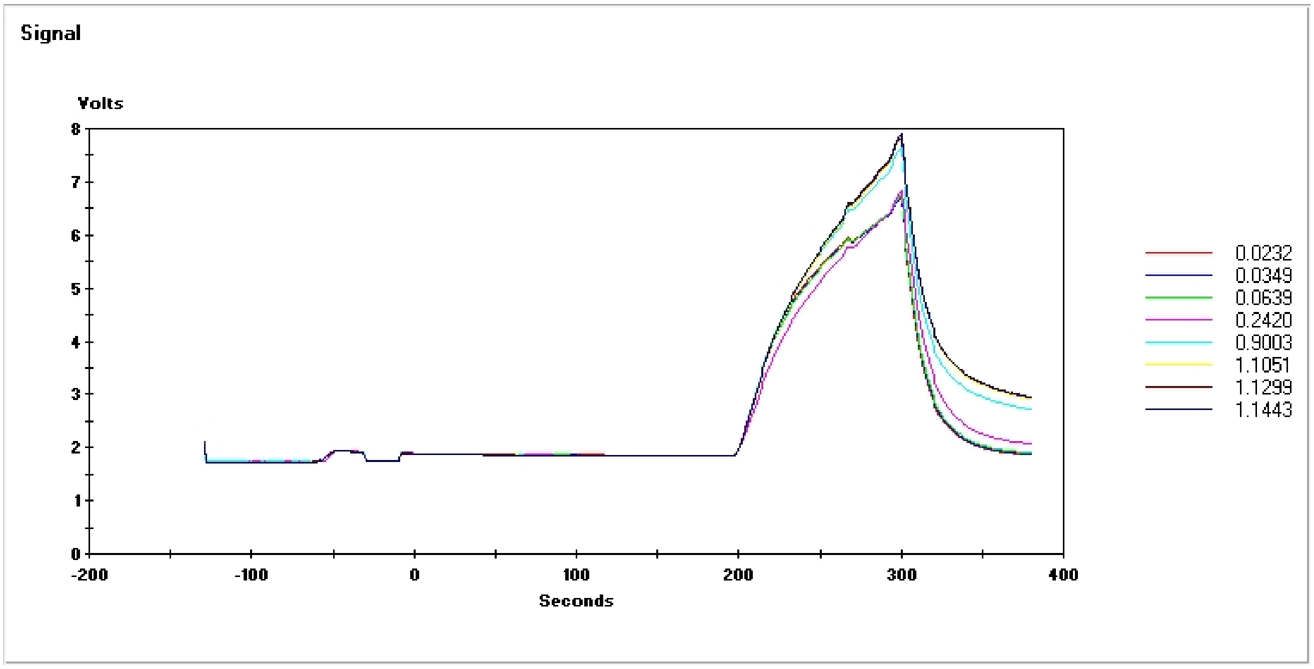

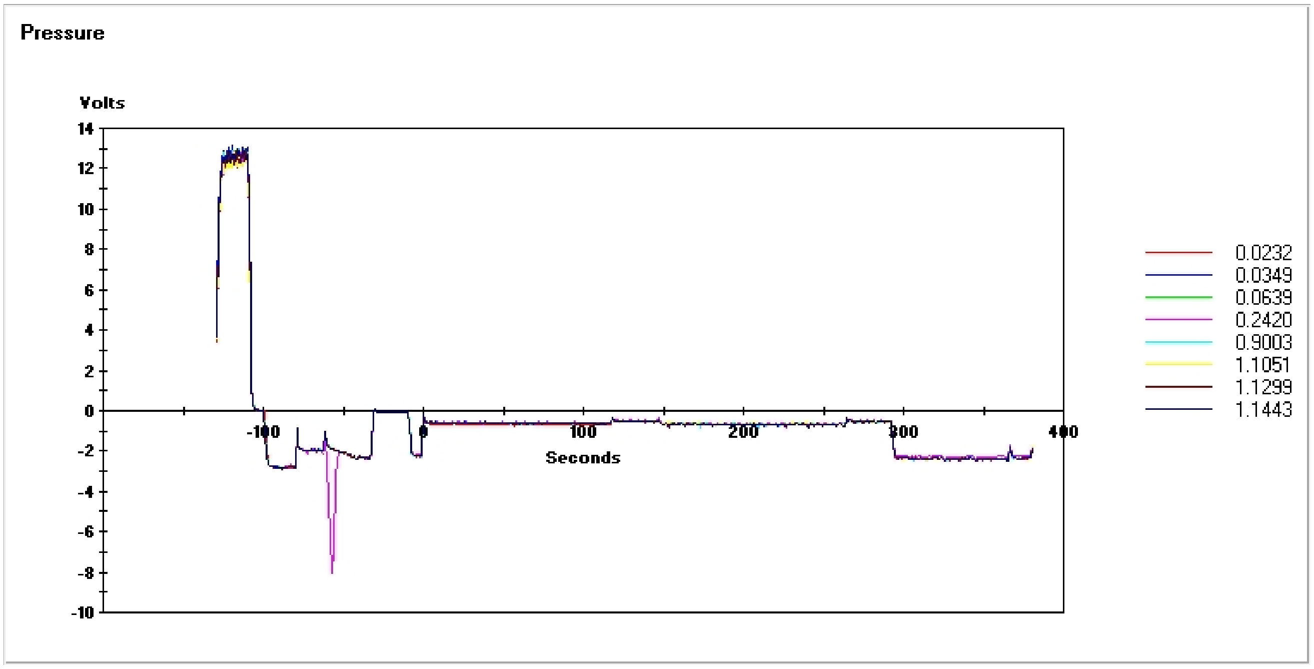

Supplement: S3 Data — (ZIP) [file pone.0273512.s005.zip › IgG KD measurements KinExA/RF Hu6F15.1 vs F7 HC-MBP.pdf]
