# Supplementary material for: Multicolor fluorescence activated cell sorting to generate humanized monoclonal antibody binding seven subtypes of BoNT/F
Source: PLoS One. 2022 Sep 1;17(9):e0273512. doi: 10.1371/journal.pone.0273512 (PMC9436041; doi:10.1371/journal.pone.0273512)

Experiment(x)

**Experiment Name:**	RF Hu6F15.6 vs F4 HC-MBP	**Start Time:**	Mon Aug 14 16:57:15 2017
**Experiment Type:**	Equilibrium	**End Time:**	Mon Aug 14 21:32:06 2017
**Constant Binding Partner (CBP)**		**Buffer:**	PBS/BSA
**Molecular Concentration:**	60.00pM	**Label:**	6F8-647
**Valency:**	1	**Label Conc:**	0
**Binding Site Concentration:**	60.00pM		

Comments(x)

beads: Hu6F15.3 8/10/17

sample volume: 6 ml

detection: 6F8-647

CBP: 60 pM BoNT F4 HC-MBP 12/17/14 (diluted to 100 nM 2/28/17)

titrant: Hu6F15.6 IgG 2/3/17

titration: 7 samples: 20 nM - 20 fM (1:10)

samples:

1) NSB

2-8) titration

Timing(x)

Bead Handling (Custom Beads)

Draw Source	Time (sec)	Volume (uL)	Rate (mL/min)	Stir
Backflush	20	0	0.0000	
Buffer	20	500	1.5000	✓
Particle Reservoir 1	20	333	1.0000	✓
Buffer	30	500	1.0000	
Waste	2	8	0.2500	
Buffer	20	0	0.0000	
Buffer	9	150	1.0000	

Sample Timing

Draw Source	Time (sec)	Volume (uL)	Rate (mL/min)	Time Stamp
Sample Set 1,208-214	1440	6000	0.2500	
Buffer	30	125	0.2500	
Rack 2: Tube 60	120	500	0.2500	
Buffer	30	125	0.2500	
Buffer	90	1500	1.0000	

Analysis(x)

Baseline / Endpoints:
5 to 10 (sec) from beginning
10 to 5 (sec) from end

Ignore	Binding Signal (V)	Concentration
	0.1164	2.00uM
	0.1461	20.00nM
	0.2001	2.00nM
	0.5200	200.00pM
	0.7032	20.00pM
	0.7812	2.00pM
	0.7764	200.00fM
	0.7834	20.00fM

**Kd:**	292.31pM
**Active CBP:**	285.18fM
**CBP % Activity:**	0.48
**Ratio:**	0.0010
**Sig 100%:**	0.77
**NSB:**	0.12
**%Error:**	2.07

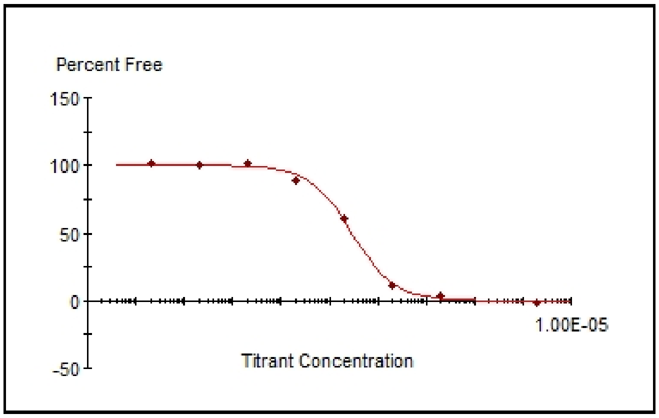

**Kd:**	292.31pM
**95% confidence interval**	
**Kd High:**	337.99pM
**Kd Low:**	197.95pM

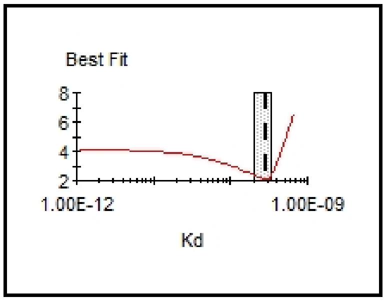

**Active CBP:**	285.18fM
**CBP %Activity:**	0.48
**95% confidence interval**	
**CBP High:**	Greater than 78.94pM
**%Activity:**	Greater than 131.56
**CBP Low:**	Less than 1.03fM
**%Activity:**	Less than 0.00

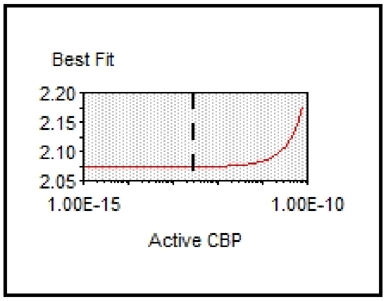

Data Traces(x)

Cycles: 1
Incubation delay (min): 0
Mix Time:

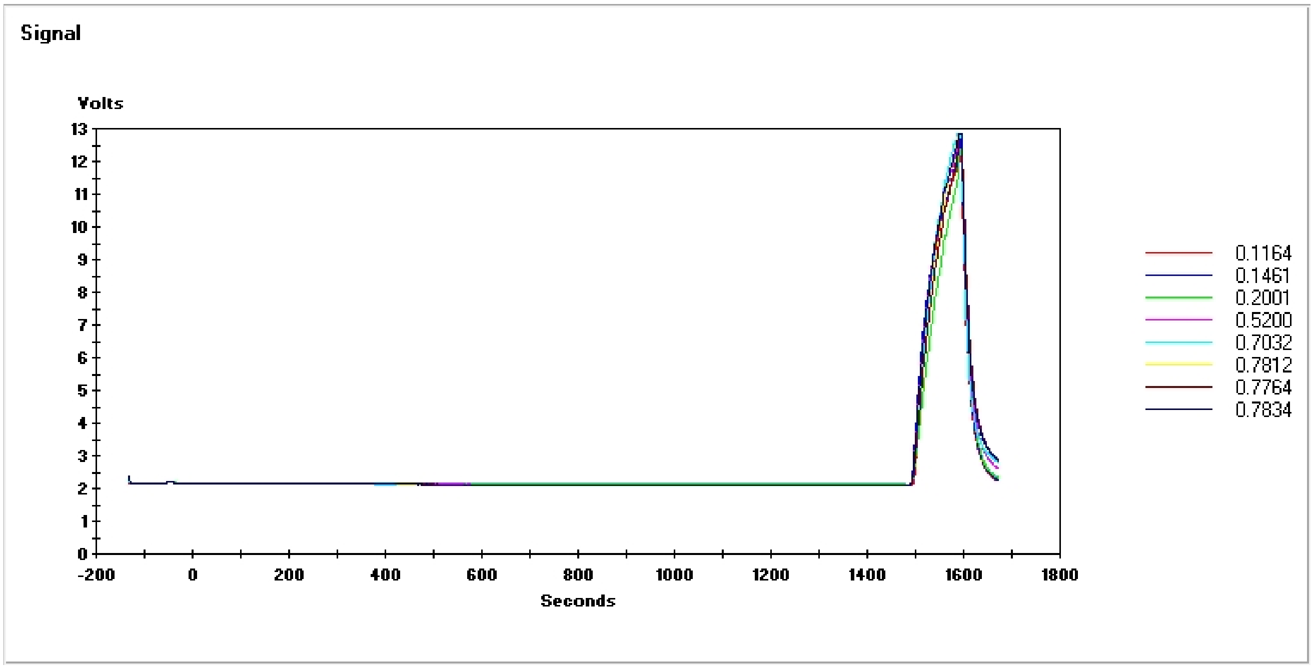

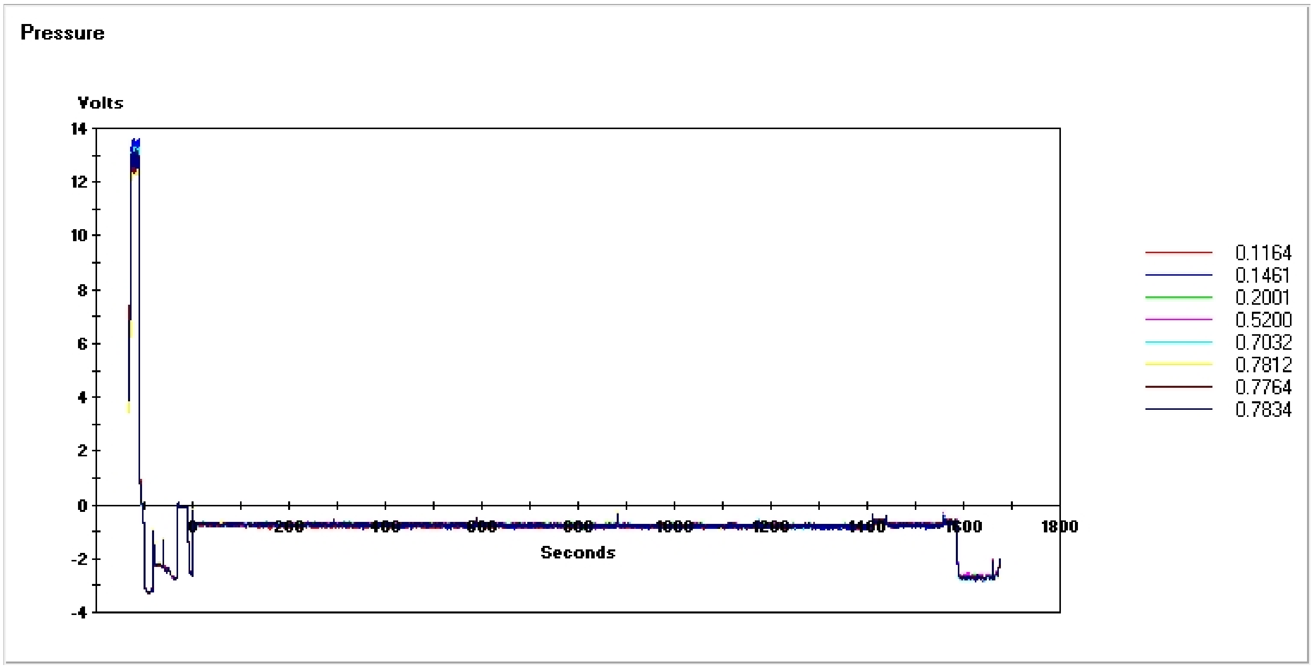

Supplement: S3 Data — (ZIP) [file pone.0273512.s005.zip › IgG KD measurements KinExA/RF Hu6F15.6 vs F4 HC-MBP.pdf]
