# Supplementary material for: Multicolor fluorescence activated cell sorting to generate humanized monoclonal antibody binding seven subtypes of BoNT/F
Source: PLoS One. 2022 Sep 1;17(9):e0273512. doi: 10.1371/journal.pone.0273512 (PMC9436041; doi:10.1371/journal.pone.0273512)

Experiment(x)

**Experiment Name:**	RF hu6F15.1 vs BoNT F4 HC	**Start Time:**	Sat Dec 20 03:16:13 2014
**Experiment Type:**	Equilibrium	**End Time:**	Sat Dec 20 06:13:55 2014
**Constant Binding Partner (CBP)**		**Buffer:**	PBS/BSA
**Molecular Concentration:**	3.00nM	**Label:**	aMBP-647
**Valency:**	1	**Label Conc:**	0
**Binding Site Concentration:**	3.00nM		

Comments(x)

beads: hu6F15.1 11/6/14

sample volume: 3 ml

detection: aMBP-647

receptor: 3 nM BoNT F4 HC 12/17/14

titrant: 6F15.1 IgG 12/14/14

titration: 6 samples: 50 nM - 500 fM (1:10); + BoNT F4 HC only

samples:

1) NSB

2) 100% (BoNT F4 HC only)

3-8) titration of hu6F15.1 IgG

Timing(x)

Bead Handling (Custom Beads)

Draw Source	Time (sec)	Volume (uL)	Rate (mL/min)	Stir
Backflush	20	0	0.0000	
Buffer	20	500	1.5000	✓
Particle Reservoir 1	18	300	1.0000	✓
Buffer	30	500	1.0000	
Waste	2	8	0.2500	
Buffer	20	0	0.0000	
Buffer	9	150	1.0000	

Sample Timing

Draw Source	Time (sec)	Volume (uL)	Rate (mL/min)	Time Stamp
Sample Set 1,222-228	720	3000	0.2500	
Buffer	30	125	0.2500	
Rack 2: Tube 60	120	500	0.2500	
Buffer	30	125	0.2500	
Buffer	90	1500	1.0000	

Analysis(x)

Baseline / Endpoints:
45 to 50 (sec) from beginning
10 to 5 (sec) from end

Ignore	Binding Signal (V)	Concentration
✓	0.4745	0
	1.1222	0
	0.5982	50.00nM
	0.6117	5.00nM
	0.6931	500.00pM
	1.0490	50.00pM
	1.1526	5.00pM
	1.1493	500.00fM

**Kd:**	66.90pM
**Active CBP:**	210.68pM
**CBP % Activity:**	7.02
**Ratio:**	3.1491
**Sig 100%:**	1.14
**NSB:**	0.60
**%Error:**	1.93

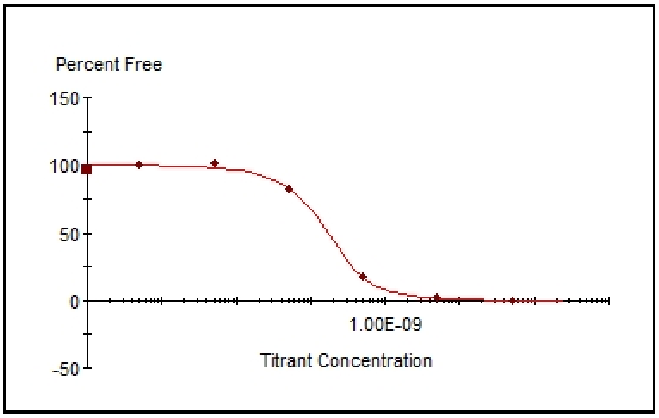

**Kd:**	66.90pM
**95% confidence interval**	
**Kd High:**	96.05pM
**Kd Low:**	43.37pM

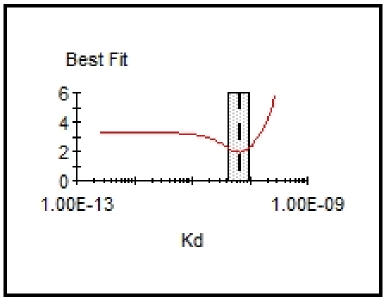

**Active CBP:**	210.68pM
**CBP %Activity:**	7.02
**95% confidence interval**	
**CBP High:**	340.60pM
**%Activity:**	11.35
**CBP Low:**	119.35pM
**%Activity:**	3.98

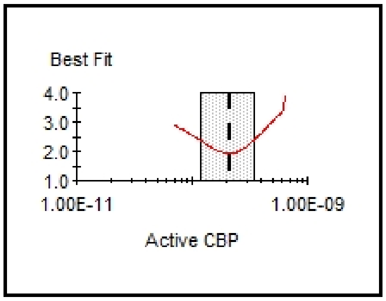

Data Traces(x)

Cycles: 1
Incubation delay (min): 0
Mix Time:

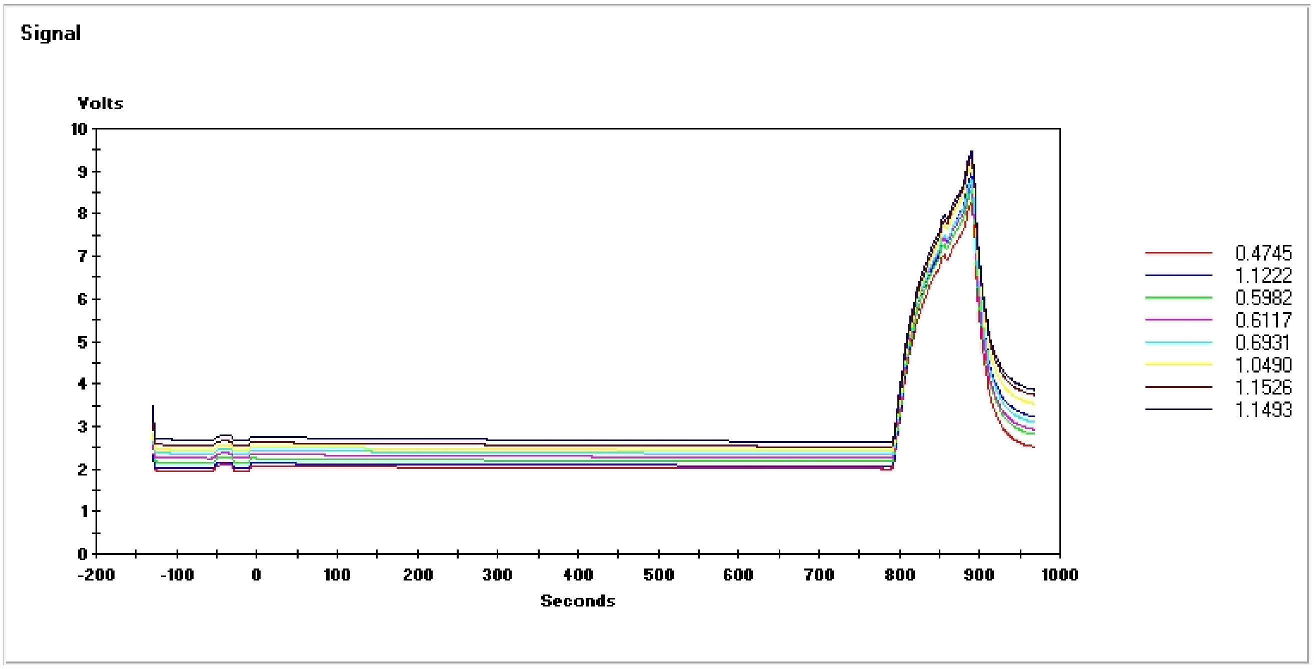

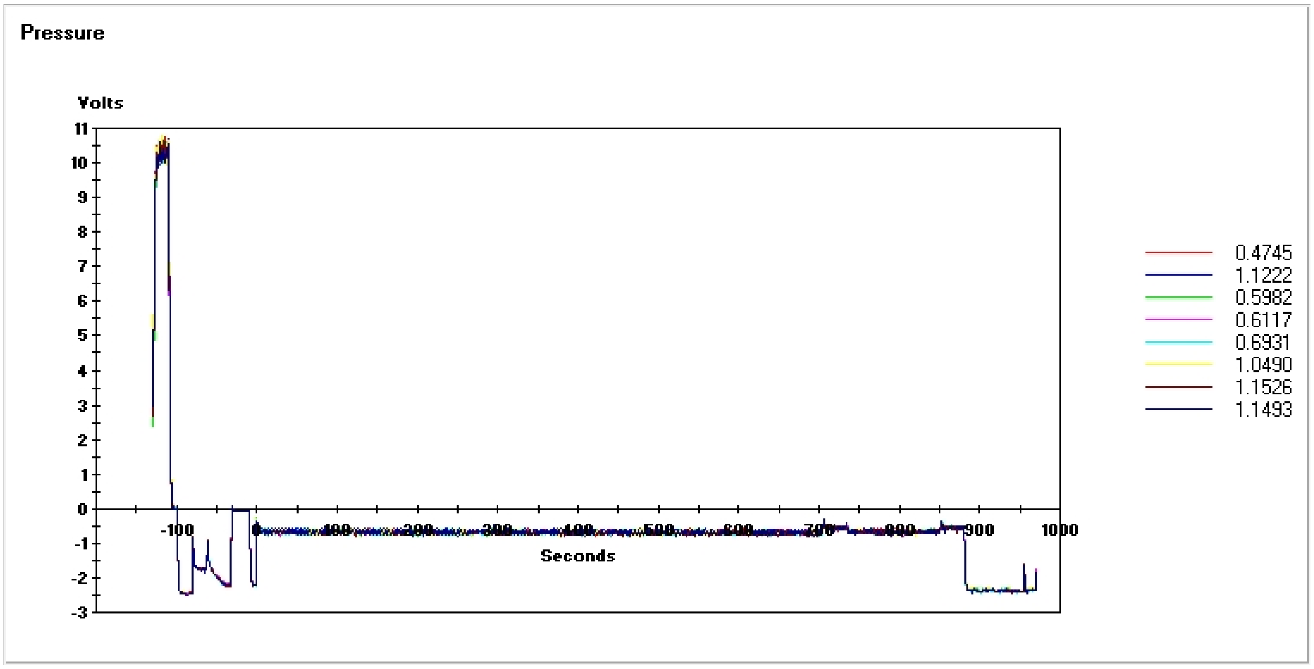

Supplement: S3 Data — (ZIP) [file pone.0273512.s005.zip › IgG KD measurements KinExA/RF hu6F15.1 vs BoNT F4 HC.pdf]
