# Supplementary material for: Multicolor fluorescence activated cell sorting to generate humanized monoclonal antibody binding seven subtypes of BoNT/F
Source: PLoS One. 2022 Sep 1;17(9):e0273512. doi: 10.1371/journal.pone.0273512 (PMC9436041; doi:10.1371/journal.pone.0273512)

Experiment(x)

**Experiment Name:**	RF 28H4 vs F1 HC	**Start Time:**	Wed Aug 02 16:52:05 2017
**Experiment Type:**	Equilibrium	**End Time:**	Wed Aug 02 18:43:49 2017
**Constant Binding Partner (CBP)**		**Buffer:**	PBS/BSA
**Molecular Concentration:**	50.00pM	**Label:**	6F8-647
**Valency:**	1	**Label Conc:**	0
**Binding Site Concentration:**	50.00pM		

Comments(x)

beads: Hu6F15.3 8/2/17

sample volume: 1000 ul

detection: 6F8-647

CBP: 50 pM BoNT F1 HC 12/17/14 (diluted to 100 nM 2/28/17)

titrant: 28H4 IgG 11/3/16

titration: 7 samples: 100 nM - 100 fM (1:10)

samples:

1) NSB

2-8) titration

Timing(x)

Bead Handling (Custom Beads)

Draw Source	Time (sec)	Volume (uL)	Rate (mL/min)	Stir
Backflush	20	0	0.0000	
Buffer	20	500	1.5000	✓
Particle Reservoir 1	18	300	1.0000	✓
Buffer	30	500	1.0000	
Waste	2	8	0.2500	
Buffer	20	0	0.0000	
Buffer	9	150	1.0000	

Sample Timing

Draw Source	Time (sec)	Volume (uL)	Rate (mL/min)	Time Stamp
Sample Set 1,301-307	240	1000	0.2500	
Buffer	30	125	0.2500	
Rack 2: Tube 60	120	500	0.2500	
Buffer	30	125	0.2500	
Buffer	90	1500	1.0000	

Analysis(x)

Baseline / Endpoints:
5 to 10 (sec) from beginning
10 to 5 (sec) from end

Ignore	Binding Signal (V)	Concentration
✓	0.0933	NSB
	0.1050	100.00nM
	0.1112	10.00nM
	0.1527	1.00nM
	0.2144	100.00pM
	0.2240	10.00pM
	0.2338	1.00pM
	0.2292	100.00fM

**Kd:**	584.84pM
**Active CBP:**	116.31pM
**CBP % Activity:**	232.63
**Ratio:**	0.1989
**Sig 100%:**	0.23
**NSB:**	0.10
**%Error:**	1.65

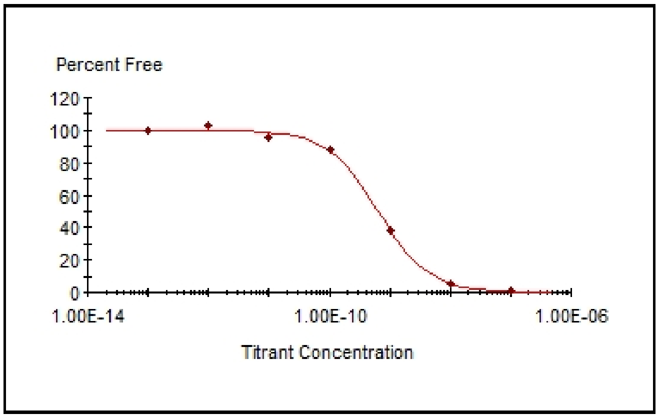

**Kd:**	584.84pM
**95% confidence interval**	
**Kd High:**	716.02pM
**Kd Low:**	395.50pM

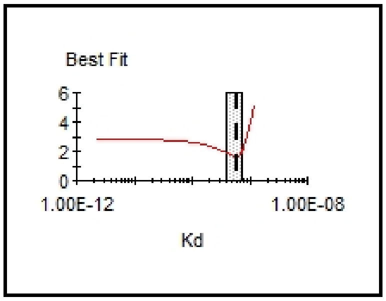

**Active CBP:**	116.31pM
**CBP %Activity:**	232.63
**95% confidence interval**	
**CBP High:**	705.58pM
**%Activity:**	1411.16
**CBP Low:**	Less than 420.20fM
**%Activity:**	Less than 0.84

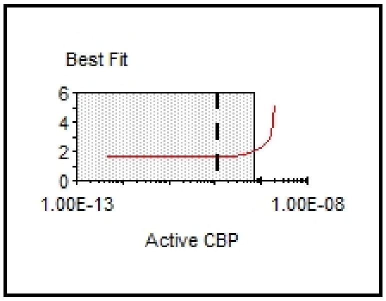

Data Traces(x)

Cycles: 1
Incubation delay (min): 0
Mix Time:

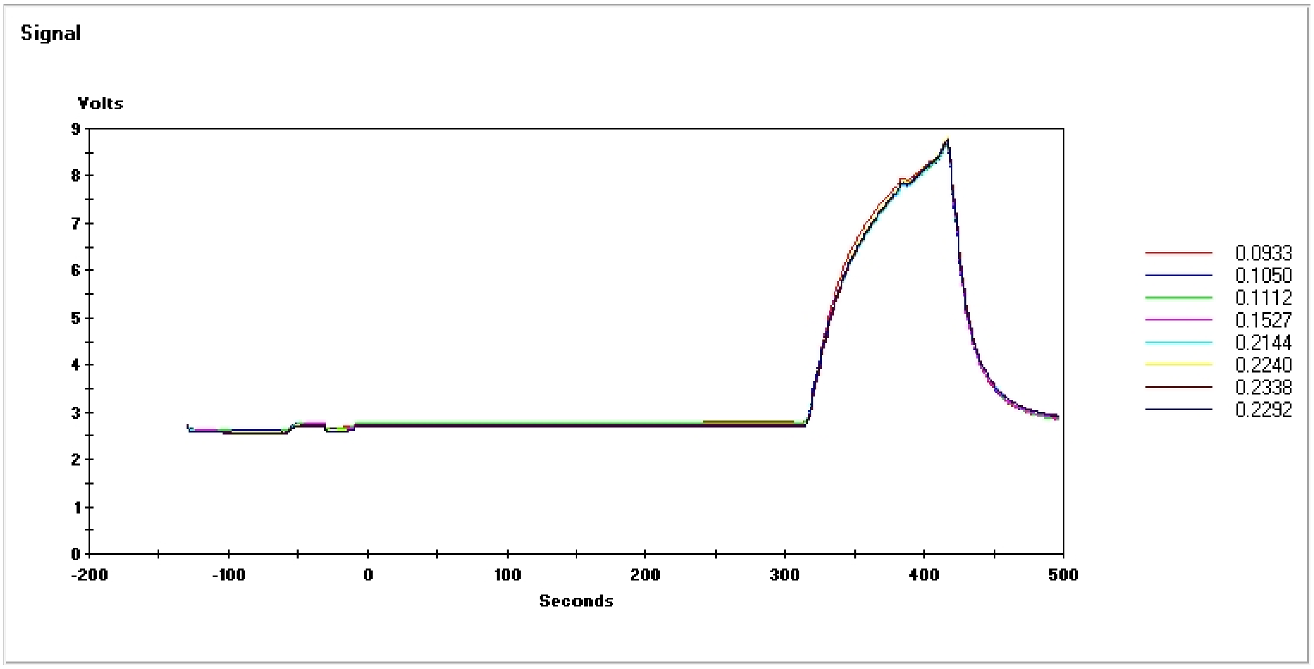

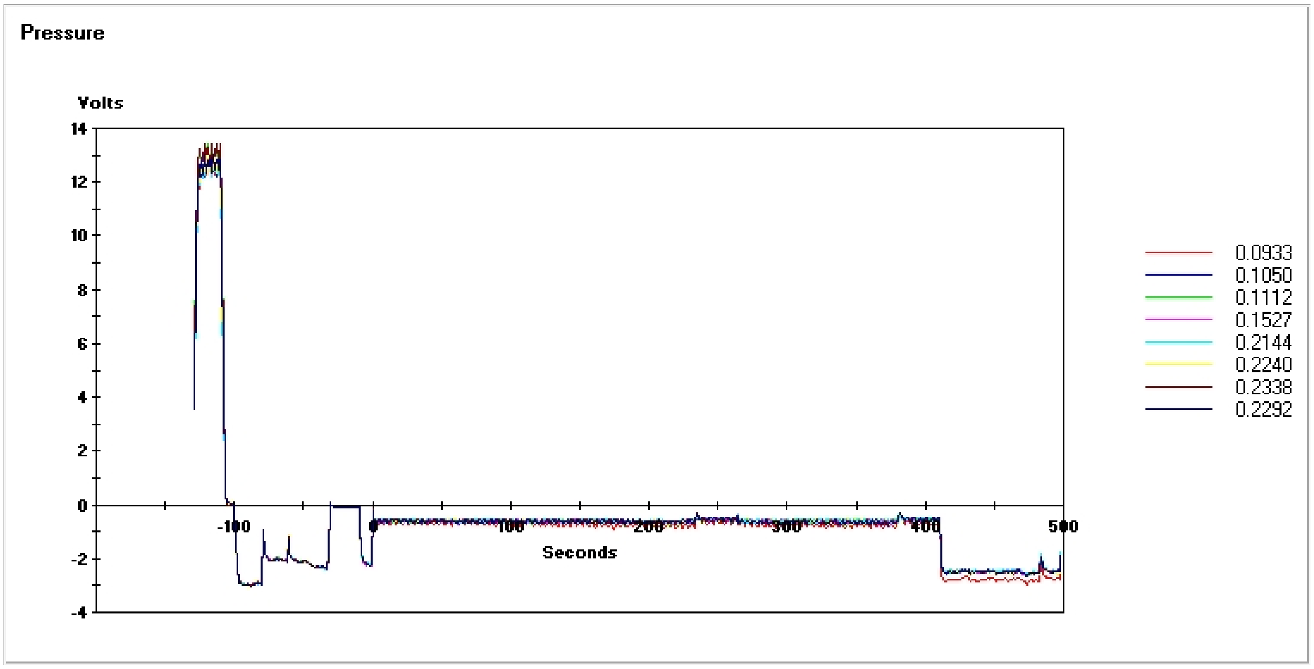

Supplement: S3 Data — (ZIP) [file pone.0273512.s005.zip › IgG KD measurements KinExA/RF 28H4 vs F1 HC.pdf]
