# Supplementary material for: Multicolor fluorescence activated cell sorting to generate humanized monoclonal antibody binding seven subtypes of BoNT/F
Source: PLoS One. 2022 Sep 1;17(9):e0273512. doi: 10.1371/journal.pone.0273512 (PMC9436041; doi:10.1371/journal.pone.0273512)

Experiment(x)

**Experiment Name:**	RF Hu6F15.1 vs F5 HNHC-MBP{3}	**Start Time:**	Fri Oct 06 19:44:50 2017
**Experiment Type:**	Equilibrium	**End Time:**	Fri Oct 06 20:56:34 2017
**Constant Binding Partner (CBP)**		**Buffer:**	PBS/BSA
**Molecular Concentration:**	5.00nM	**Label:**	6F5.4-647
**Valency:**	1	**Label Conc:**	0
**Binding Site Concentration:**	5.00nM		

Comments(x)

beads: Hu6F15.3 9/8/17

sample volume: 500 ul

detection: 6F5.4-647

CBP: 5 nM BoNT F5 HNHC-MBP 11/16/15

titrant: Hu6F15.1 IgG 3/21/15

titration: 5 samples: 400 nM - 40 pM (1:10)

samples:

1) NSB

2-6) titration

Timing(x)

Bead Handling (Custom Beads)

Draw Source	Time (sec)	Volume (uL)	Rate (mL/min)	Stir
Backflush	20	0	0.0000	
Buffer	20	500	1.5000	✓
Particle Reservoir 1	18	300	1.0000	✓
Buffer	30	500	1.0000	
Waste	2	8	0.2500	
Buffer	20	0	0.0000	
Buffer	9	150	1.0000	

Sample Timing

Draw Source	Time (sec)	Volume (uL)	Rate (mL/min)	Time Stamp
Sample Set 1,301-305	120	500	0.2500	
Buffer	30	125	0.2500	
Rack 2: Tube 60	120	500	0.2500	
Buffer	30	125	0.2500	
Buffer	90	1500	1.0000	

Analysis(x)

Baseline / Endpoints:
185 to 190 (sec) from beginning
10 to 5 (sec) from end

**Ignore**	**Binding Signal (V)**	**Concentration**
✓	0.0228	NSB
	0.0342	400.00nM
	0.0402	40.00nM
	0.1015	4.00nM
	0.2315	400.00pM
	0.2461	40.00pM

**Kd:**	550.51pM
**Active CBP:**	4.03nM
**CBP % Activity:**	80.62
**Ratio:**	7.3225
**Sig 100%:**	0.25
**NSB:**	0.04
**%Error:**	0.49

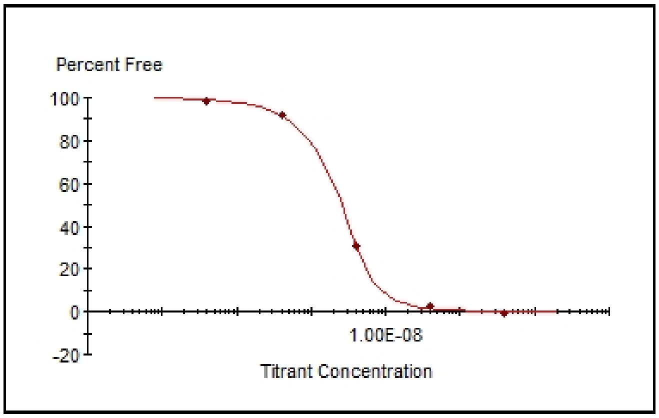

Kd: 550.51pM
95% confidence interval

**Kd High:**	739.22pM
**Kd Low:**	339.32pM

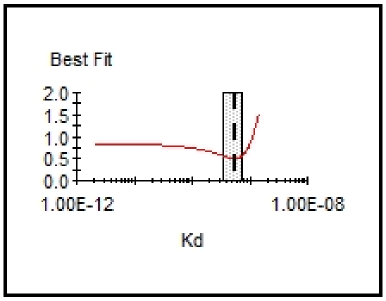

**Active CBP:**	4.03nM
**CBP %Activity:**	80.62

95% confidence interval

**CBP High:**	4.89nM
**%Activity:**	97.90
**CBP Low:**	3.29nM
**%Activity:**	65.82

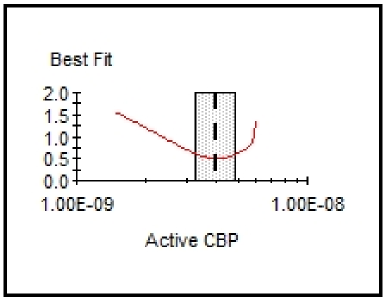

Data Traces(x)

Cycles: 1
Incubation delay (min): 120
Mix Time:

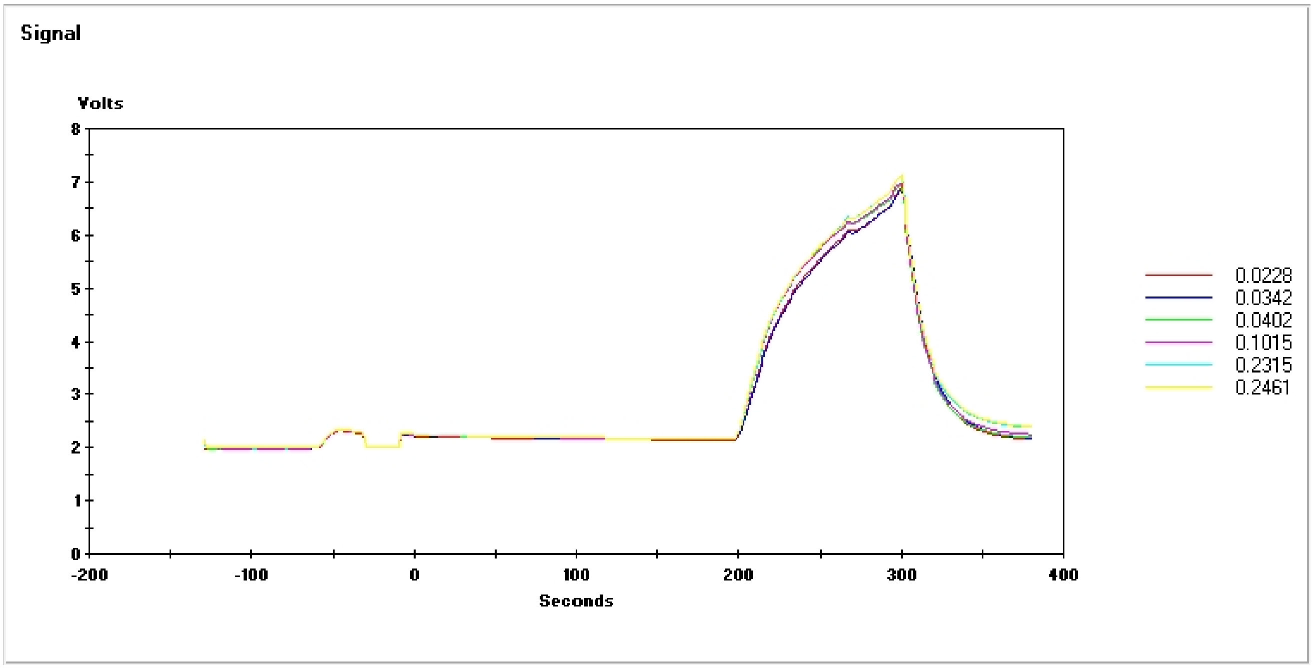

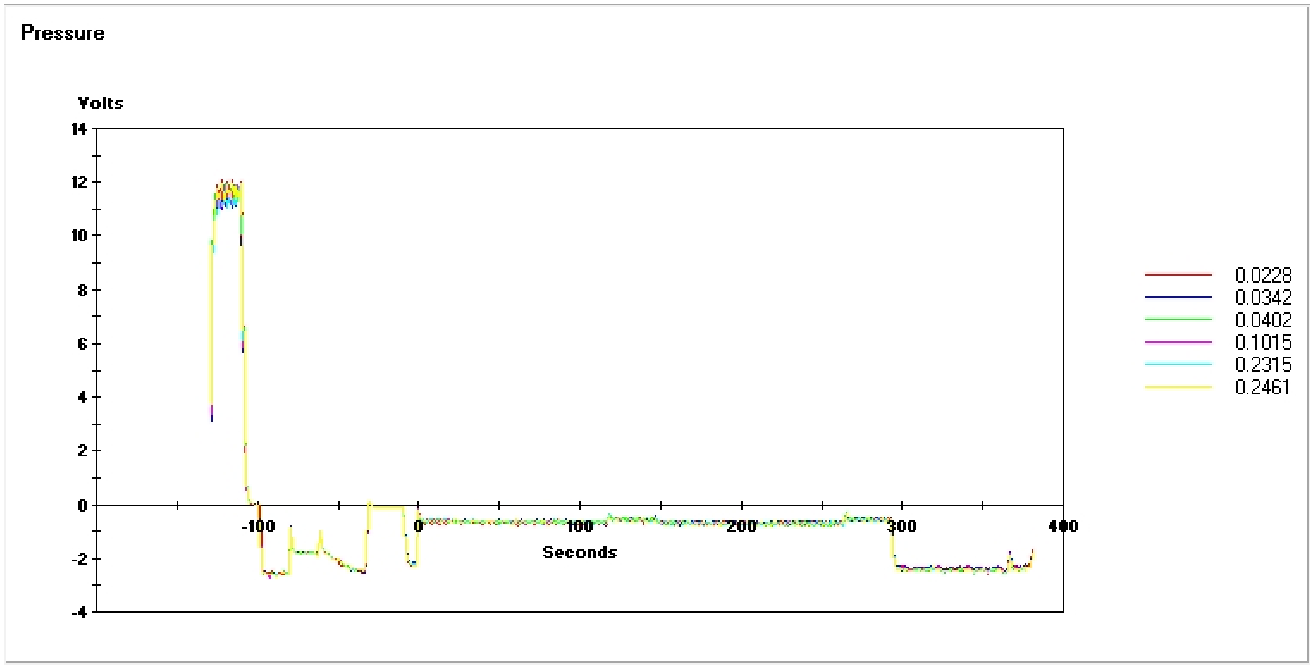

Supplement: S3 Data — (ZIP) [file pone.0273512.s005.zip › IgG KD measurements KinExA/RF Hu6F15.1 vs F5 HNHC-MBP{3}.pdf]
