# Supplementary material for: Multicolor fluorescence activated cell sorting to generate humanized monoclonal antibody binding seven subtypes of BoNT/F
Source: PLoS One. 2022 Sep 1;17(9):e0273512. doi: 10.1371/journal.pone.0273512 (PMC9436041; doi:10.1371/journal.pone.0273512)

Experiment(x)

**Experiment Name:**	RF 28H4 vs F1	**Start Time:**	Wed Sep 13 19:10:52 2017
**Experiment Type:**	Equilibrium	**End Time:**	Wed Sep 13 23:45:29 2017
**Constant Binding Partner (CBP)**		**Buffer:**	PBS/BSA
**Molecular Concentration:**	80.00pM	**Label:**	6F5.4-647
**Valency:**	1	**Label Conc:**	0
**Binding Site Concentration:**	80.00pM		

Comments(x)

beads: Hu6F15.3 8/28/17

sample volume: 6 ml

detection: 6F5.4-647

CBP: 80 pM BoNT F1 holotoxin 100251 9/12/17

titrant: 28H4 IgG 11/3/16

titration: 7 samples: 100 nM - 100 fM (1:10)

samples:

1) NSB

2-8) titration
beads: Hu6F15.3 9/8/17

Timing(x)

Bead Handling (Custom Beads)

Draw Source	Time (sec)	Volume (uL)	Rate (mL/min)	Stir
Backflush	20	0	0.0000	
Buffer	20	500	1.5000	✓
Particle Reservoir 2	18	300	1.0000	✓
Buffer	30	500	1.0000	
Waste	2	8	0.2500	
Buffer	20	0	0.0000	
Buffer	9	150	1.0000	

Sample Timing

Draw Source	Time (sec)	Volume (uL)	Rate (mL/min)	Time Stamp
Sample Set 1,201-207	1440	6000	0.2500	
Buffer	30	125	0.2500	
Rack 2: Tube 59	120	500	0.2500	
Buffer	30	125	0.2500	
Buffer	90	1500	1.0000	

Analysis(x)

Baseline / Endpoints:
5 to 10 (sec) from beginning
10 to 5 (sec) from end

Binding

Ignore	Signal (V)	Concentration
	0.0062	10.00uM
	0.0149	100.00nM
	0.0806	10.00nM
	0.1979	1.00nM
	0.2496	100.00pM
	0.2553	10.00pM
	0.2635	1.00pM
	0.2717	100.00fM

Kd: 3.48nM
Active CBP: 3.38pM
CBP %Activity: 4.23
Ratio: 0.0010
Sig 100%: 0.26
NSB: 0.01
%Error: 2.28

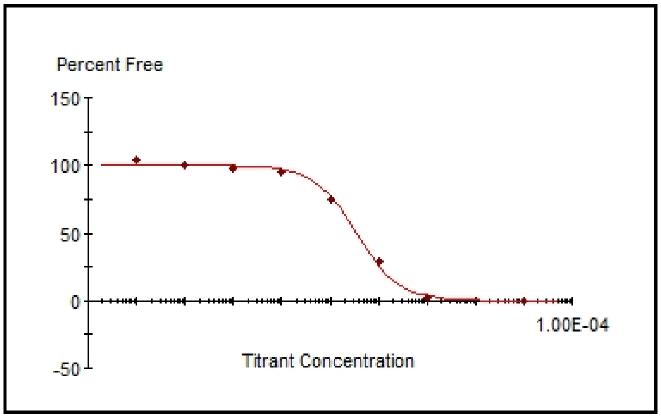

Kd: 3.48nM
95% confidence interval
Kd High: 4.17nM
Kd Low: 2.89nM

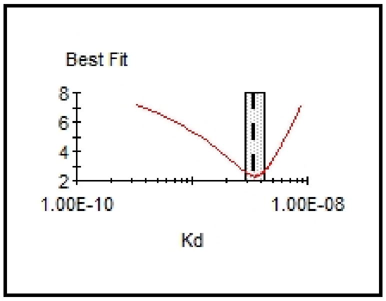

Active CBP: 3.38pM
CBP %Activity: 4.23
95% confidence interval
CBP High: Greater than 936.79pM
%Activity: Greater than 1170.99
CBP Low: Less than 12.23fM
%Activity: Less than 0.02

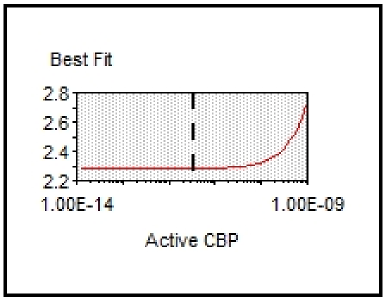

Data Traces(x)

Cycles: 1
Incubation delay (min): 0
Mix Time:

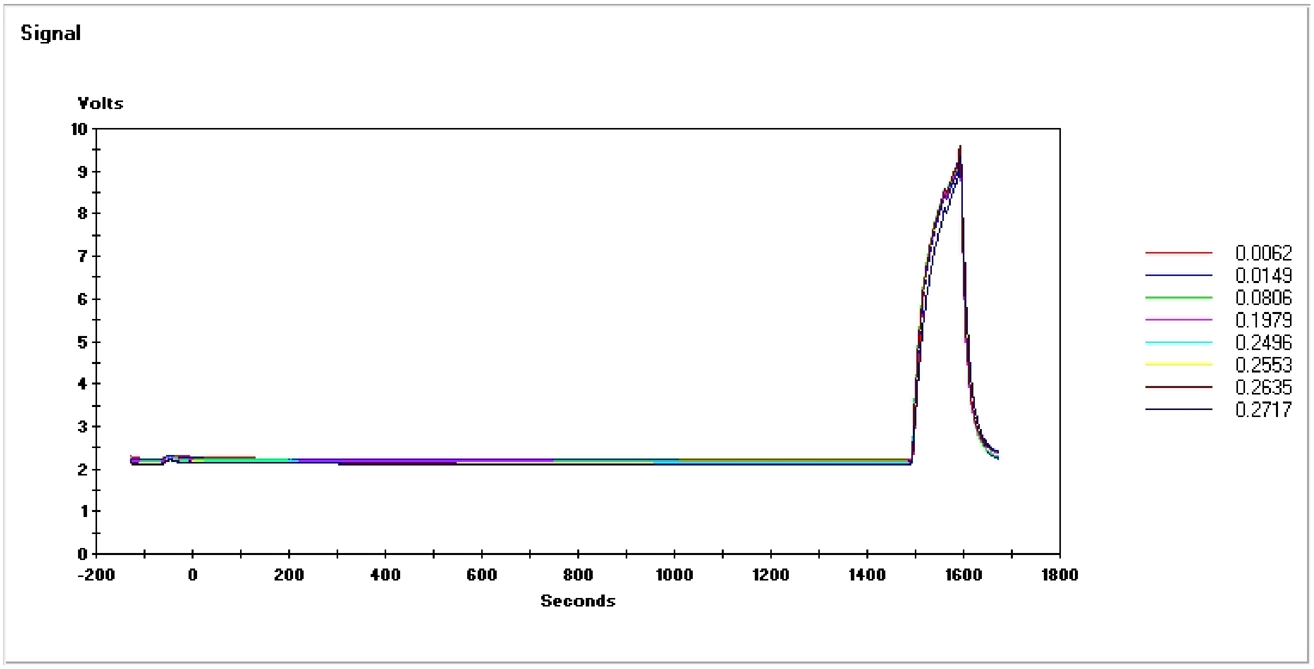

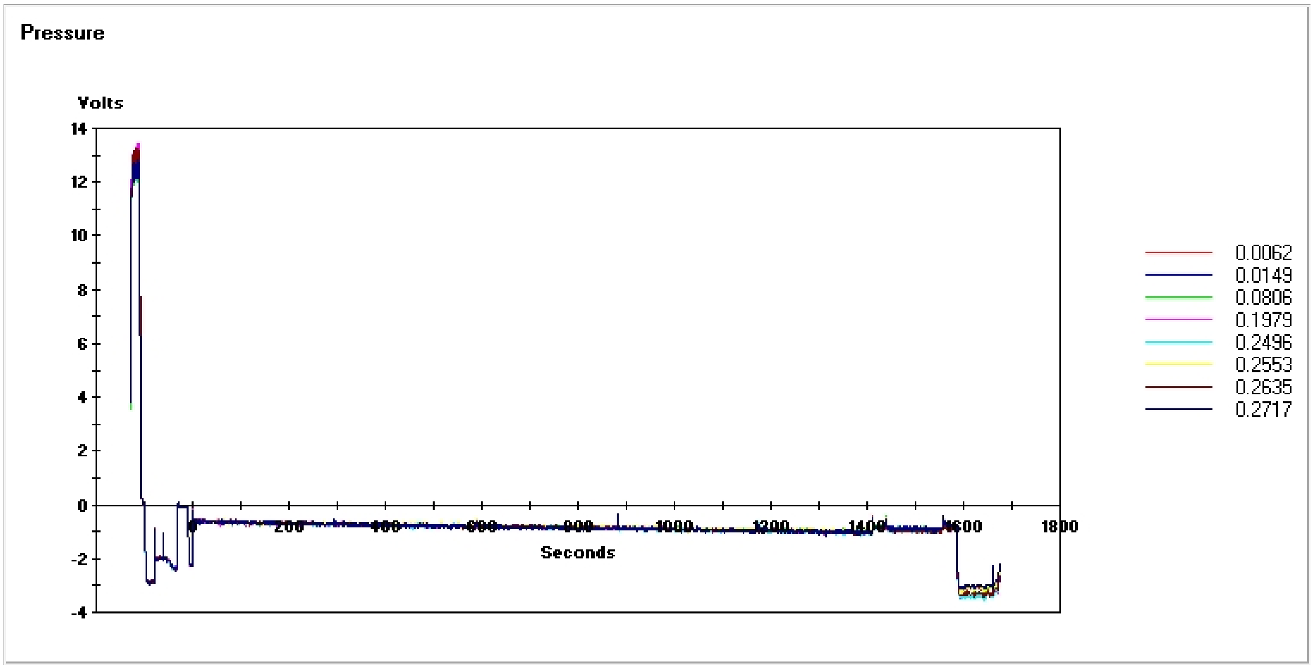

Supplement: S3 Data — (ZIP) [file pone.0273512.s005.zip › IgG KD measurements KinExA/RF 28H4 vs F1.pdf]
