# Supplementary material for: Multicolor fluorescence activated cell sorting to generate humanized monoclonal antibody binding seven subtypes of BoNT/F
Source: PLoS One. 2022 Sep 1;17(9):e0273512. doi: 10.1371/journal.pone.0273512 (PMC9436041; doi:10.1371/journal.pone.0273512)

Experiment(x)

**Experiment Name:**	RF Hu6F15.6 vs F7 HC-MBP{3}	**Start Time:**	Tue Sep 12 11:03:08 2017
**Experiment Type:**	Equilibrium	**End Time:**	Tue Sep 12 15:37:44 2017
**Constant Binding Partner (CBP)**		**Buffer:**	PBS/BSA
**Molecular Concentration:**	400.00pM	**Label:**	6F3-647
**Valency:**	1	**Label Conc:**	0
**Binding Site Concentration:**	400.00pM		

Comments(x)

beads: Hu6F15.3 9/8/17

sample volume: 6 ml

detection: 6F3-647

CBP: 400 pM BoNT F7 HC-MBP 12/17/14

titrant: Hu6F15.6 IgG 2/3/17

titration: 7 samples: 40 nM - 40 pM (1:10)

samples:

1) NSB

2-8) titration

Timing(x)

Bead Handling (Custom Beads)

Draw Source	Time (sec)	Volume (uL)	Rate (mL/min)	Stir
Backflush	20	0	0.0000	
Buffer	20	500	1.5000	✓
Particle Reservoir 1	18	300	1.0000	✓
Buffer	30	500	1.0000	
Waste	2	8	0.2500	
Buffer	20	0	0.0000	
Buffer	9	150	1.0000	

Sample Timing

Draw Source	Time (sec)	Volume (uL)	Rate (mL/min)	Time Stamp
Sample Set 1,201-207	1440	6000	0.2500	
Buffer	30	125	0.2500	
Rack 2: Tube 60	120	500	0.2500	
Buffer	30	125	0.2500	
Buffer	90	1500	1.0000	

Analysis(x)

Baseline / Endpoints:
1400 to 1405 (sec) from beginning
10 to 5 (sec) from end

Ignore	Binding Signal (V)	Concentration
✓	0.0671	NSB
	0.0638	40.00nM
	0.0688	4.00nM
	0.1318	400.00pM
	0.5641	40.00pM
	0.6371	4.00pM
	0.6349	400.00fM
	0.6574	40.00fM

**Kd:**	22.85pM
**Active CBP:**	255.55pM
**CBP % Activity:**	63.89
**Ratio:**	11.1852
**Sig 100%:**	0.65
**NSB:**	0.06
**%Error:**	0.94

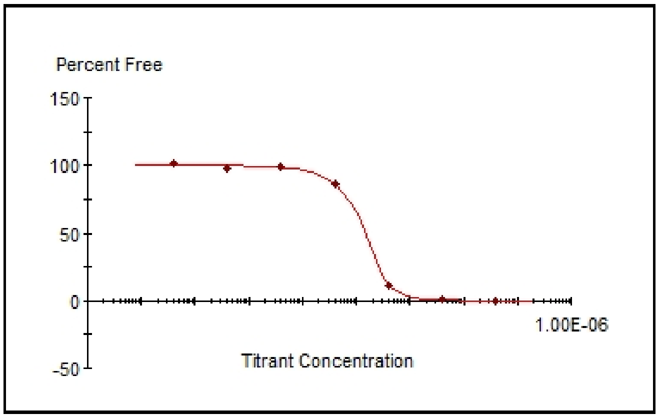

Kd: 22.85pM
95% confidence interval
Kd High: 29.99pM
Kd Low: 16.39pM

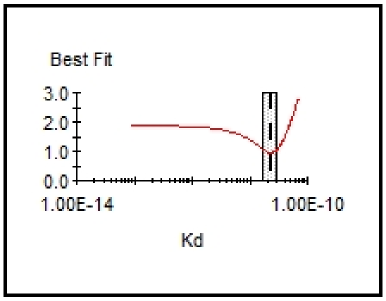

Active CBP: 255.55pM
CBP %Activity: 63.89
95% confidence interval
CBP High: 314.94pM
%Activity: 78.73
CBP Low: 211.04pM
%Activity: 52.76

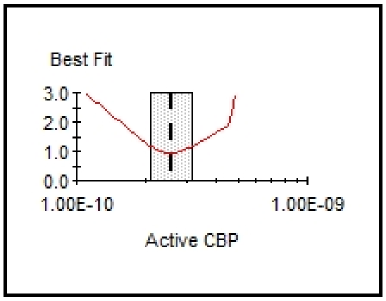

Data Traces(x)

Cycles: 1
Incubation delay (min): 0
Mix Time:

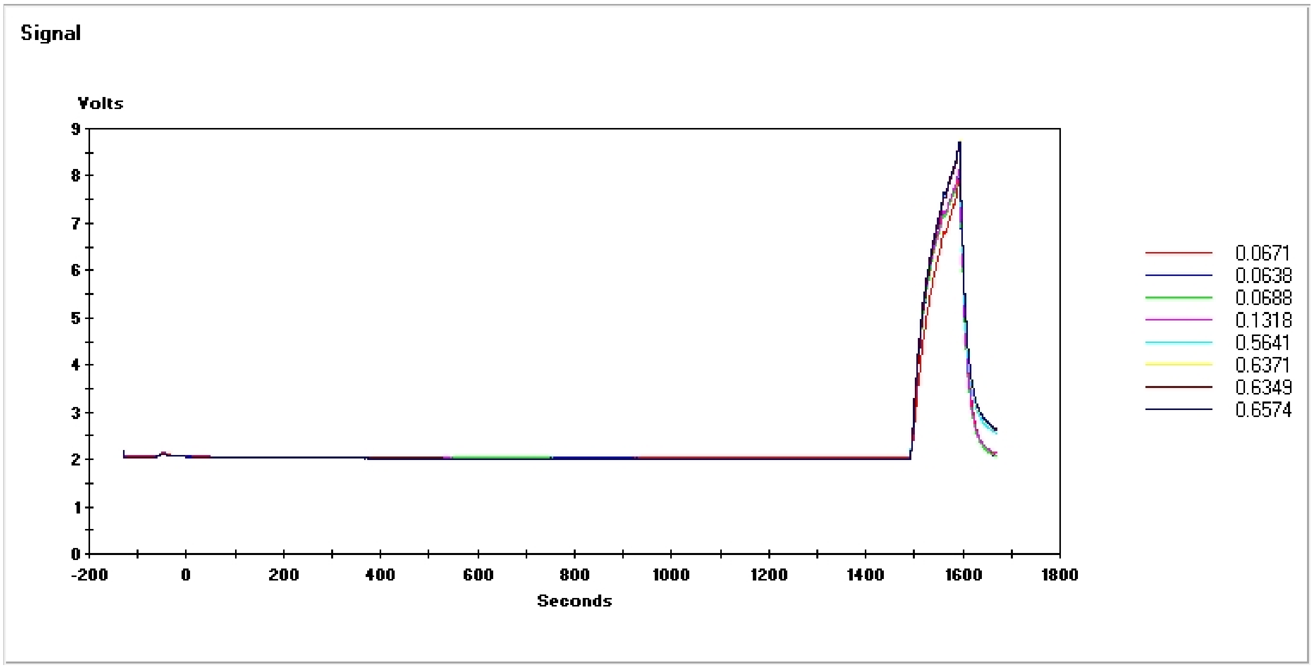

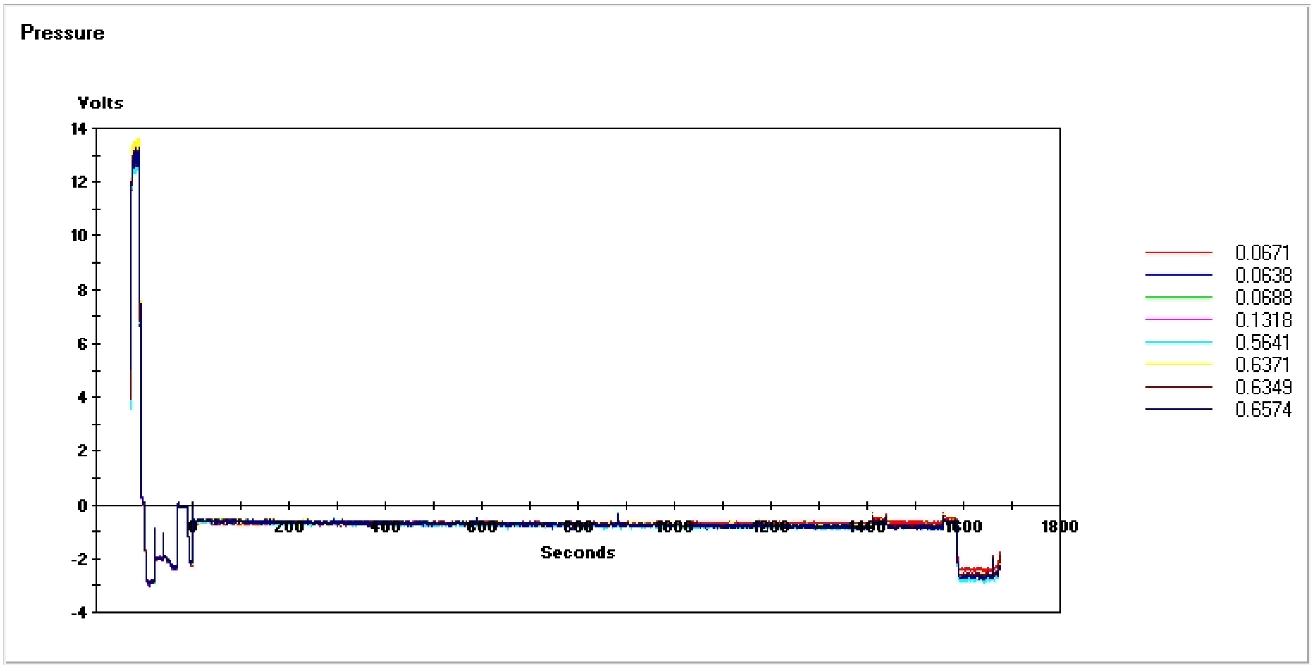

Supplement: S3 Data — (ZIP) [file pone.0273512.s005.zip › IgG KD measurements KinExA/RF Hu6F15.6 vs F7 HC-MBP{3}.pdf]
